# Altering the Properties of Spiropyran Switches Using Coordination Cages with Different Symmetries

**DOI:** 10.1021/jacs.2c08901

**Published:** 2022-11-15

**Authors:** Jinhua Wang, Liat Avram, Yael Diskin-Posner, Michał J. Białek, Wojciech Stawski, Moran Feller, Rafal Klajn

**Affiliations:** 1 Department of Organic Chemistry, Weizmann Institute of Science, Rehovot 76100, Israel; 2 Department of Chemical Research Support, Weizmann Institute of Science, Rehovot 76100, Israel; 3 Department of Chemistry, University of Wrocław, 14 F. Joliot-Curie Street, 50383 Wrocław, Poland

## Abstract

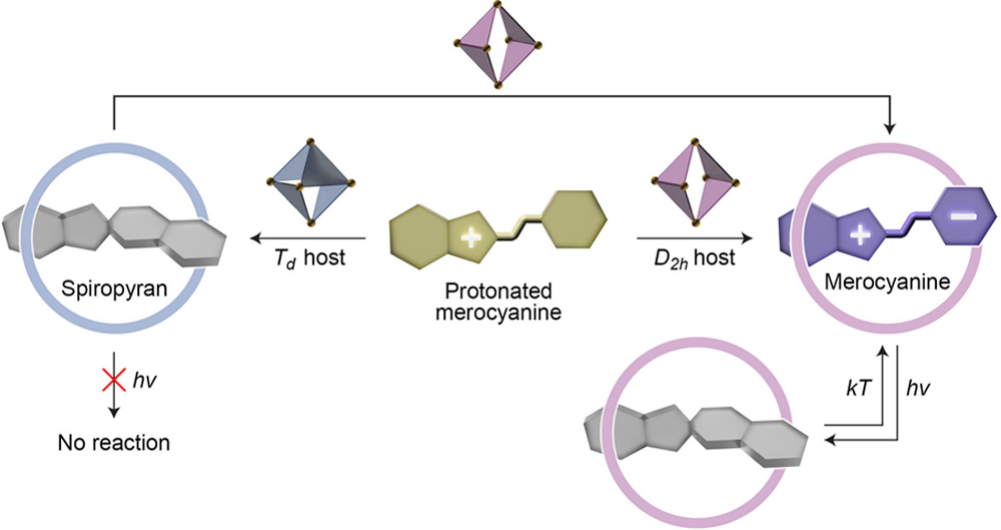

Molecular confinement effects can profoundly alter the physicochemical properties of the confined species. A plethora of organic molecules were encapsulated within the cavities of supramolecular hosts, and the impact of the cavity size and polarity was widely investigated. However, the extent to which the properties of the confined guests can be affected by the symmetry of the cage—which dictates the shape of the cavity—remains to be understood. Here we show that cage symmetry has a dramatic effect on the equilibrium between two isomers of the encapsulated spiropyran guests. Working with two Pd-based coordination cages featuring similarly sized but differently shaped hydrophobic cavities, we found a highly selective stabilization of the isomer whose shape matches that of the cavity of the cage. A *T*_*d*_-symmetric cage stabilized the spiropyrans’ colorless form and rendered them photochemically inert. In contrast, a *D*_2*h*_-symmetric cage favored the colored isomer, while maintaining reversible photoswitching between the two states of the encapsulated spiropyrans. We also show that the switching kinetics strongly depend on the substitution pattern on the spiropyran scaffold. This finding was used to fabricate a time-sensitive information storage medium with tunable lifetimes of the encoded messages.

## Introduction

Confining molecules within spaces not much larger than the molecules themselves can significantly affect their properties.^1−8^ Nature is rich in examples of how molecular confinement effects can dramatically increase the rate^9^ and alter the course of chemical reactions^10^—or, vice versa, stabilize highly reactive species (such as sulfenic acids, RS–OH^11^)—all within protein matrices. In synthetic systems, cage-like molecules formed by metal–ligand coordination (i.e., coordination cages) provide an attractive platform to study the effect of confinement on chemical reactivity.^12^ In a seminal study, white phosphorus (P_4_) was rendered chemically stable within the cavity of a water-soluble, Fe-based coordination cage.^13^ Similarly, the radical initiator AIBN was found to remain stable within (but could be released “on demand” from) a Pd-based cage.^14^ Coordination cages can also have contrasting effects on the free energies of two isomers of switchable molecules, thus stabilizing otherwise metastable isomers.^15−19^

Furthermore, coordination cages (and other molecular capsules^20,21^) can alter the course of chemical reactions. Recently, binding within a Pd-based cage was found to induce an unusual conformation of a 2-biphenylacetylene, forcing it to undergo an unexpected 5-*endo*-*dig* cyclization.^22^ In another example, hydrogenation of a triene proceeded in a highly regioselective fashion inside a Ga-based coordination cage.^23^ During the past several years, increasing attention has been devoted to the effect of confinement on the photoresponsive properties of the encapsulated guests.^24^ However, the impact of cage architecture (symmetry)—which determines the shape of the cavity—on the behavior of photoresponsive guests has so far remained unexplored.

**Figure 1:**
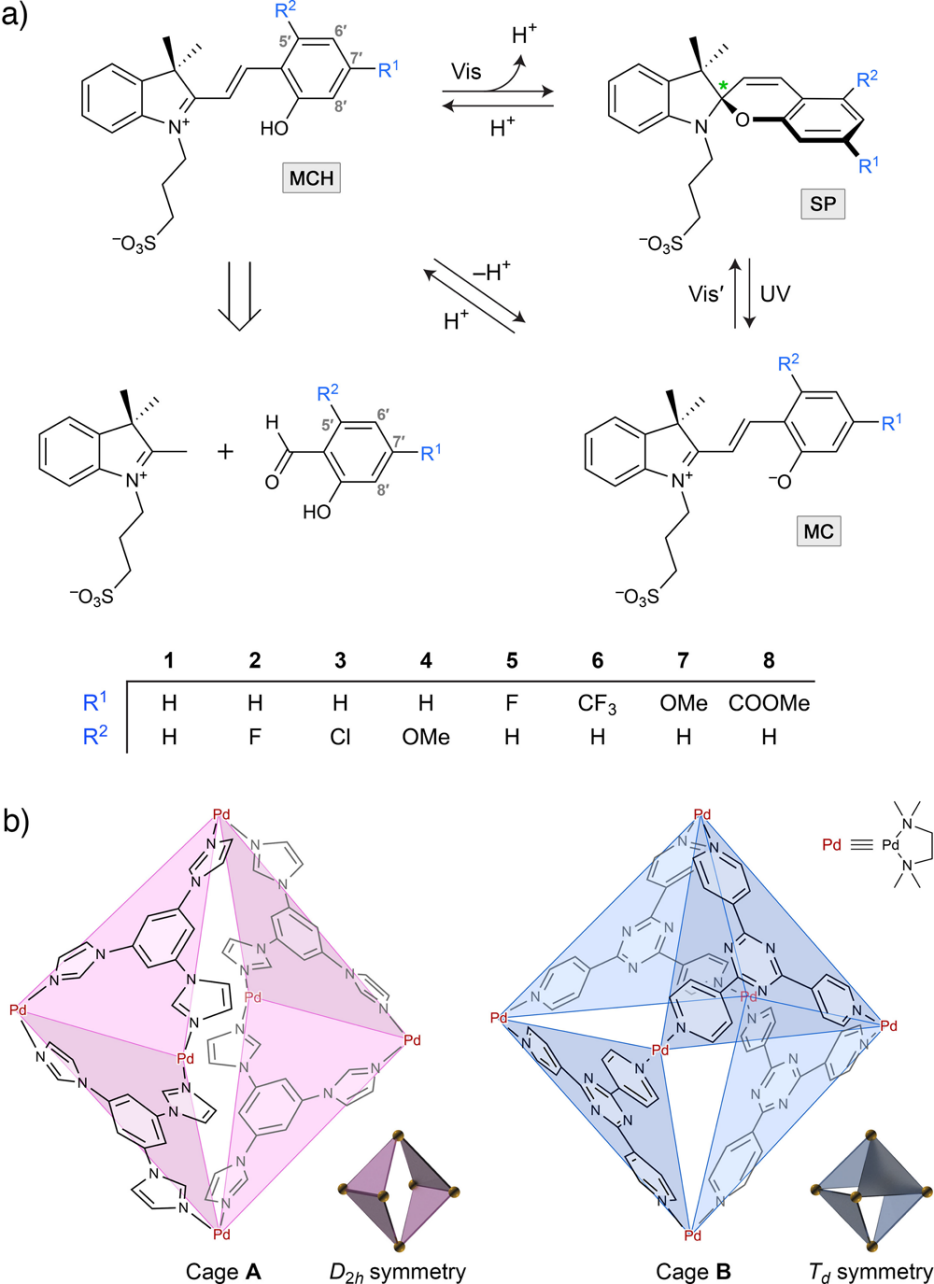
(a) Reversible transformations between the spiropyran’s MCH^+^, SP, and MC forms and (bottom left) the building blocks for the modular synthesis of spiropyran-based photoacids. The table at the bottom lists the eight spiropyrans investigated in this study. (b) The two Pd^II^_6_L_4_ coordination cages used in this study: the *D*_2*h*_-symmetric Mukherjee cage^46^ (cage **A**; left) and the *T*_*d*_-symmetric Fujita cage^48^ (cage **B**; right). Both cages have a charge of +12 (counterions = 12 NO_3_^–^) and *N*,*N*,*N*′,*N*′-tetramethylethylenediamine (TMEDA) as the ancillary ligand.

Here, we set out to investigate the effect of cavity shape on the properties of spiropyran switches. Owing to their ability to respond to multiple external stimuli, spiropyrans have emerged as arguably the most versatile switchable molecules.^25,26^ Depending on the environmental conditions (e.g., pH, solvent polarity, the wavelength of the incident light), a spiropyran switch can adopt one of several states, including the closed-ring isomer (SP in Figure 1a), the open-ring isomer (often referred to as “merocyanine”; MC in Figure 1a), and the protonated open-ring form (protonated merocyanine; MCH in Figure 1a).^27^ For example, nonpolar solvents stabilize the SP form, UV light and polar media favor MC, and low-pH environments shift the equilibrium toward MCH. In 2011, Liao and co-workers reported compound **1** (Figure 1a), a self-protonating spiropyran appended with a 3-sulfopropyl chain, which has a high tendency to exist in the MCH form.^28^ Upon exposure to blue light, the MCH form of **1** undergoes cyclization, accompanied by the release of a proton. In the dark, the reverse reaction occurs spontaneously (Figure 1a, top); therefore, solutions of **1** exhibit a lower pH under continuous light irradiation. Over the past several years, compound **1** has been used for an impressive repertoire of functions, including guiding the assembly of coordination cages^29^ and one-dimensional coordination polymers,^30^ operating pH-responsive molecular switches^31,32^ and DNA-origami-based plasmonic assemblies,^33^ controlling the assembly state of gold nanocrystals^34^ and microgel particles,^35^ tuning the activity of microbial fuel cells^36^ and the rate of ATP production by chloroplasts,^37^ and modulating permeability through the walls of vesicular nanoreactors,^38^ among other functions.^39−45^

**Figure 2:**
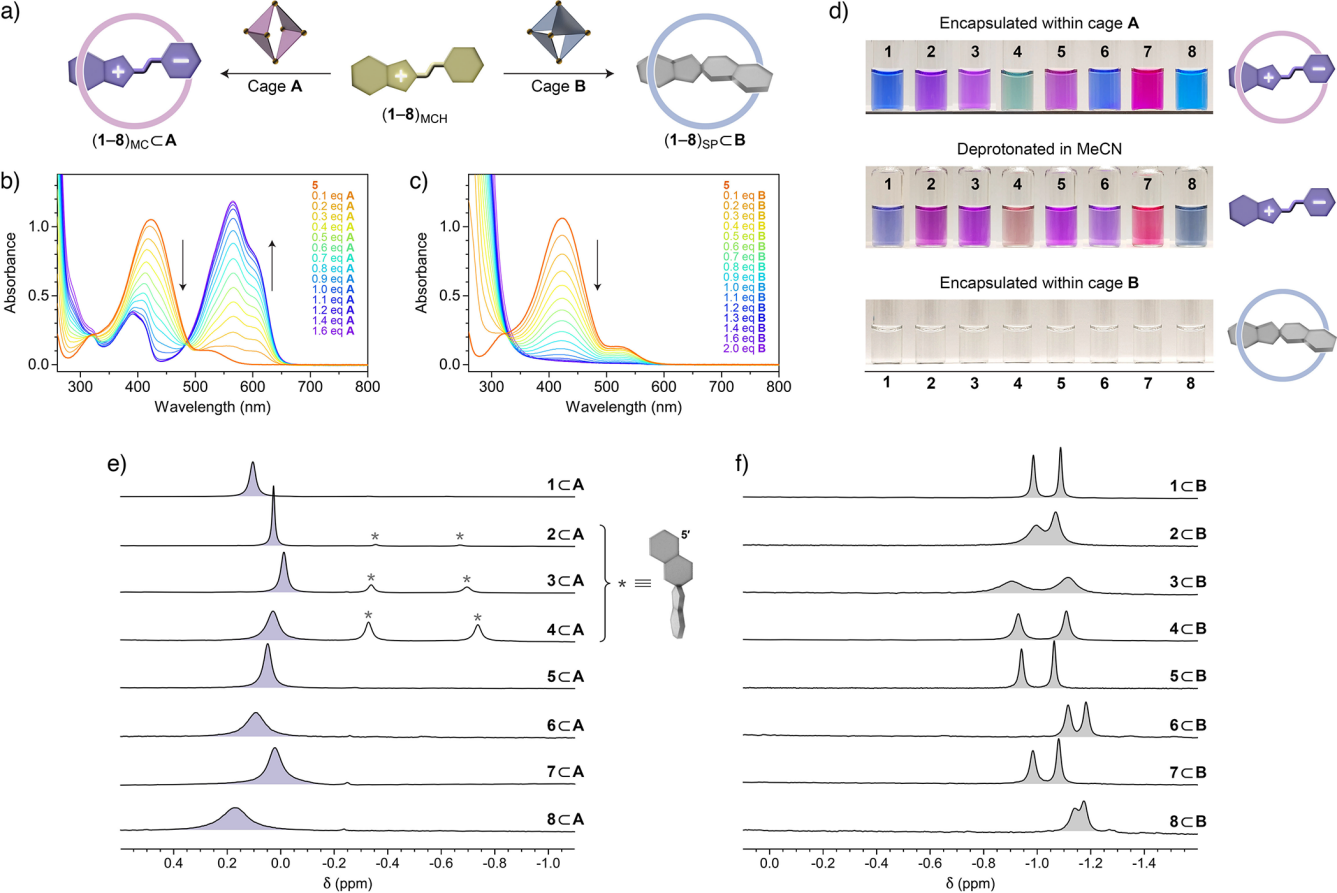
(a) Schematic representation of the MCH^+^ → MC transformation induced by cage **A** (left) and the MCH^+^ → SP transformation induced by cage **B** (right). (b) Changes in the UV/vis absorption spectra of the MCH^+^ form upon the gradual addition of cage **A** (here, illustrated for spiropyran **5**). (c) Changes in the UV/vis absorption spectra of the MCH^+^ form upon the gradual addition of cage **B** (illustrated for spiropyran **5**). (d) Photographs of vials containing spiropyrans **1**–**8** encapsulated within cage **A** (MC isomers; top), dissolved in MeCN in the presence of 1 equiv of KOH (MC isomers; center), and encapsulated within cage **B** (SP isomers; bottom). (e) Partial ^1^H NMR spectra (500 MHz, 298 K, D_2_O) of spiropyrans **1**–**8** encapsulated within cage **A**, focusing on the spiropyrans’ C**H**_3_ protons (the signals denoted with asterisks originate from small amounts of the SP isomer encapsulated within **A**). (f) Partial ^1^H NMR spectra (500 MHz, 298 K, D_2_O) of spiropyrans **1**–**8** encapsulated within cage **B**, focusing on the spiropyrans’ C**H**_3_ protons (the desymmetrization is an inherent feature of the SP isomer and is not related to encapsulation within cage **B**). For full-range spectra, see the Supporting Information, Figures S41–S56.

Recently, we investigated^18^ the complexation of **1** within the cavity of coordination cage **A** (originally reported^46^ by Mukherjee and co-workers; Figure 1b). Similar to the prototypical Fujita cage^47,48^ (**B** in Figure 1b), cage **A** is composed of six *cis*-blocked Pd^2+^ nodes and four triangle-shaped tricoordinate ligands (1,3,5-triimidazolylbenzene instead of 1,3,5-tripyridyltriazine for **B**). Both cages bear a high charge of +12, which results in excellent solubility in water (counterions = NO_3_^–^) and contain a hydrophobic cavity, which enables binding of a wide variety of organic guests. However, despite the structural resemblance of the cage panels, **A** and **B** differ substantially in their architectures: whereas cage **B** adopts a *T*_*d*_ symmetry, cage **A** has a horizontal symmetry plane and a *D*_2*h*_ symmetry (Figure 1b). Interestingly, we found^18^ that the hydrophobic cavity of **A** exhibits a strong affinity to the MC form of **1** (i.e., **1**_MC_; Figure 2a, left), which is otherwise unstable in hydrophobic environments. This observation can be attributed to the combination of (i) shape complementarity between **A**’s cavity and **1**_MC_ and (ii) the polycationic character of cage **A** (which facilitates the deprotonation of MCH).

Here, we hypothesized that the *T*_*d*_ symmetry of cage **B** might translate into its preferential binding of spiropyrans in their closed-ring SP form, which has a quasi-tetrahedral geometry (originating from the central *spiro* carbon atom, denoted with a green asterisk in Figure 1a). This hypothesis is substantiated by the fact that **B**(16,47) and other *T*_*d*_-symmetric cages^13,49−51^ have been shown to strongly bind guests having tetrahedral features. Working with eight differently substituted spiropyrans, we demonstrate that cage **B** induces the conversion of MCH into SP, rendering the SP form photochemically inactive. In contrast, we found that upon binding various spiropyran derivatives, the *D*_2*h*_-symmetric cage **A** retains the open-ring structure of the encapsulated guests (with the liberation of a proton and the transformation of MCH into MC).^52^ Furthermore, owing to its structural flexibility, **A** supports the reversible photochromism of the encapsulated spiropyrans.

## Results and Discussion

### Guest Design

We worked with photoacids **1**–**8** (Figure 1a), which differ in the substitution pattern on the phenol ring. The photoacids were synthesized in a modular fashion by coupling (2,3,3-trimethyl-3*H*-indoliumyl)-1-propanesulfonate with substituted salicylaldehydes (Figure 1a), a strategy previously used to generate libraries of spiropyrans.^53−57^ Compound **1** is the prototypical merocyanine-based photoacid,^28^ and compounds **5** and **7** were previously reported by Liu et al.;^54^ the remaining five photoacids have not been described before (see Supporting Information, Section 2, for synthetic procedures). All eight compounds contain the 3-sulfopropyl chain on the nitrogen atom; we have previously demonstrated^18^ that this substituent reinforces the binding to cage **A** through a pair of hydrogen-bonding interactions between the guest’s SO_3_^–^ group and the host’s acidic imidazole protons (N=C**H**–N). We have also shown that cage **A** has no appreciable affinity to spiropyrans substituted at the 6′ position, owing to the steric clash between substituents at the 6′ position and cage **A**’s axial imidazole groups; therefore, substituents were introduced at the 5′ and 7′ positions (Figure 1a).

### Encapsulation of Spiropyrans within *D*_2*h*_-Symmetric Cage **A** and *T*_*d*_-Symmetric Cage **B**

To study the formation of host–guest complexes, we solubilized spiropyrans **1**–**8** in a minimal amount of methanol, diluted the resulting solutions with water (to 95% v/v H_2_O), and titrated them with concentrated aqueous solutions of both cages. For example, Figure 2b shows the evolution of the UV/vis absorption spectra of spiropyran **5** in the presence of increasing amounts of cage **A**. The initial spectrum features a prominent absorption band centered at ∼420 nm, characteristic of the MCH form. Upon the addition of **A**, this band decreased at the expense of an intense band at ∼565 nm, which can be attributed to the MC isomer (i.e., **5**_MC_). In contrast, a similar experiment using cage **B** as the titrant resulted in bleaching of the solution (Figure 2c), which can be explained by the cage-induced transformation of **5**_MCH_ into **5**_SP_. In both cases, the spectra stopped evolving after >1 equiv of the cage had been added, indicating that all the MCH had been transformed, thus suggesting that the resulting complexes are of 1:1 stoichiometry (i.e., **5**_MC_⊂**A** and **5**_SP_⊂**B**). The other seven spiropyrans behaved analogously when treated with the two cages (Figures S22–S30).

The pronounced changes in the UV/vis spectra accompanying titration allowed us to confirm the 1:1 binding stoichiometry (see the Job’s plots in Figures S22 and S24–S30) and determine the association constants (*K*_assoc_) of all 16 complexes. For example, nonlinear curve fitting of the spectra recorded during the addition of **A** to **1** led to *K*_assoc_ ≈ 1.2 × 10^7^ M^–^^1^ (Figure S22b); the other spiropyrans were also bound strongly, with *K*_assoc_ values in the range (0.24–2.8) × 10^7^ M^–^^1^ (Table 1). Compared with **A**, cage **B** exhibited significantly lower affinity to all eight spiropyrans, with *K*_assoc_ in the range (1.0–7.7) × 10^5^ M^–1^.

**Table 1:** Selected Photophysical and Chemical Properties of the Eight Spiropyrans Investigated Here

compound	λ_max_, MCH^a^	λ_max_, MC⊂**A**^b^	λ_max_, MC,^c^	*K*_assoc, MC⊂**A**_^d^	*K*_assoc, SP⊂**B**_^e^	*k*_SP⊂**A**→MC⊂**A**_^f^	*k*_extract_^g^
**1**	424 nm	590 nm	574 nm	1.2 × 10^7^ M^–1^	1.7 × 10^5^ M^–1^	1.62 h^–1^	1.02 h^–1^
**2**	409 nm	567 nm	554 nm	4.2 × 10^6^ M^–1^	7.7 × 10^5^ M^–1^	1.68 h^–1^	1.02 h^–1^
**3**	412 nm	568 nm	559 nm	3.6 × 10^6^ M^–1^	5.5 × 10^5^ M^–1^	1.02 h^–1^	0.79 h^–1^
**4**	421 nm	590 nm	549 nm	2.8 × 10^7^ M^–1^	6.8 × 10^5^ M^–1^	5.10 h^–1^	11.0 h^–1^
**5**	423 nm	566 nm	550 nm	2.4 × 10^6^ M^–1^	4.6 × 10^5^ M^–1^	16.4 h^–1^	3.12 h^–1^
**6**	413 nm	588 nm	566 nm	3.9 × 10^6^ M^–1^	5.4 × 10^5^ M^–1^	0.96 h^–1^	0.22 h^–1^
**7**	454 nm	552 nm	552 nm	1.2 × 10^7^ M^–1^	1.0 × 10^5^ M^–1^	20.8 h^–1^	31.7 h^–1^
**8**	423 nm	613 nm	588 nm	4.6 × 10^6^ M^–1^	4.1 × 10^5^ M^–1^	0.24 h^–1^	0.18 h^–1^

a Wavelength of maximum absorption (in water with 2% v/v methanol).

b Wavelength of maximum absorption (within **A**; water).

c Wavelength of maximum absorption in the presence of 1 equiv of KOH (in acetonitrile with 2% v/v water).

d Association constant of the complex with cage **A** (water with 2% v/v methanol).

e Association constant of the complex with cage **B** (water with 2% v/v methanol).

f First-order rate constant of the spontaneous back-isomerization for the photochemically generated SP form back to MC within **A**’s cavity.

g (Pseudo)first-order rate constant for the extraction from cage **B** into cage **A**.

Figure 2d shows photographs of the 16 solutions at the end of titration (top and bottom panels). To confirm that the colors of spiropyrans encapsulated within **A** originate from the MC form, we solubilized all eight spiropyrans in MeCN and added 1 equiv of KOH (concentrated solution in water) to deprotonate MCH into MC. The obtained solutions (Figure 2d, middle) had colors analogous to those of **1**–**8** within **A** (although some faded rapidly due to the fast hydrolysis^56,58^ in the basic environment; see, for example, compound **4**). In all cases, the absorption maxima of the confined spiropyrans were slightly red-shifted compared with those solubilized in MeCN; this observation can be explained by the solvatochromic properties of MC, which absorbs at higher wavelengths in more nonpolar environments^59,60^ (here, **A**’s cavity; compared with MeCN). Remarkably, cage **A** stabilized the deprotonated MC even after treating the solutions of MC⊂**A** with small amounts of a strong acid (Supporting Information, Section 4).

The rapid fading of the solutions of unconfined MC is in sharp contrast to the color persistence of the same spiropyrans within cage **A**, which prompted us to study how the encapsulation affects the stability of the MC form more systematically. To this end, we prepared 20 μM solutions of all eight spiropyrans in (i) MeCN (i.e., the MCH form) and (ii) H_2_O in the presence of 1 equiv of **A** (i.e., MC⊂**A**). Then, we induced the formation of MC in MeCN by adding 1 equiv of KOH (the final solvent composition = MeCN with 2 vol% H_2_O); for consistency, we also injected 1 equiv of KOH into (ii). Changes in the UV/vis spectra were monitored immediately following the addition of the base. In all cases, MC’s stability was dramatically improved in the presence of **A** (Figures S33–S40). In an extreme case (spiropyran **7**), MC dissolved in MeCN was quantitatively hydrolyzed after only 30 s; in the presence of the cage, however, no changes in the absorption spectra could be seen after 12 h (Figure S39). This dramatic stabilization can be explained by the encapsulation-induced isolation of MC from the aqueous phase. Such encapsulation-induced enhancement of hydrolytic stability has previously been reported for a range of species otherwise prone to hydrolysis,^61^ ranging from white phosphorus^13^ to cyclic di(lactic acid).^62^ Notably, however, these molecules were encapsulated within small-window cages, similar to **B**. It is worth emphasizing that despite its large window size and, consequently, easily accessible cavity, cage **A** is still very effective in protecting the MC form against hydrolysis in a basic environment. Importantly, encapsulation within **A** can also improve the stability of spiropyrans under irradiation, thus increasing switching reversibility (see below).

The stabilization of the MC vs SP form by cages **A** and **B**, respectively, is further supported by ^1^H NMR spectroscopy. To prepare NMR samples, we added an excess of **1**–**8** to concentrated solutions of cage **A** or **B** in D_2_O, stirred the suspensions overnight, and removed any undissolved solids using a syringe filter. Unencapsulated spiropyrans **1**_MCH_–**8**_MCH_ are poorly soluble in water; in contrast, the solubilities of the inclusion complexes depend on the solubilities of the hosts (which are very high; e.g., ∼95 mmol/L for cage **A**). Therefore, we can assume that the spiropyran signals in the NMR spectra of concentrated solutions prepared in this way originate mainly from the encapsulated guests.

Figure 2e shows the partial ^1^H NMR spectra of all **A** complexes, focusing on the guests’ methyl protons. The singlets at ∼0–0.2 ppm are indicative of the open form of spiropyran; compared with unencapsulated **1**–**8**, these signals are consistently upfield-shifted by ∼1.6–1.8 ppm, suggesting that the methyl groups reside near the center of **A**’s cavity. Binding within cage **B** induced a further shift in the resonance of the guest’s methyl protons (by as much as ∼2.8–2.9 ppm; Figure 2f). Within **B**, these protons appeared as pairs of singlets in a 1:1 ratio, characteristic of the SP form, in which the two methyl groups are not equivalent (Figure 1a). No detectable MC isomer in the NMR spectra of **1**⊂**B** through **8**⊂**B** can be seen (Figure 2f; consistent with the lack of color of the solutions in Figure 2d). Interestingly, however, the spectra of three complexes within cage **A** (for compounds **2**–**4**) feature additional pairs of singlets, indicating the partial existence of these guests in the SP form. Integrating these peaks relative to MC’s singlets at ∼0 ppm reveals that the fraction of the SP isomer corresponds to ∼6% for **2**⊂**A**, ∼24% for **3**⊂**A**, and as much as ∼40% for **4**⊂**A**; that is, it scales with the bulkiness of the substituent at the 5′ position. We note that the presence of a six-membered ring at the *spiro* carbon atom introduces an asymmetry in the SP form (see the projection in Figure 2e). A substituent at the 5′ position can partially compensate for this asymmetry; the larger the substituent, the smaller the asymmetry, which translates into a more efficient filling of the symmetric cavity of cage **A**, shifting the MC ⇌ SP equilibrium to the right. Interestingly, the methyl protons of **2**_SP_, **3**_SP_, and **4**_SP_ are significantly (by ∼0.5 ppm) more upfield-shifted within **B**, indicating that the aromatic cavity of cage **B** shields these protons more efficiently than the cavity of the more porous cage **A**.

### Solid-State Structures of Inclusion Complexes

We also attempted to characterize the inclusion complexes of spiropyrans **1**–**8** within both cages by X-ray crystallography. In general, obtaining single crystals of these host–guest complexes is challenging because the addition of organic solvents (i.e., poor solvents for the cage) reduces the affinity of the cage to the guest (much of which is due to the hydrophobic effect) to the extent that the guest is released, resulting in the crystallization of the empty cage. Therefore, the only suitable method to crystallize the intact inclusion complexes is slow water evaporation, which was previously used to obtain single crystals and to determine the X-ray structure of **1**⊂**A**.^18^ Of the 15 remaining host–guest combinations, two (**2**⊂**A** and **5**⊂**A**) afforded single crystals suitable for X-ray diffraction; evaporating water from the solutions of the other 13 complexes (including all complexes of cage **B**) repeatedly led to noncrystalline films.

**Figure 3:**
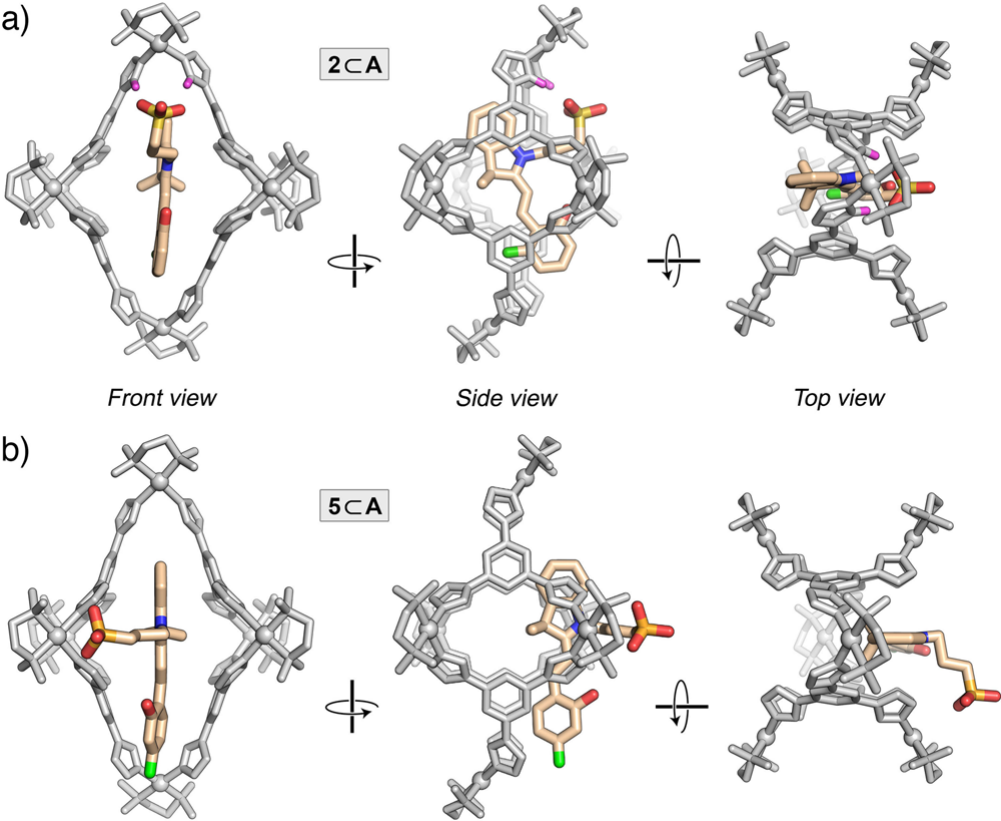
X-ray crystal structures of inclusion complexes **2**⊂**A** (a) and **5**⊂**A** (b), viewed along three different directions. Guest atoms: light-orange = C, blue = N; red = O; green = F; yellow = S. The cage is colored gray, with Pd atoms displayed as balls. Water molecules, counterions, and protons were omitted for clarity (except the acidic imidazole protons interacting with the guest in (a), which are shown in pink).

Inclusion complex **2**⊂**A** crystallized in the *P*1 space group and **5**⊂**A** crystallized in the *Pnnm* space group. In both cases, the host was filled with one guest molecule, in agreement with our NMR and UV/vis spectroscopy results. We found that the solid-state structure of **2**⊂**A** (Figure 3a) is remarkably similar to that of the previously reported^18^**1**⊂**A** (see the overlay of the two structures in Figure S60). Specifically, **2** assumes the open-ring MC form, whose shape is complementary to that of **A**’s cavity. The guest’s quaternary carbon is accommodated in the central part of **A**’s cavity, and its sulfonate group interacts with the cage’s axial imidazoles’ acidic protons (pink in Figure 3a), with an average O_guest_–N_host_ distance of 3.13(9) Å (compared with 3.16(4) Å in **1**_MC_⊂**A**^18^). Note that despite the structural similarity of **1**⊂**A** and **2**⊂**A**, the *K*_assoc_ values derived from the UV/vis titration experiments differed by a factor of ∼3.5.

Interestingly, the guest orientation in the X-ray structure of **5**⊂**A** (Figure 3b) is entirely different from that in **1**⊂**A** and **2**⊂**A**. In the **5**⊂**A** structure—observed consistently in repeated crystallization experiments—**5** occupies only about half of **A**’s cavity, with the remainder filled by several nitrate counterions and water molecules. Moreover, the guest’s propyl chain is not folded so as to form hydrogen bonds with the acidic imidazole protons; instead, it is extended and protrudes from the cavity to maximize the hydrogen-bonding interactions with water molecules, whereas the imidazole protons interact with nitrate counterions. We hypothesize that the unusual conformation of **5**⊂**A** is stabilized in the crystalline state and that the solution structure is similar to the other complexes; indeed, the chemical shifts of the central methylene protons in both **2** and **5** (and other spiropyrans) moved upfield from ∼2.2 ppm to ∼1.2 ppm, suggesting that they are encapsulated (as in Figure 3a). In any case, both X-ray structures unambiguously confirm the 1:1 binding stoichiometry.

### ^19^F GEST NMR Spectroscopy

**Figure 4:**
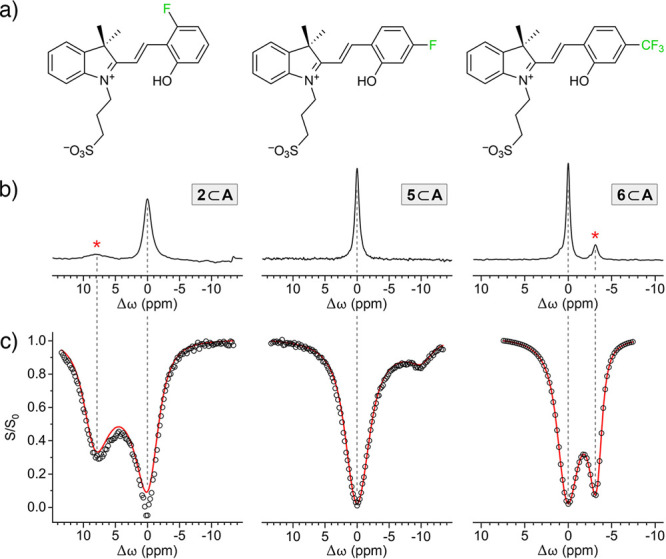
(a) Structural formulas of the three F-containing spiropyrans **2**, **5**, and **6**. (b) ^19^F NMR spectra (D_2_O, 470 MHz, 298 K) of inclusion complexes **2**⊂**A** (left), **5**⊂**A** (center), and **6**⊂**A** (right), with the signals of the encapsulated guests set to 0 ppm (the peaks denoted with asterisks originate from an unbound guest). (c) *z*-spectra of GEST experiments performed at a power (B_1_) of 60 Hz. Markers: experimental data points; red lines: fits to the Bloch–McConnell equations. The concentration of cage **A** as well as spiropyrans **2**, **5**, and **6** in all experiments was 15 mM.

Next, we investigated the inclusion complexes of the fluorinated spiropyrans (**2**, **5**, and **6**) by ^19^F guest exchange saturation transfer (GEST) NMR spectroscopy, which can be used to determine the binding kinetics in host–guest systems.^63−67^ In this method, a mixture of free and encapsulated guests is subjected to a presaturation radiofrequency pulse (B_1_) at a frequency offset of the less dominant species (here, the free guest; the fluorinated spiropyrans exhibit low solubility in water). If the free and bound guests are in fast exchange, the magnetization of the former can be transferred to the NMR signal of the latter, consequently reducing its intensity. By measuring the NMR signal intensity of the encapsulated guest’s F atom as a function of the frequency offset of the applied saturation pulse, we obtained the *z*-spectra (Figure 4c), which effectively amplified the signals originating from the unencapsulated guests. Next, we performed a series of GEST experiments at different B_1_ powers (in the range 5–180 Hz); fitting the resulting *z*-spectra to the Bloch–McConnell equations^68^ (Figures 4 and S61–S63) allowed us to determine the host–guest association rates (*k*_in_) as 4572(±259) s^–1^, 6448(±357) s^–1^, and 748(±60) s^–1^ for **2**⊂**A**, **5**⊂**A**, and **6**⊂**A**, respectively. Unfortunately, evaluating the dissociation rates (*k*_out_) was not possible because of the low aqueous solubility of free **2**, **5**, and **6**. Nevertheless, the high *k*_in_ values indicate the fast binding of guests (here, fluorinated spiropyrans) within cage **A**, as expected from the cage’s porous structure.^69^ The fast guest exchange kinetics are also manifested by peak broadening in the ^19^F spectra (Figure 4b). In contrast, the signals in the ^19^F NMR spectra of the three spiropyrans within cage **B** were sharp (Figures S57–S59), which implies slow (on the NMR time scale) exchange^70^ (indeed, we did not observe any GEST effect with **2**⊂**B**, **5**⊂**B**, and **6**⊂**B**).

Similar differences in signal broadness were evident from the ^1^H NMR spectra of the **A** vs **B** complexes; we found that the spectra of the **A** complexes generally featured signals broader than the spectra of the **B** complexes (Figures S41–S48 and S49–S56, respectively). Compared with **B**, cage **A** has large windows through which guests can rapidly enter and leave the cage (the binding and unbinding kinetics are likely accelerated further by **A**’s high structural flexibility^18,71^). In contrast, the four windows of cage **B** are too small to allow the passage of guests **1**–**8**; therefore, guest uptake and release must proceed via a different mechanism:^72−75^ one involving a temporary dissociation of a Pd–N bond, which explains the slower kinetics.

### Light-Responsiveness of Spiropyrans within Cages **A** and **B**

**Figure 5:**
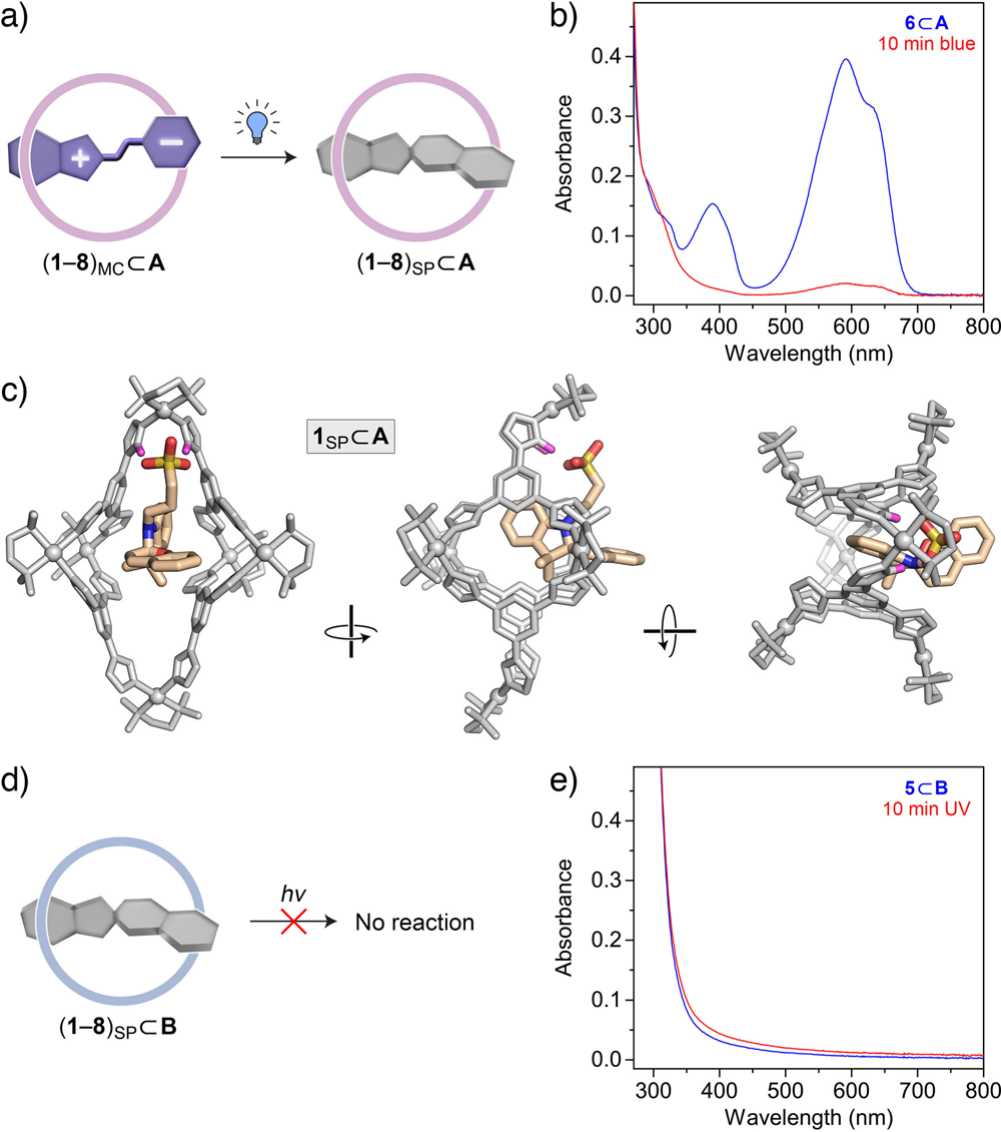
(a) Schematic representation of the light-induced transformation of MC into SP within the cavity of cage **A**. (b) UV/vis absorption spectra of an encapsulated MC form of spiropyran (here, **6**) before (blue) and after (red) exposure to blue light (460 nm) for 10 min. (c) Energy-optimized structure of the metastable **1**_SP_⊂**A** inclusion complex viewed from three different directions. Guest atoms: light-orange = C, blue = N; red = O; green = F; yellow = S; the cage is colored gray, with Pd atoms displayed as balls; counterions and protons were omitted for clarity (except the acidic imidazole protons interacting with the guest, which are shown in pink). (d) Spiropyrans **1**–**8** encapsulated within cage **B** are not photoswitchable. (e) The UV/vis absorption spectra of an encapsulated SP form of spiropyran (here, illustrated for **5**) before (blue) and after (red) exposure to UV light for 10 min.

Upon exposure to blue light,^76^ the blue solutions of the (**1**–**8**)_MC_⊂**A** complexes faded (e.g., Figure 5b), indicating photoisomerization to the SP form (Figure 5a). The unsubstituted spiropyran **1**⊂**A** turned colorless within 20 s; other, bulkier guests required longer irradiation times (200–400 s) for the reaction to complete (except **7**⊂**A**, which reacted very slowly, with less than 50% conversion after 1 h of irradiation). The reaction could also be followed by NMR, as illustrated in Figures S80 and S81 for **4**⊂**A**. Despite significant efforts, we did not succeed to crystallize any of the (**1**–**8**)_SP_⊂**A** complexes (owing to the spontaneous back-isomerization; see below). Figure 5c shows a DFT-optimized model of the **1**_SP_⊂**A** complex, which retains the hydrogen bonds between **1**’s sulfonate group and **A**’s acidic imidazole protons. Compared with the starting structure used in the calculations (i.e., the X-ray structure of **1**_MC_⊂**A**, in which the MC isomer was replaced by SP), the cage within **1**_SP_⊂**A** is significantly distorted. We have previously demonstrated that host **A** is highly flexible^18,71^ and argued that this flexibility is vital to accommodate two structurally different isomers of a photoresponsive guest, thus providing a suitable environment for efficient photoswitching of the bound guest.^18,77,78^ Compared with **A**, the *T*_*d*_-symmetric cage **B** is highly rigid and unable to adapt to efficiently stabilize the open-ring isomer of spiropyrans. Indeed, irradiation of the (**1**–**8**)_SP_⊂**B** complexes did not induce any appreciable changes in their UV/vis spectra (as illustrated for **5**_SP_⊂**B** in Figure 5d,e), irrespective of the irradiation period and the wavelength of incident light (including 365 nm, which is typically used to convert SP into MC). We note, however, that the ring-opening reaction can also be hampered by the high UV absorption cross-section^14^ of cage **B**.

### Thermal Back-Isomerization of Spiropyrans within Cage **A**

**Figure 6:**
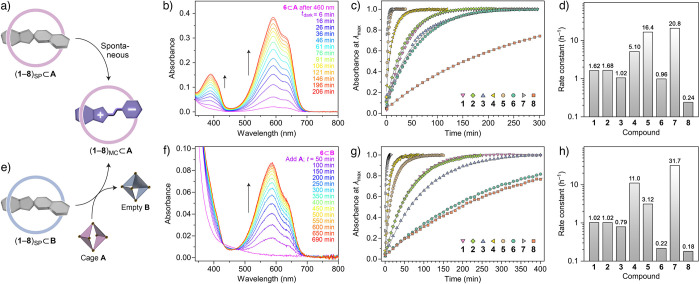
(a) Schematic representation of the thermal relaxation of the encapsulated SP form of spiropyran into the MC form. (b) UV/vis absorption spectra accompanying the back-isomerization of the encapsulated SP form into the MC form (here, illustrated for spiropyran **6**). (c) Following the kinetics of the back-isomerization of spiropyrans **1**–**8** encapsulated within cage **A** by monitoring the absorbance at the wavelength of maximum absorption of the respective MC form (see Table 1). Markers: experimental data points; gray lines: fits to a first-order rate equation. (d) Rate constants for the spontaneous SP → MC back-isomerization within **A** obtained by fitting MC’s recovery to the first-order reaction model. (e) Schematic representation of the extraction of spiropyran encapsulated within cage **B** by cage **A**, accompanied by isomerization from SP into MC. (f) UV/vis absorption spectra accompanying the extraction of spiropyran (here, **6**) from cage **B** (5 equiv) into cage **A** (5 equiv). (g) Following the kinetics of the ring-opening of spiropyrans **1**–**8** induced by cage **A**. Markers: experimental data points; gray lines: fits to a first-order rate equation. (h) Rate constants for the extraction of spiropyrans from cage **B** into cage **A**, obtained by fitting the MC absorption vs time profiles to the first-order reaction model.

The photochemically generated inclusion complexes (**1**–**8**)_SP_⊂**A** are metastable, and they spontaneously revert to the initial species (**1**–**8**)_MC_⊂**A** in the dark (Figure 6a). For example, the series of spectra in Figure 6b follow the reaction **6**_SP_⊂**A** → **6**_MC_⊂**A**; by plotting the absorbance at 588 nm (λ_max_) over time, we conclude that the reaction obeys first-order kinetics, with a rate constant of *k*_SP⊂**A**→MC⊂**A**_ = 0.96 h^–1^. Next, we set out to study the kinetics of the back-isomerization reaction for the other spiropyrans. Figure 6c plots the recovery of all eight MC⊂**A** complexes under the same conditions; similar to **6**_SP_⊂**A**, the other SP complexes reacted with first-rate constants, which are listed in Figure 6d. These results show that the reaction rates vary significantly, depending on the spiropyran’s substitution pattern, with the largest difference (between **7**⊂**A** and **8**⊂**A**) approaching 2 orders of magnitude.

### Reversible Photochromism of Encapsulated Spiropyrans and “Self-Erasing” Images with Tunable Lifetimes

**Figure 7:**
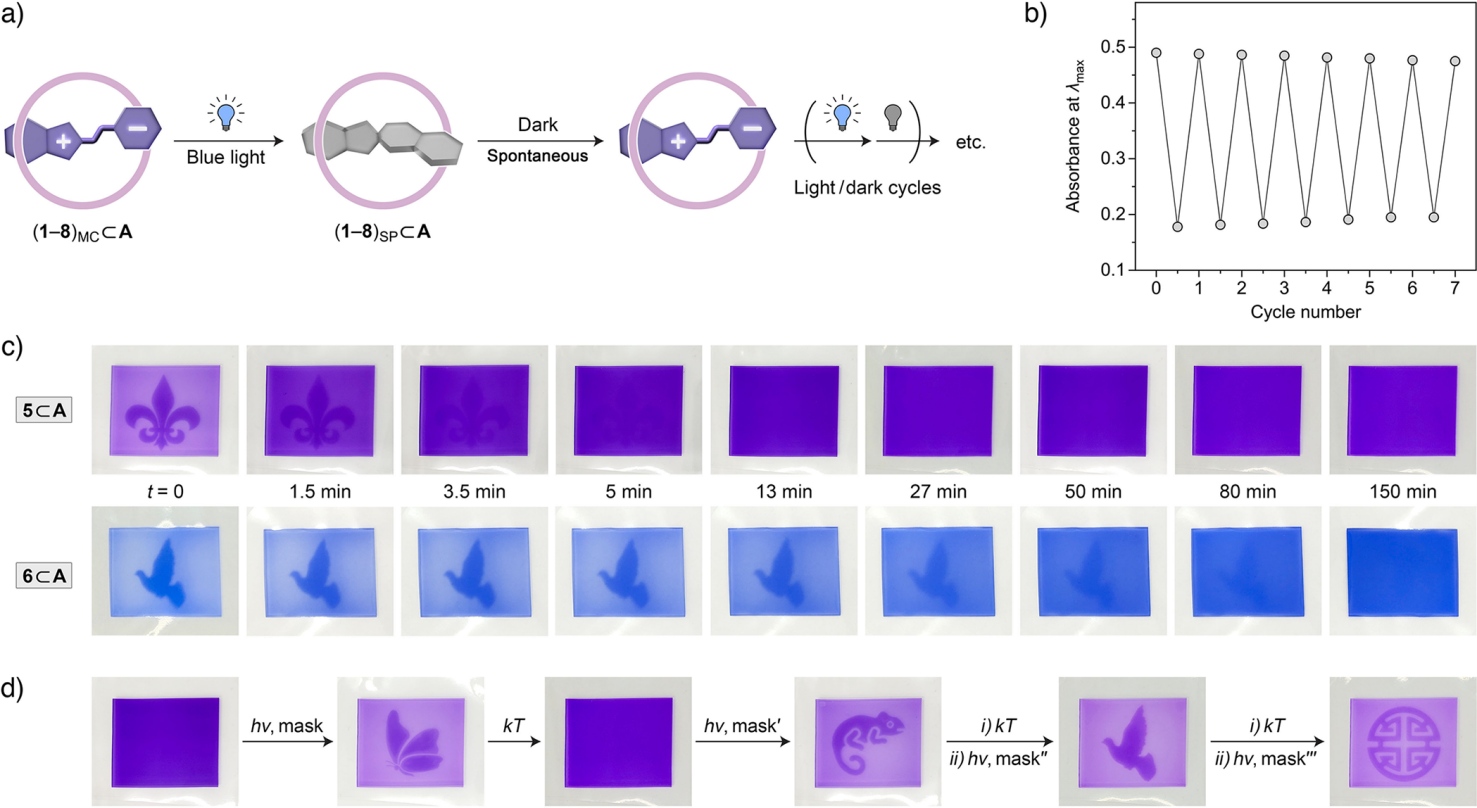
(a) Schematic representation of the reversible, light-induced isomerization of spiropyran within cage **A**. (b) Reversible changes in absorbance at λ_max_ of the encapsulated spiropyran’s MC form (here, **5**; λ_max_ = 566 nm) upon alternating cycles of blue light irradiation (λ = 460 nm; 30 s) and incubation in the dark (90 min). (c) Photographs of agarose gels (45 × 35 × 1 mm) soaked with 2 mM aqueous solutions of **5**⊂**A** (top) and **6**⊂**A** (bottom); the photographs were taken at different times after a 1 min exposure to blue light (460 nm) through a mask. (d) A series of images created consecutively in the same piece of agarose gel (soaked with a 2 mM aqueous solutions of **5**⊂**A**) by irradiating through different masks (*h*ν = 460 nm light for 1 min; *kT* = 2 h in the dark).

Once the photochemically generated **6**_SP_⊂**A** (Figure 5b) relaxed to **6**_MC_⊂**A** (Figure 6b), the cycle could be repeated many times (Figure 7a,b). Similarly, guests **1**, **2**, **4**, and **5** exhibited excellent switching reversibility (Supporting Information, Section 9.2). Notably, the encapsulation within **A** considerably improved the reversibility of switching for spiropyrans **2** and **6**, which, under the same irradiation conditions, experienced significant fatigue during repeated photoisomerization (e.g., compare Figure S65 with S73). However, spiropyrans **3**, **7**, and **8** degraded relatively quickly both inside and outside the cage, either because of the long irradiation times required to generate the SP isomer or because of a very slow back-isomerization (during which a substantial fraction of the guest decomposed) (Supporting Information, Section 9).

We have previously demonstrated that agarose gels soaked with **1**_MC_⊂**A** can serve as “reusable paper”, in which “self-erasing” messages can be created multiple times;^18^ however, the ability to tune the lifetimes of these messages was lacking. Of the five spiropyrans that exhibited good switching reversibility, **5** and **6** have the fastest and the slowest back-isomerization kinetics, respectively (differing by a factor of ∼17). To demonstrate the applicability of the inclusion complexes of substituted spiropyrans as the key components of reusable information storage media, we prepared thin (1 mm) pieces of agarose gels and soaked them with 2 mM aqueous solutions of **5**_MC_⊂**A** and **6**_MC_⊂**A**. The two gels exhibited intense purple and blue colors, respectively. Next, we exposed both gels to 460 nm light through a mask for 1 min, thus inducing bleaching in the regions exposed to light (Figure 7c, *t* = 0). Then, we followed the recovery of the deep color typical of the MC⊂**A** isomers; the series of photographs in the top panel of Figure 7c shows that for **5** the back-reaction took 3.5–5 min to complete. As expected, the back-isomerization **6**_SP_⊂**A** to **6**_MC_⊂**A** required more time; we found that the pattern disappeared after 50–80 min (Figure 7c, bottom panel).

The high reversibility of the system allowed us to create multiple images in the same gel. Once the initially generated image disappeared, the same gel could be exposed to blue light through a different mask, thus assuming a new pattern. For example, the chameleon image fabricated in the gel soaked with **5**⊂**A** (Figure 7d) similarly persisted for up to five minutes, after which subsequent images could be created consecutively.

### Extraction of Spiropyrans Bound within Cage **B** Using Cage **A**

Finally, having found that cage **A** binds spiropyrans stronger than cage **B** (by factors ranging from ∼2.6 for **5** to ∼120 for **7**; Table 1), we hypothesized that **A** should be able to “extract” the guests from the (**1**–**8**)_SP_⊂**B** complexes (Figure 6e). Here, we note that both (i) the SP → MC back-isomerization within **A** and (ii) the extraction of MC from **B** into **A** leads to the same species (MC⊂**A**; Figure 6a vs e); therefore, we found it intriguing to compare the rates of the two processes.

The transfer of spiropyrans from cage **B** into cage **A** can be conveniently followed by UV/vis absorption spectroscopy, since it is accompanied by the transformation of the colorless SP into the intensely colored MC isomer (similar to the SP → MC back-isomerization within **A**). It is important to point out that the binding of **1**_SP_–**8**_SP_ within **B** is relatively weak (Table 1); consequently, the UV/vis spectra (typically recorded at micromolar concentrations) of 1:1 mixtures of spiropyrans **1**_MCH_–**8**_MCH_ and cage **B** display a low-intensity absorption band at ∼420 nm due to residual unbound MCH (in equilibrium with the encapsulated SP). To ensure a quantitative encapsulation of spiropyrans, we treated them with 5 equiv of **B**; next, we injected 5 equiv of the competing binder **A** and monitored the evolution of the UV/vis spectra, as illustrated in Figure 6f for spiropyran **6**. Similar to the thermal relaxation within **A**, the rate of extraction of **6** by **A** could be fitted to (pseudo)first-order kinetics (the same was true for the other seven spiropyrans; see Figure 6g,h). For the reaction **6**_SP_⊂**B** + **A** → **6**_MC_⊂**A** + **B**, we found *k*_extract_ = 0.22 h^–1^; that is, it proceeded ∼4.5 times slower than the back-isomerization of **6** within cage **A**. Similarly, the thermal isomerization was faster than the extraction-induced ring opening for **1**–**3**, **5**, and **8** (by factors ranging from ∼1.3 for **8** to ∼5.3 for **5**). In two cases (methoxylated spiropyrans **4** and **7**), however, we found *k*_extract_ to be higher than *k*_SP⊂**A**→MC⊂**A**_. This surprising finding led us to hypothesize that prior to encapsulation within **A**, spiropyrans **4** and **7** undergo ring-opening in solution (i.e., SP → MCH). To this end, we studied the kinetics of thermal relaxation of all eight spiropyrans dissolved in water and found that the reaction rate depended strongly on spiropyran’s identity (Figures S64–S71), with the fastest spiropyran (**7**) reacting over 4 orders of magnitude faster than the slowest one (**3**). Surprisingly, the rate of extraction of **4** (a process that entails ring-opening) was found to proceed faster than its ring-opening both in solution (**4**_SP_ → **4**_MCH_) and under confinement (**4**_SP_⊂**A** → **4**_MC_⊂**A**). This unexpected result (confirmed in repeated experiments) highlights the complexity of the process and indicates that additional scenarios should be considered, such as concerted unbinding/ring-opening, whereby the release of spiropyran from cage **B** facilitates the cleavage of the dihydropyran ring.

## Conclusions

In sum, we investigated the encapsulation of eight spiropyran derivatives within the cavities of the *D*_2*h*_-symmetric cage **A** and the *T*_*d*_-symmetric cage **B**. The cavities of the two cages are similarly sized but have significantly different shapes. The elongated, quasi-2D cavity of cage **A** and the tetrahedral cavity of **B** have shapes complementary to the two isomers of spiropyrans: the open-ring MC and the closed-ring SP, respectively. Indeed, we found that cage **A** stabilized the MC form and cage **B** stabilized the SP form to the extent that they could shift the MC ⇌ SP equilibrium into either direction nearly quantitatively for most of the spiropyrans studied here. In addition to their different symmetries, the two cages differ in terms of flexibility; cage **A** is highly flexible, whereas **B** is structurally rigid. The rigid nature and high UV absorption cross-section of cage **B** rendered the encapsulated SP isomers photochemically inert; they could not be converted to the open-ring MC isomer even under irradiation with UV light. In contrast, cage **A** could adjust its shape in order to stabilize both SP and MC; hence, it supported the reversible photochromism of spiropyrans. All eight spiropyran derivatives could be transformed from the MC isomer into the metastable SP form upon exposure to blue light; in the dark, the colorless SP isomer spontaneously reverted to the intensely colored MC, with kinetics strongly dependent on the spiropyran’s substitution pattern. Among the five spiropyrans that exhibited highly reversible photoswitching within the cavity of **A**, the 7′-F-substituted spiropyran back-isomerized the fastest, whereas 7′-CF_3_-spiropyran was the slowest, with a ∼17-fold difference in the reaction rate of the two complexes. These results form the basis of tuning the lifetime of transient images in the thin films of hydrogels doped with the photoswitchable complexes. Overall, our results indicate that the symmetry of coordination cages profoundly affects the behavior of the switchable molecules confined within their cavities. Future studies will explore other spiropyran derivatives; for example, we hypothesize that varying the length of the alkyl chain (terminated with the sulfonate group, which binds strongly to the cage through a pair of hydrogen bonds) can have a substantial effect on the behavior of the binding strength and chemical reactivity of encapsulated spiropyrans. We are also planning to extend our studies on the relationship between cage symmetry and the photochromism of the confined molecules to other classes of photoswitchable compounds, such as diarylethenes^79^ and spirooxazines.^80,81^

## Supporting Information Available

General experimental considerations, synthetic procedures, characterization by UV/vis and NMR spectroscopy, single-crystal X-ray diffraction, and DFT calculations (PDF)
DFT-optimized model (PDB)

## Supplementary Material

ja2c08901_si_001.pdf

ja2c08901_si_002.pdb
