# Supplementary material for: Altering the Properties of Spiropyran Switches Using Coordination Cages with Different Symmetries
Source: J Am Chem Soc. 2022 Nov 15;144(46):21244–54. doi: 10.1021/jacs.2c08901 (PMC9706567; doi:10.1021/jacs.2c08901)
Supplement: Supplementary file 1 — ja2c08901_si_001.pdf [file ja2c08901_si_001.pdf]

Supporting Information for:

Altering the Properties of Spiropyran Switches using Coordination Cages with Different Symmetries

Jinhua Wang,[1] Liat Avram,[2] Yael Diskin-Posner,[2] Michał J. Białek,[1,3] Wojciech Stawski,[1] Moran Feller,[1] Rafal Klajn[1]*

[1]Department of Organic Chemistry, Weizmann Institute of Science, Rehovot 76100, Israel
[2]Department of Chemical Research Support, Weizmann Institute of Science, Rehovot 76100, Israel
[3]Department of Chemistry, University of Wrocław, 14 F. Joliot-Curie St., 50383 Wrocław, Poland

*e-mail: rafal.klajn@weizmann.ac.il

Table of contents:

1. General remarks 2
2. Synthesis of cages and spiropyran guests 2
3. Encapsulation of spiropyrans studied by UV/vis absorption spectroscopy 18
4. Stabilization of the MC form under acidic conditions 26
5. Cage **A** protects the MC form of spiropyrans against hydrolysis 28
6. NMR spectra of encapsulated spiropyrans 32
7. X-ray data collection and structure refinement 42
8. Guest exchange saturation transfer (GEST) experiments 43
9. Photoresponsive properties of free and encapsulated spiropyrans 47
    9.1. Photoswitching of free spiropyrans 47
    9.2. Photoswitching of spiropyrans within cage **A** 50
    9.3. Attempts to photoswitch spiropyrans within cage **B** 57
10. DFT calculations 57
11. Extraction of guests from cage **B** to cage **A** 58
12. Preparation of photoresponsive agarose gels 60
13. Supporting references 61

1. General remarks

All commercially available chemicals were used as received unless otherwise specified. NMR spectra were recorded on a Bruker Avance III 400 MHz spectrometer or a Bruker Avance III HD 500 MHz spectrometer. Chemical shifts (δ) are given in ppm relative to residual protio solvent resonances (4.79 ppm for D_2O and 2.50 ppm for DMSO-d_6). Electrospray ionization mass spectrometry (ESI-MS) experiments were carried out on a Bruker Daltonics Esquire 300 Plus ESI mass spectrometer using spectroscopic-grade methanol. UV/vis absorption spectra were recorded on a Shimadzu UV-2700 spectrophotometer or an Agilent Cary 60 spectrophotometer. For photoirradiation experiments, we used a Prizmatix mic-LED 420 nm and 460 nm light-emitting diodes (LEDs) as blue light sources and a Prizmatix Mic-LED 365 nm LED as the UV light source. To follow the photoisomerization reactions using NMR spectroscopy under in-situ irradiation, the LEDs were equipped with a high-numerical-aperture polymer optical fiber (diameter = 1 mm, length = 5 m).

2. Synthesis of cages and spiropyran guests

Cage A: The cage[1] was synthesized according to a previous report.[2] Specifically, 1,3,5-tri(imidazol-1-yl)benzene (553 mg, 2.0 mmol) was suspended in the aqueous solution (8 mL) of $[Pd(NO_3)_2(tmeda)]$ (1.04 g, 3.0 mmol) and the mixture was stirred at room temperature overnight until the white solid has completely dissolved. After filtration through a membrane, the clear yellow solution was concentrated to approximately 2 mL. Pure cage **A** was obtained as needle crystals by slow vapor diffusion of acetone at room temperature (yield = 92%). ^{1}H NMR (500 MHz, D_2O, 298 K): δ (ppm) = 9.10 (s, 8 H), 8.82 (s, 4 H), 7.74 (s, 4 H), 7.71 (s, 4 H), 7.69 (s, 8 H), 7.64 (s, 8 H), 7.54 (s, 12 H), 3.11 (s, 24 H), 2.77–2.70 (m, 72 H).

Cage B: The cage[3] was synthesized analogously to the procedure described above. Specifically, 2,4,6-tri(pyridin-4-yl)-1,3,5-triazine (625 mg, 2.0 mmol) was suspended in the aqueous solution (8 mL) of $[Pd(NO_3)_2(tmeda)]$ (1.04 g, 3.0 mmol) and the mixture was stirred at room temperature overnight until the white solid has completely dissolved. After filtration through a membrane, the clear yellow solution was concentrated *in vacuo* to dryness, resulting in a light-yellow solid (yield = 95%). ^{1}H NMR (500 MHz, D_2O, 298 K): δ (ppm) = 9.33 (d, J = 6.5 Hz, 24 H), 8.81 (d, J = 6.5 Hz, 24 H), 3.21 (s, 24 H), 2.81 (s, 72 H).

General procedure for the synthesis of spiropyrans: Spiropyrans **1**–**8** were prepared by condensing (2,3,3-trimethylindoliumyl)propane-1-sulfonate **S0** with the corresponding aldehyde (**S1** through **S8**), according to the generic scheme[4] outlined in Figure S1. Aldehydes **S1** and **S3** were obtained from commercial resources and used as received. Aldehydes **S2** and **S4**–**S8** were synthesized according to previously described procedures;[5–8] their ^{1}H NMR spectra matched those reported previously.[5–8] The respective aldehyde and **S0** (1:1 molar ratio) were dissolved in absolute ethanol and the solution was heated at reflux overnight under nitrogen. After cooling down to room temperature, yellow or orange solids precipitated from the solution. The solids were collected by filtration, washed several times with cold ethanol, and dried under high vacuum. The yield ranged from 50% to 75%, depending on the aldehyde (see below).

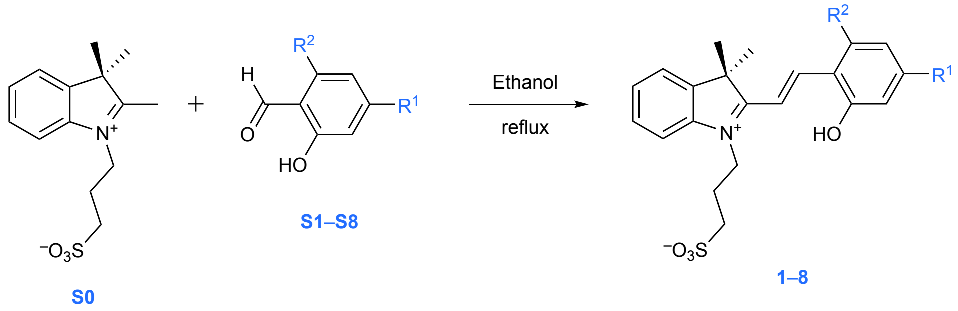

**1**:	R^1 = H	R^2 = H
**2**:	R^1 = H	R^2 = F
**3**:	R^1 = H	R^2 = Cl
**4**:	R^1 = H	R^2 = OMe
**5**:	R^1 = F	R^2 = H
**6**:	R^1 = CF_3	R^2 = H
**7**:	R^1 = OMe	R^2 = H
**8**:	R^1 = COOMe	R^2 = H

Scheme S1. Synthetic scheme for differently substituted spiropyrans.

Spiropyran **1**: Synthesized as described before.[4]

Spiropyran **2**: Prepared according to the general procedure (see above), starting with 258 mg (0.92 mmol) of **S0** and 134 mg (0.95 mmol) of **S2**. Yield = 240 mg (65%). ^{1}H NMR (500 MHz, DMSO-d_6, 298 K): δ (ppm) = 11.82 (s, 1 H), 8.34 (d, J = 16.6 Hz, 1 H), 8.10 (d, J = 7.0 Hz, 1 H), 7.89 (d, J = 16.6 Hz, 1 H), 7.88 (d, J = 7.0 Hz, 1 H), 7.65 (m, 2 H), 7.49 (dd, J = 16.0, 7.0 Hz, 1 H), 6.92 (d, J = 8.3 Hz, 1 H), 6.88 (t, J = 9.6 Hz, 1 H), 4.71 (t, J = 7.6 Hz, 2 H), 2.64 (t, J = 6.8 Hz, 2 H), 2.20 (m, 2 H), 1.79 (s, 6 H); ^{13}C NMR (125 MHz, DMSO-d_6, 298 K): δ (ppm) = 182.2, 163.3, 161.2, 160.3, 160.2, 143.6, 142.6, 141.0, 135.7, 135.6, 129.5, 129.2, 123.0, 115.5, 115.2, 115.1, 112.8, 110.5, 110.4, 106.4, 106.2, 52.1, 47.8, 46.3, 26.2, 24.5; ^{19}F NMR (470 MHz, DMSO-d_6, 298 K): δ = –111.3 ppm; ESI-HRMS calcd. for $C_{21}H_{21}FNO_4S$ $[M - H]^-$: 402.1175, found: 402.1183.

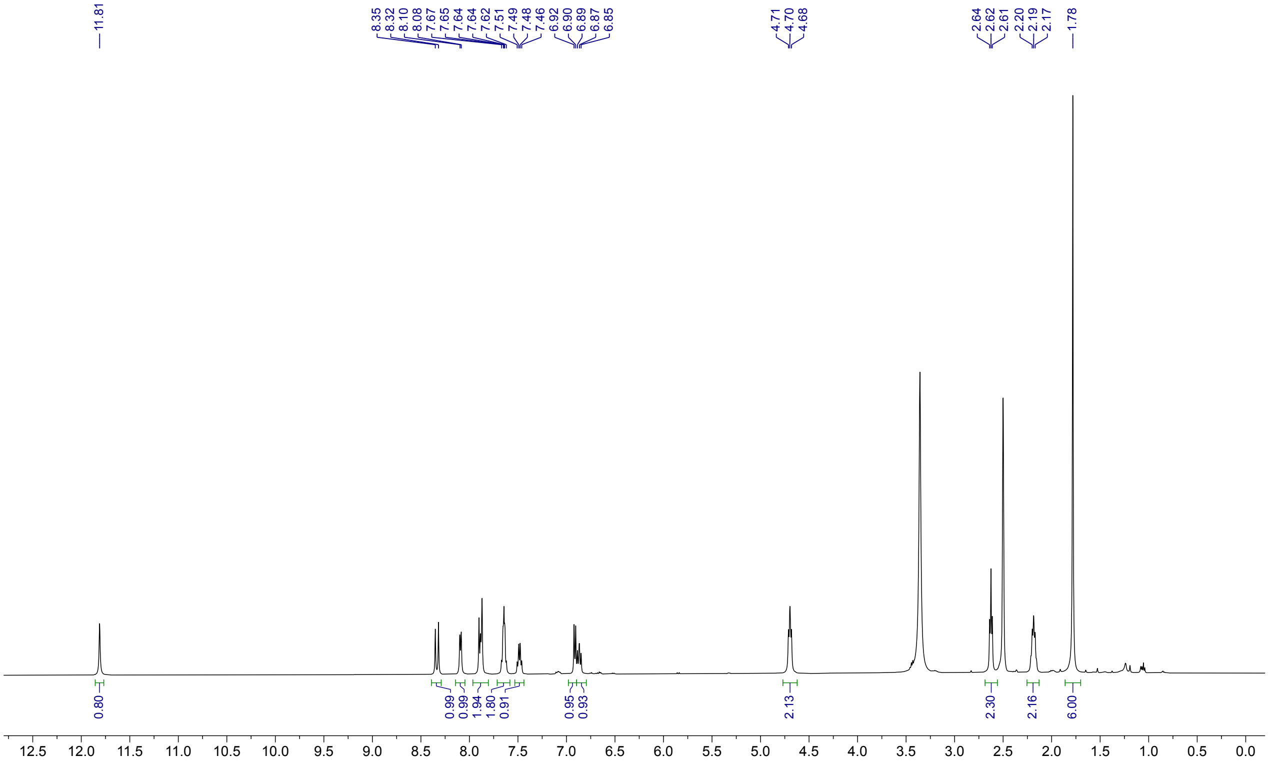

Figure S1. ^{1}H NMR spectrum of **2** (500 MHz, DMSO-d_6, 298 K).

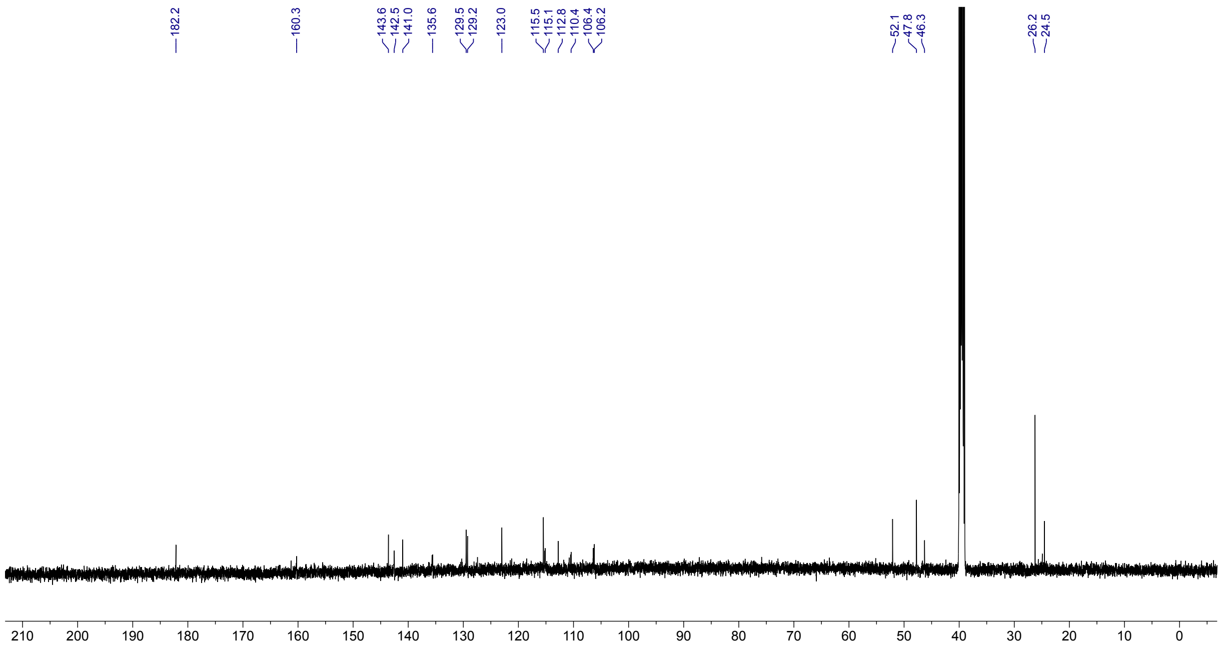

Figure S2. ^{13}C NMR spectrum of **2** (125 MHz, DMSO-d_6, 298 K).

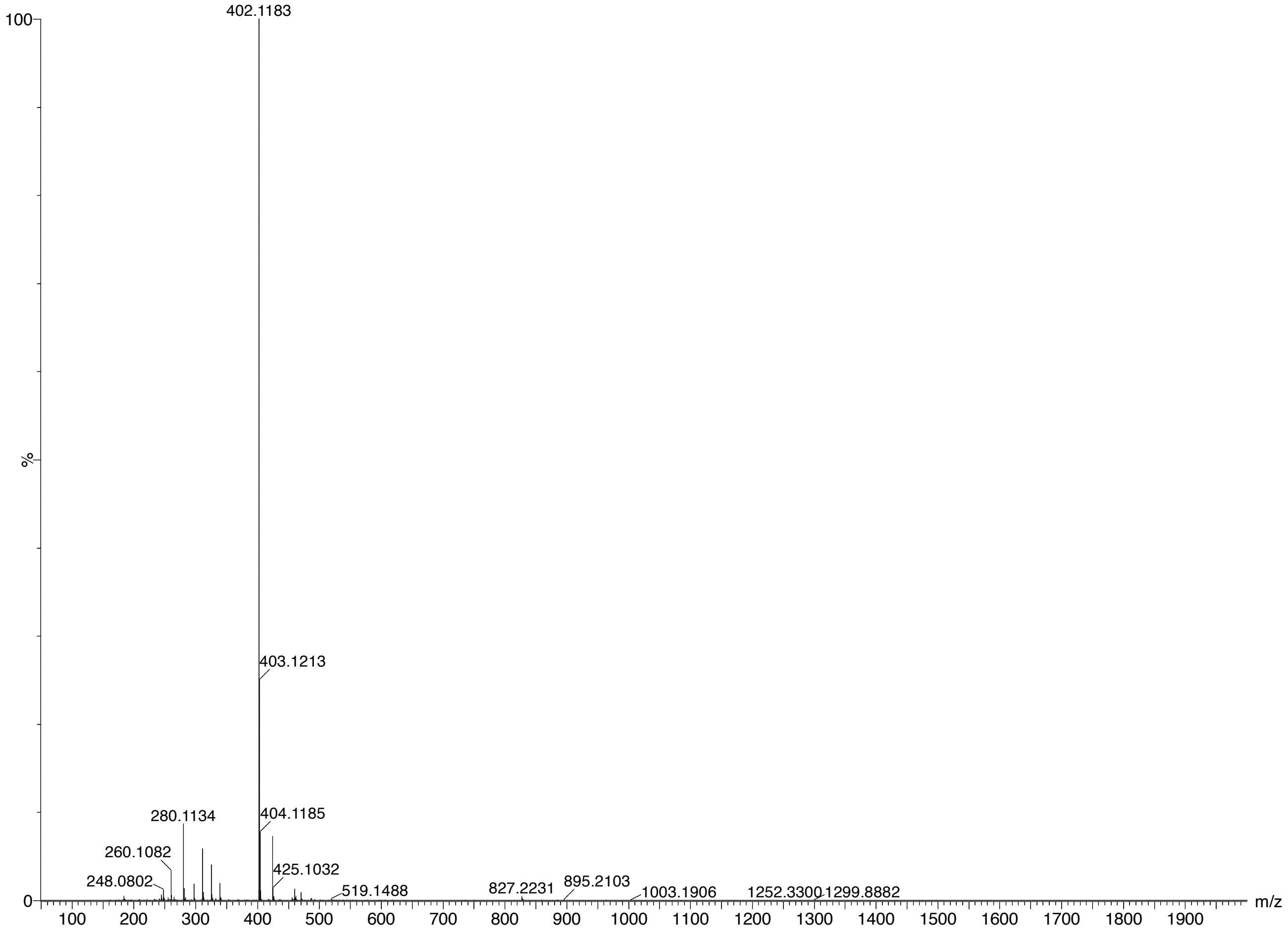

Figure S3. A high-resolution ESI-TOF mass spectrum of **2**.

Spiropyran **3**: Prepared according to the general procedure (see above), starting with 205 mg (0.73 mmol) of **S0** and 115 mg (0.73 mmol) of **S3**. Yield = 220 mg (72%). ^{1}H NMR (400 MHz, DMSO-d_6, 298 K): δ (ppm) = 12.03 (s, 1 H), 8.14 (d, J = 16.2 Hz, 1 H), 8.10 (d, J = 7.2 Hz, 1 H), 7.90 (m, 1 H), 7.66 (m, 2 H), 7.44 (t, J = 8.1 Hz, 1 H), 7.15 (d, J = 7.2 Hz, 1 H), 7.07 (d, J = 8.2 Hz, 1 H), 4.69 (t, J = 7.6 Hz, 2 H), 2.64 (t, J = 7.2 Hz, 2 H), 2.20 (m, 2 H), 1.81 (s, 6 H); ^{13}C NMR (100 MHz, DMSO-d_6, 298 K): δ (ppm) = 182.4, 161.0, 146.4, 143.6, 141.0, 136.5, 134.8, 129.5, 129.2, 123.0, 120.9, 118.5, 116.5, 116.1, 115.5, 52.0, 47.8, 46.4, 26.3, 24.5; ESI-HRMS calcd. for $C_{21}H_{21}ClNO_4S$ [M – H]$^{-}$: 418.0880, found: 418.0884.

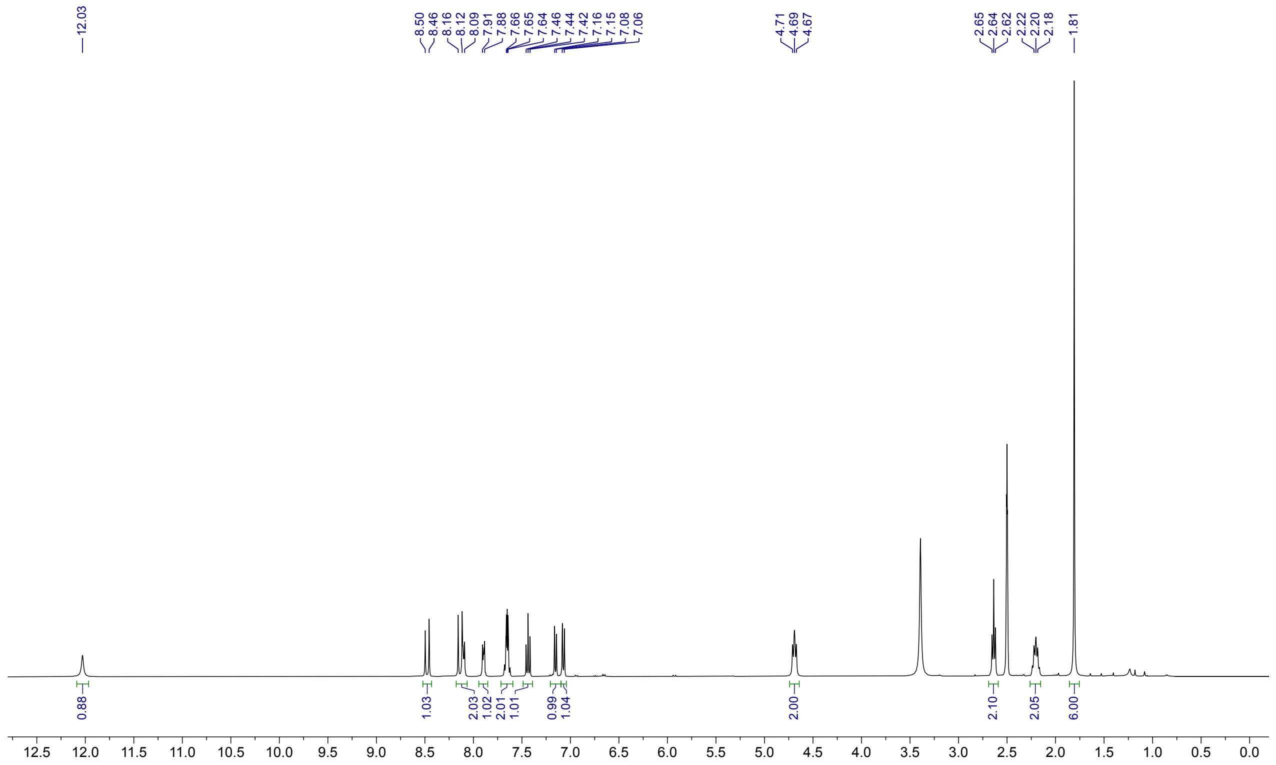

Figure S4. ^{1}H NMR spectrum of **3** (400 MHz, DMSO-d_6, 298 K).

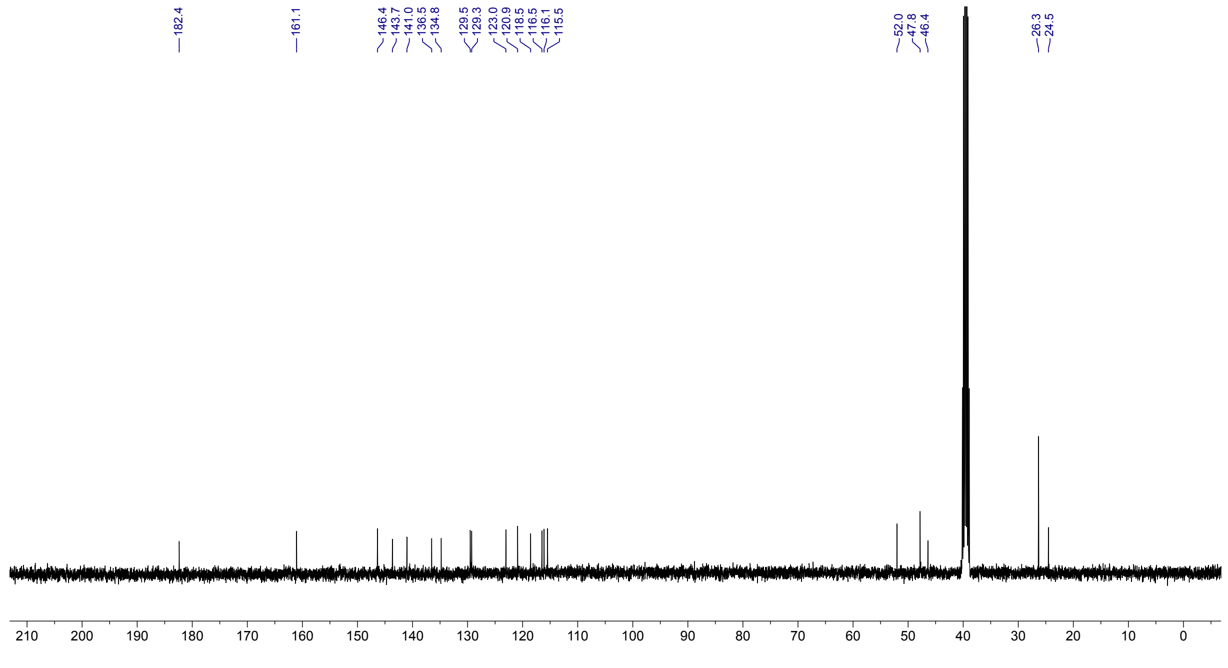

Figure S5. ^{13}C NMR spectrum of **3** (100 MHz, DMSO-d_6, 298 K).

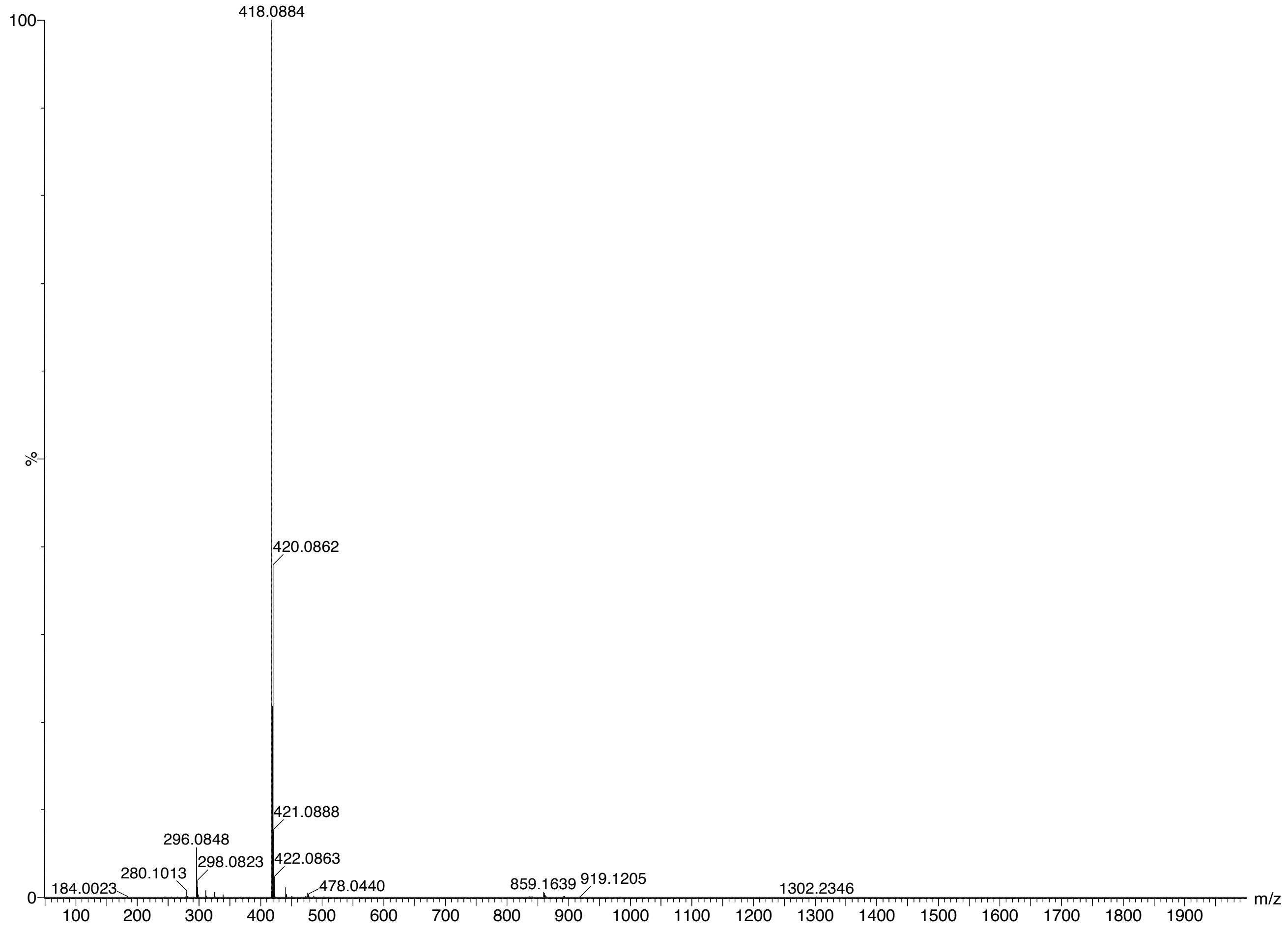

Figure S6. A high-resolution ESI-TOF mass spectrum of **3**.

Spiropyran **4**: Prepared according to the general procedure (see above), starting with 259 mg (0.92 mmol) of **S0** and 142 mg (0.93 mmol) of **S4**. Yield = 260 mg (68%). ^{1}H NMR (500 MHz, DMSO-d_6, 298 K): δ (ppm) = 11.35 (s, 1 H), 8.65 (d, J = 16.8 Hz, 1 H), 8.02 (d, J = 7.6 Hz, 1 H), 7.96 (d, J = 16.6 Hz, 1 H), 7.85 (d, J = 7.4 Hz, 1 H), 7.61 (m, 2 H), 7.44 (t, J = 8.2 Hz, 1 H), 6.66 (dd, J = 8.2, 3.9 Hz, 2 H), 4.65 (t, J = 7.8 Hz, 2 H), 4.03 (s, 3 H), 2.67 (t, J = 6.7 Hz, 2 H), 2.17 (m, 2 H), 1.77 (s, 6 H); ^{13}C NMR (125 MHz, DMSO-d_6, 298 K): δ (ppm) = 182.3, 161.5, 160.6, 145.8, 143.2, 141.0, 136.6, 129.1, 128.8, 122.9, 114.9, 112.9, 110.9, 108.6, 102.5, 56.5, 51.6, 47.9, 45.9, 26.8, 24.2; ESI-HRMS calcd. for $C_{22}H_{24}NO_5S$ $[M - H]^-$: 414.1375, found: 414.1381.

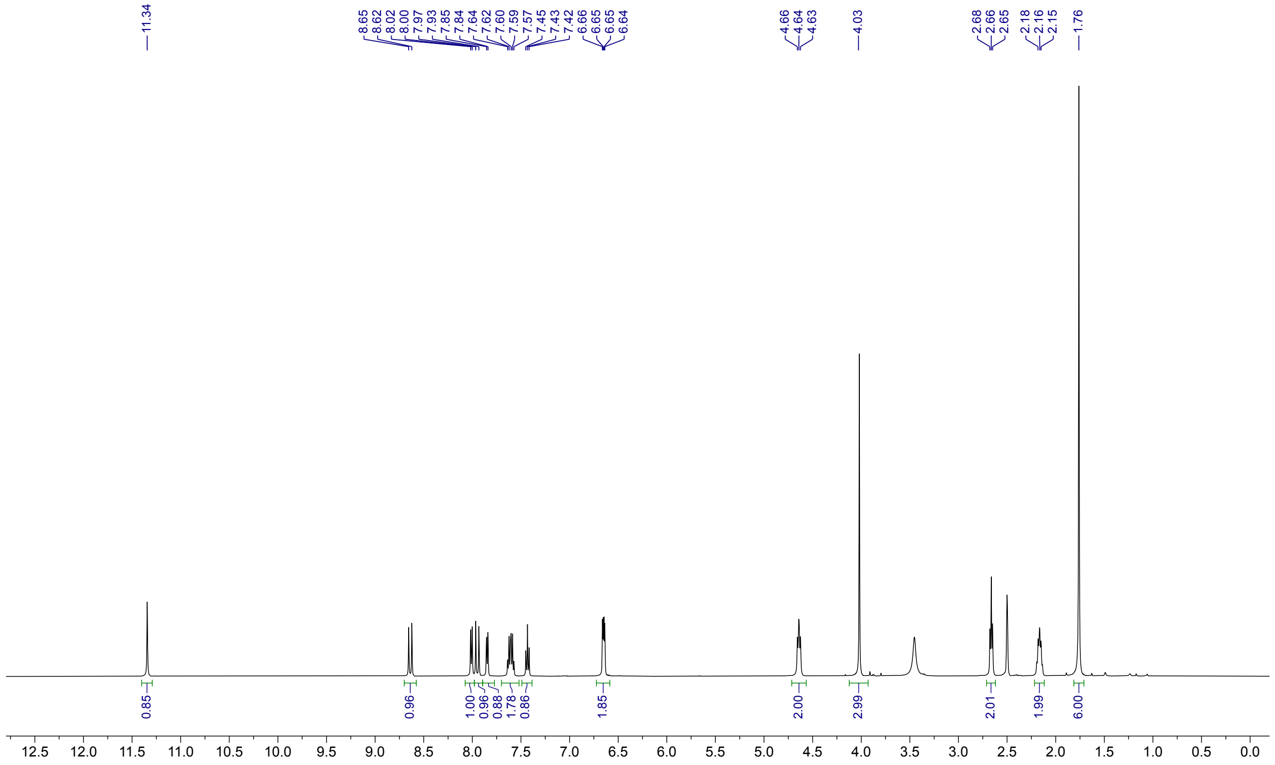

Figure S7. ^{1}H NMR spectrum of **4** (500 MHz, DMSO-d_6, 298 K).

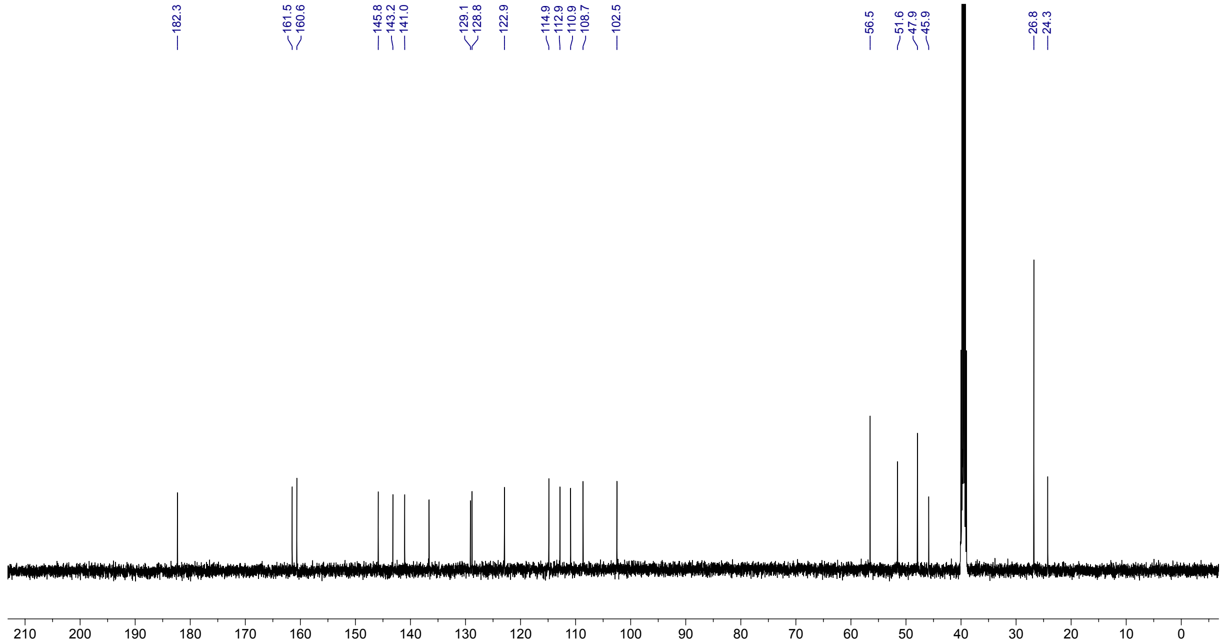

Figure S8. ^{13}C NMR spectrum of **4** (125 MHz, DMSO-d_6, 298 K).

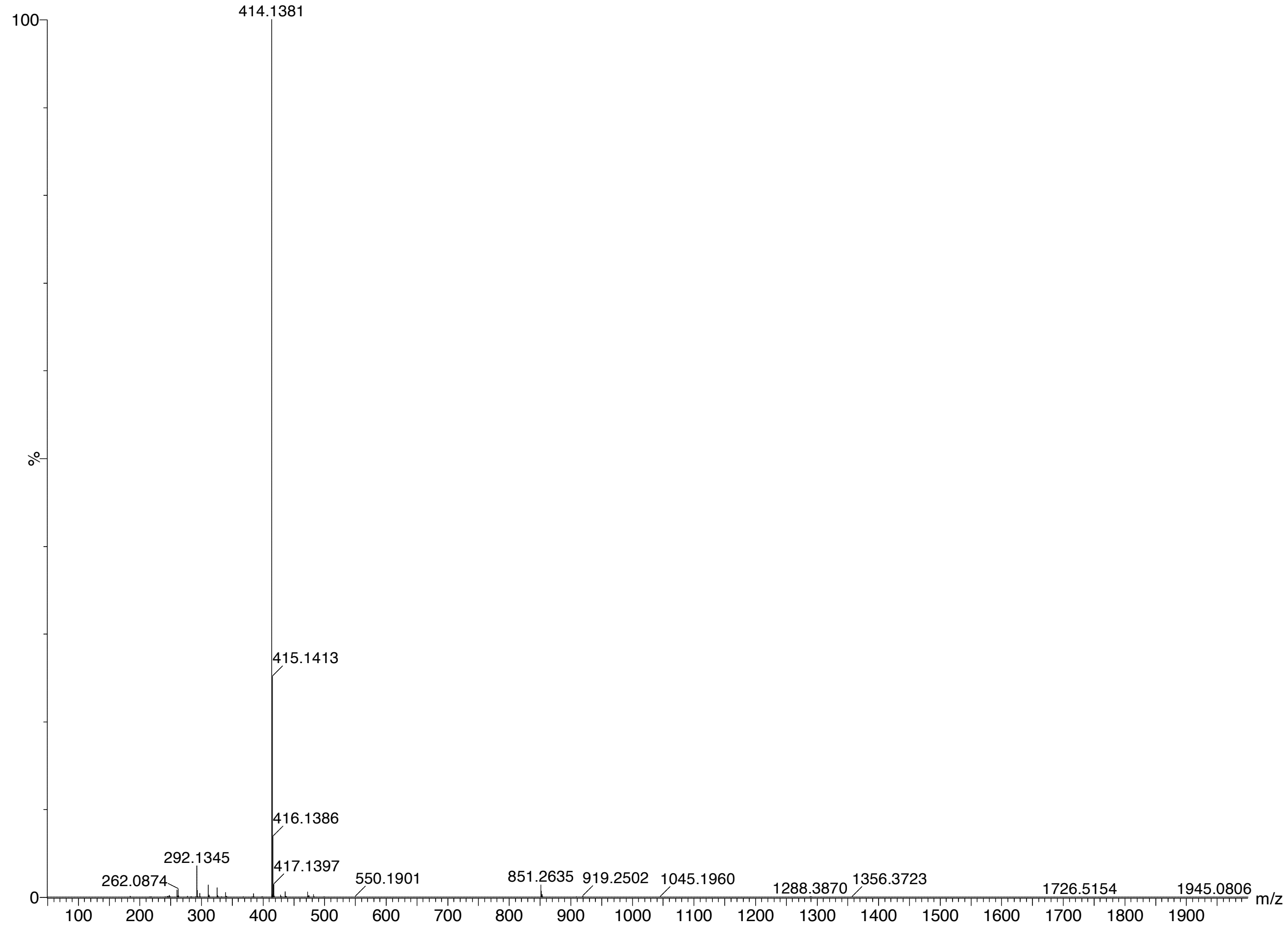

Figure S9. A high-resolution ESI-TOF mass spectrum of **4**.

Spiropyran **5**:[9] Prepared according to the general procedure (see above), starting with 252 mg (0.90 mmol) of **S0** and 132 mg (0.94 mmol) of **S5**. Yield = 240 mg (66%). ^{1}H NMR (500 MHz, DMSO-d_6, 298 K): δ (ppm) = 11.62 (s, 1 H), 8.51 (d, J = 16.6 Hz, 1 H), 8.40 (dd, J = 8.4, 6.7 Hz, 1 H), 8.01 (d, J = 7.8 Hz, 1 H), 7.86 (d, J = 16.2 Hz, 1 H), 7.85 (d, J = 7.8 Hz, 1 H), 7.62 (m, 2 H), 6.88 (t, J = 8.7 Hz, 1 H), 6.81 (dd, J = 10.7, 2.3 Hz, 1 H), 4.80 (t, J = 7.2 Hz, 2 H), 2.65 (t. J = 5.8 Hz, 2 H), 2.17 (m, 2 H), 1.76 (s, 6 H); ^{13}C NMR (125 MHz, DMSO-d_6, 298 K): δ (ppm) = 181.7, 167.6, 165.1, 161.0, 160.8, 147.6, 143.4, 140.9, 132.5, 132.4, 129.1, 129.1, 123.0, 118.5, 118.5, 115.0, 111.2, 108.1, 107.9, 103.4, 103.2, 51.8, 47.3, 45.5, 26.4, 24.6; ^{19}F NMR (470 MHz, DMSO-d_6, 298 K): δ = –102.6 ppm; ESI-HRMS calcd. for $C_{21}H_{21}FNO_4S$ $[M – H]^{-}$: 402.1175, found: 402.1182.

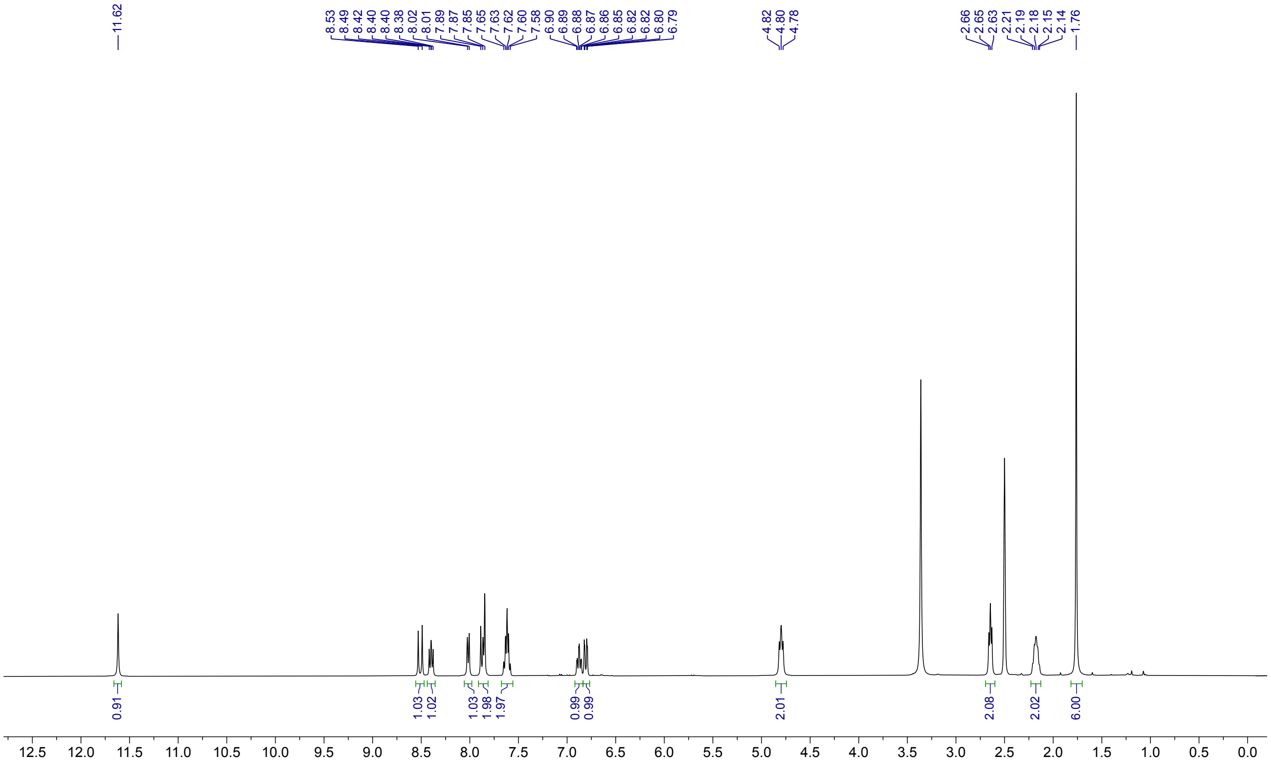

Figure S10. ^{1}H NMR spectrum of **5** (500 MHz, DMSO-d_6, 298 K).

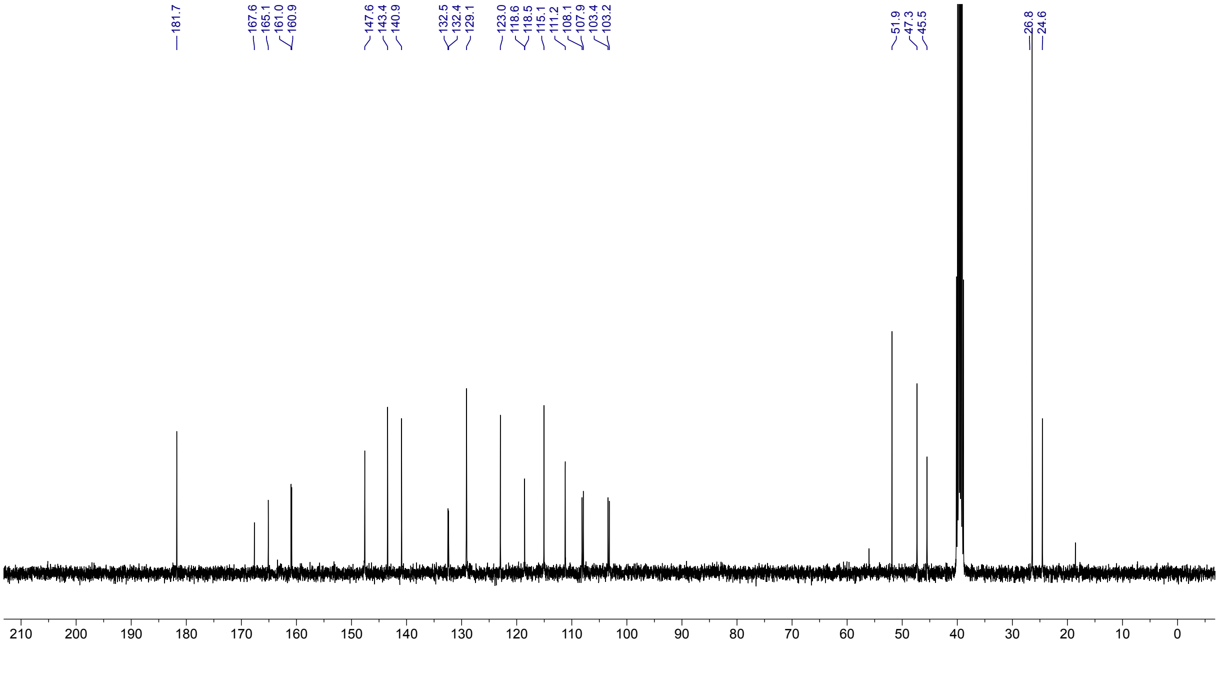

Figure S11. ^{13}C NMR spectrum of **5** (125 MHz, DMSO-d_6, 298 K).

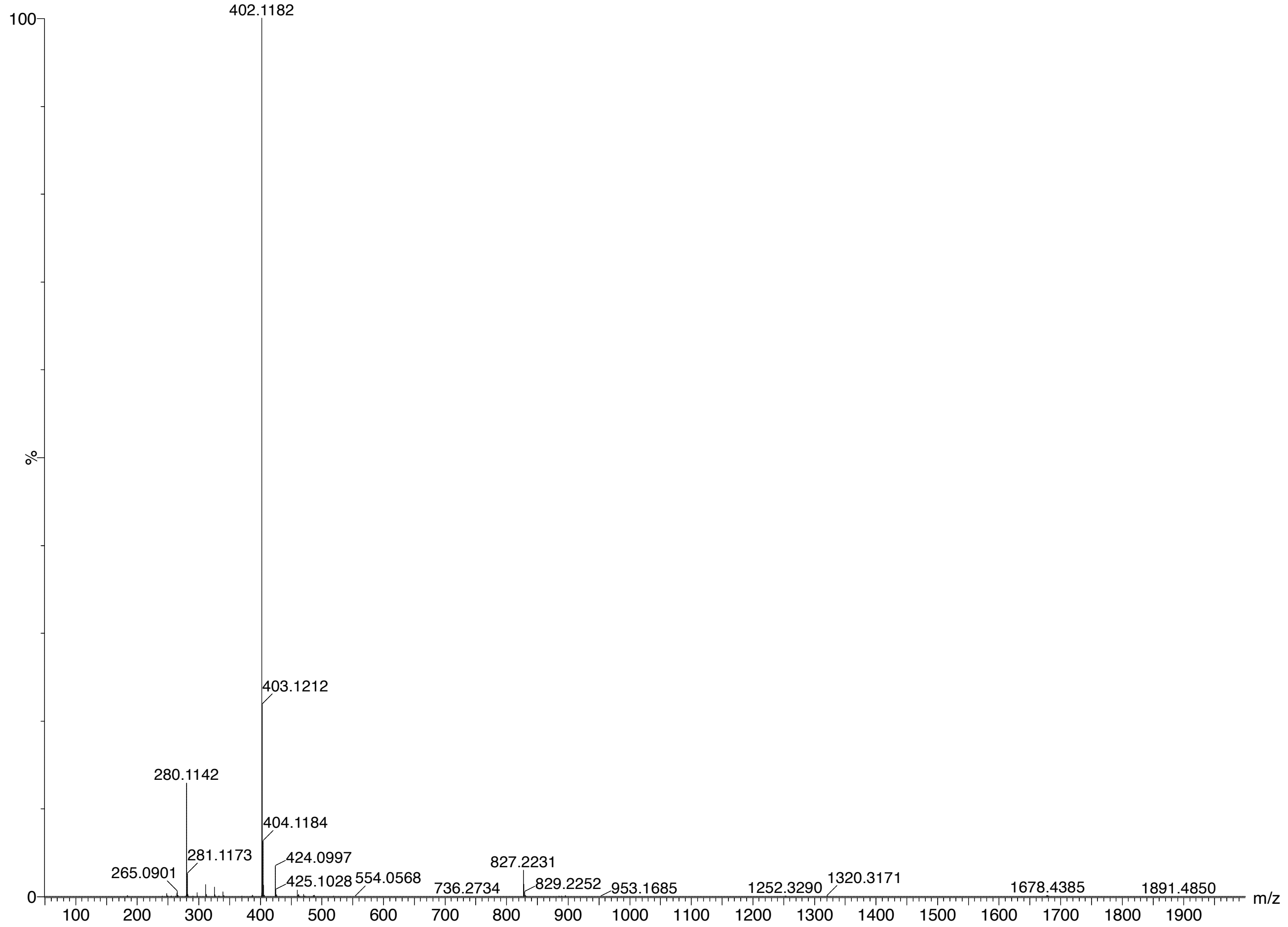

Figure S12. A high-resolution ESI-TOF mass spectrum of **5**.

Spiropyran **6**: Prepared according to the general procedure (see above), starting with 222 mg (0.79 mmol) of **S0** and 150 mg (0.79 mmol) of **S6**. Yield = 180 mg (50%). ^{1}H NMR (500 MHz, DMSO-d_6, 298 K): δ (ppm) = 11.63 (s, 1 H), 8.53 (d, J = 12.2 Hz, 1 H), 8.50 (m, 1 H), 8.08 (m, 1 H), 8.03 (d, J = 16.6 Hz, 1 H), 7.89 (m, 1 H), 7.65 (m, 2 H), 7.30 (s, 1 H), 7.29 (d, J = 7.2 Hz, 1 H), 4.86 (t, J = 7.7 Hz, 2 H), 2.66 (t, J = 6.3 Hz, 2 H), 2.21 (m, 2 H), 1.79 (s, 6 H); ^{13}C NMR (125 MHz, DMSO-d_6, 298 K): δ (ppm) = 181.9, 158.4, 146.3, 143.8, 140.9, 131.0, 129.6, 129.2, 124.8, 123.0, 115.9, 115.5, 114.4, 112.9, 52.2, 47.2, 46.0, 26.1, 24.7; ^{19}F NMR (470 MHz, DMSO-d_6, 298 K): δ = –63.0 ppm; ESI-HRMS calcd. for $C_{22}H_{21}F_3NO_4S$ [M – H]$^-$: 452.1143, found: 452.1147.

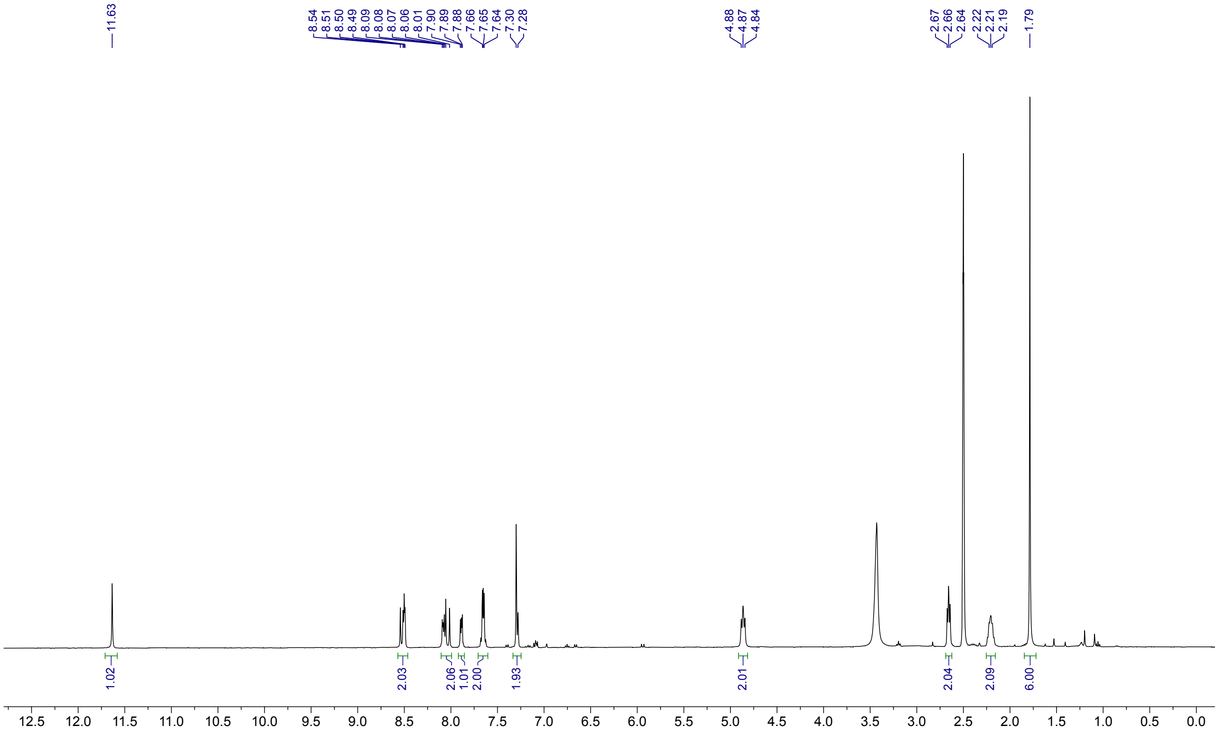

Figure S13. ^{1}H NMR spectrum of **6** (500 MHz, DMSO-d_6, 298 K).

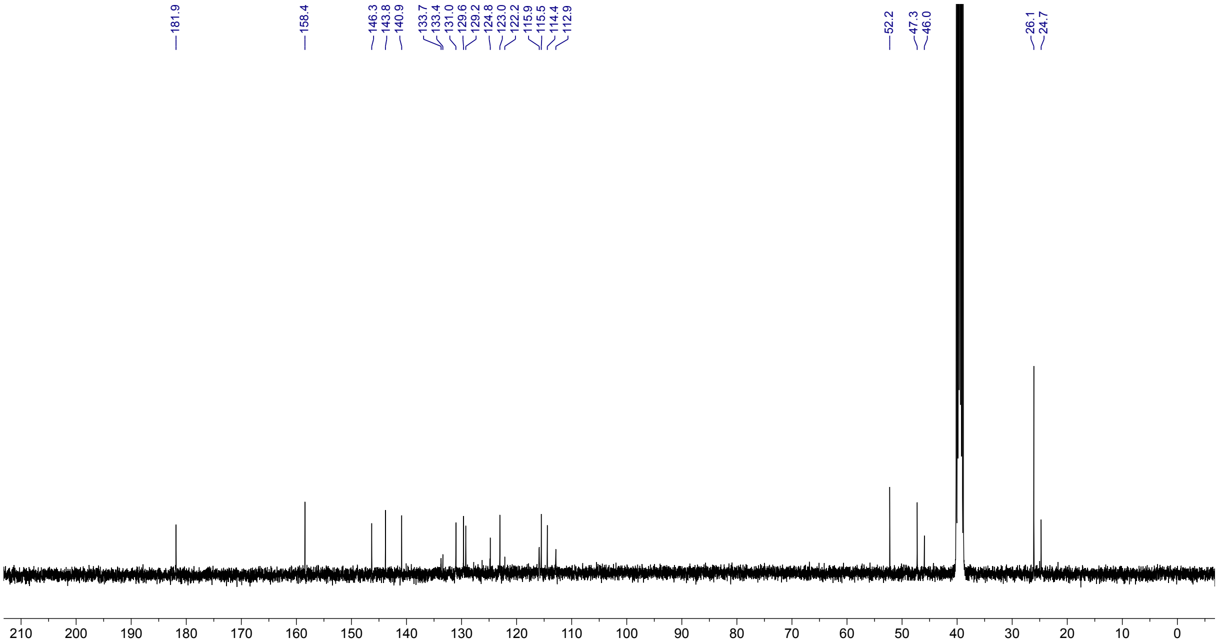

Figure S14. ^{13}C NMR spectrum of **6** (125 MHz, DMSO-d_6, 298 K).

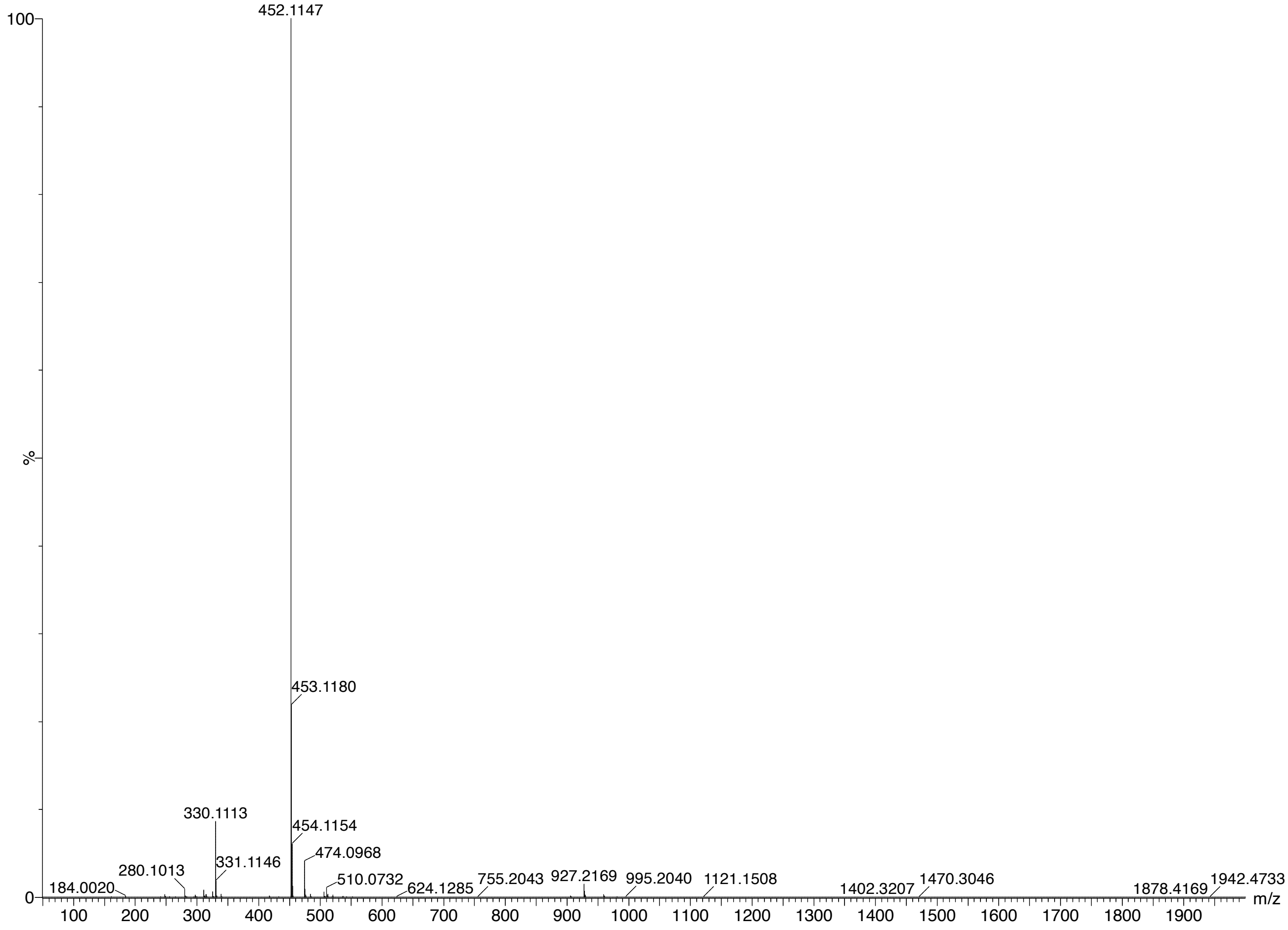

Figure S15. A high-resolution ESI-TOF mass spectrum of **6**.

Spiropyran **7**:[9] Prepared according to the general procedure (see above), starting with 190 mg (0.67 mmol) of **S0** and 104 mg (0.68 mmol) of **S7**. Yield = 210 mg (75%). ^{1}H NMR (400 MHz, DMSO-d_6, 298 K): δ (ppm) = 11.28 (s, 1 H), 8.54 (d, *J* = 16.2 Hz, 1 H), 8.28 (d, *J* = 8.9 Hz, 1 H), 7.93 (d, *J* = 7.8 Hz, 1 H), 7.81 (d, *J* = 7.1 Hz, 1 H), 7.71 (d, *J* = 16.1 Hz, 1 H), 7.60 (t, *J* = 7.1 Hz, 1 H), 7.55 (t, *J* = 7.4 Hz, 1 H), 6.63 (dd, *J* = 8.9, 2.2 Hz, 1 H), 6.55 (d, *J* = 2.2 Hz, 1 H), 4.72 (t, *J* = 7.6 Hz, 2 H), 3.85 (s, 3 H), 2.64 (t, *J* = 6.4 Hz, 2 H), 2.15 (m, 2 H), 1.74 (s, 6 H); ^{13}C NMR (100 MHz, DMSO-d_6, 298 K): δ (ppm) = 181.0, 166.2, 161.7, 148.8, 143.0, 141.0, 131.9, 129.0, 128.4, 122.9, 115.3, 114.4, 108.4, 108.1, 100.6, 55.7, 51.3, 47.4, 44.9, 26.8, 24.3; ESI-HRMS calcd. for $C_{22}H_{24}NO_5S$ [M – H]$^-$: 414.1375, found: 414.1381.

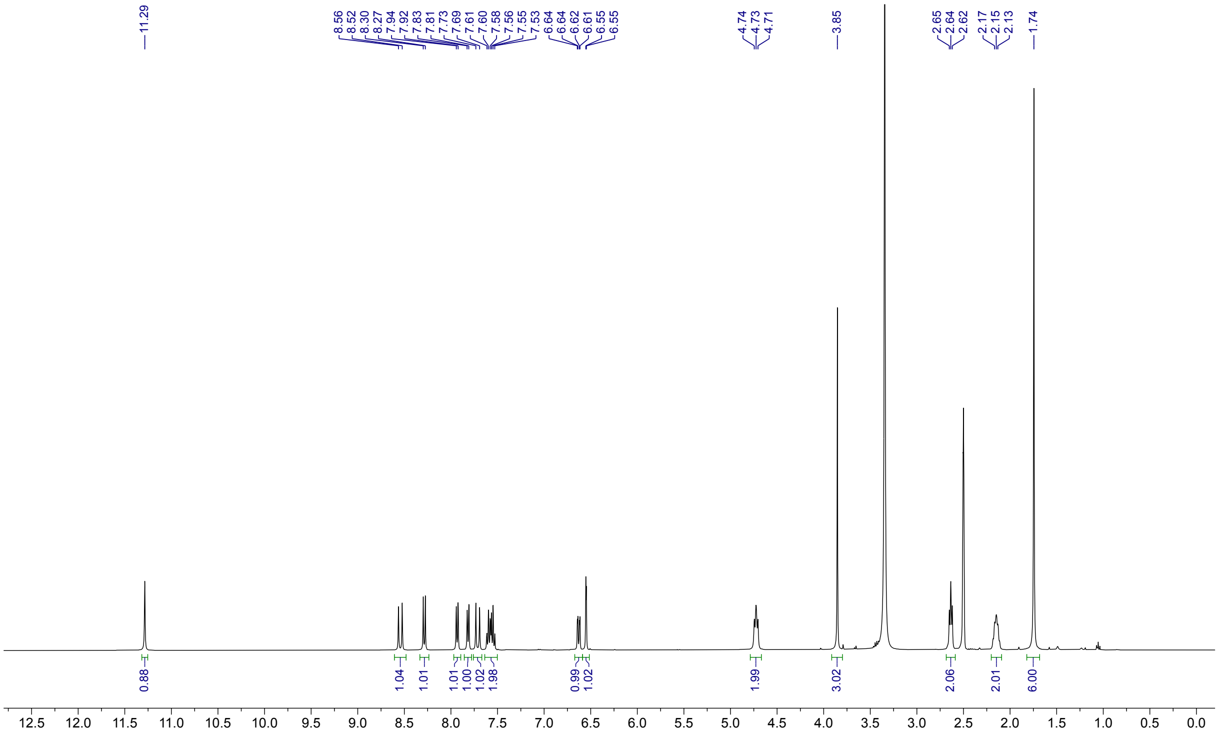

Figure S16. ^{1}H NMR spectrum of **7** (400 MHz, DMSO-d_6, 298 K).

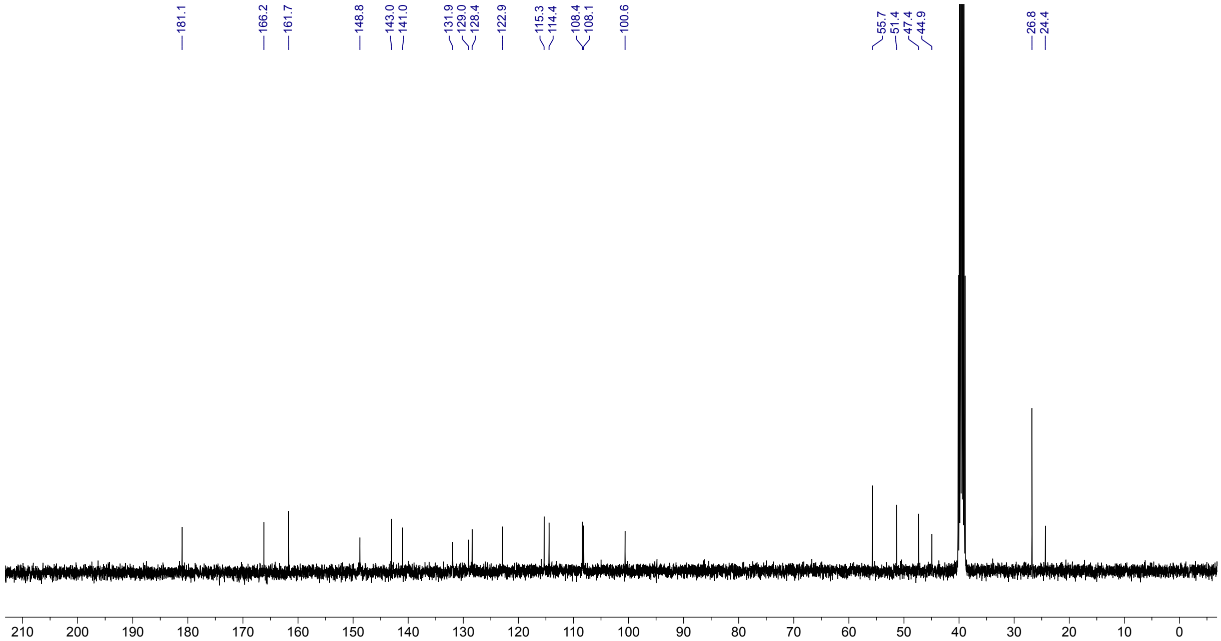

Figure S17. ^{13}C NMR spectrum of **7** (100 MHz, DMSO-d_6, 298 K).

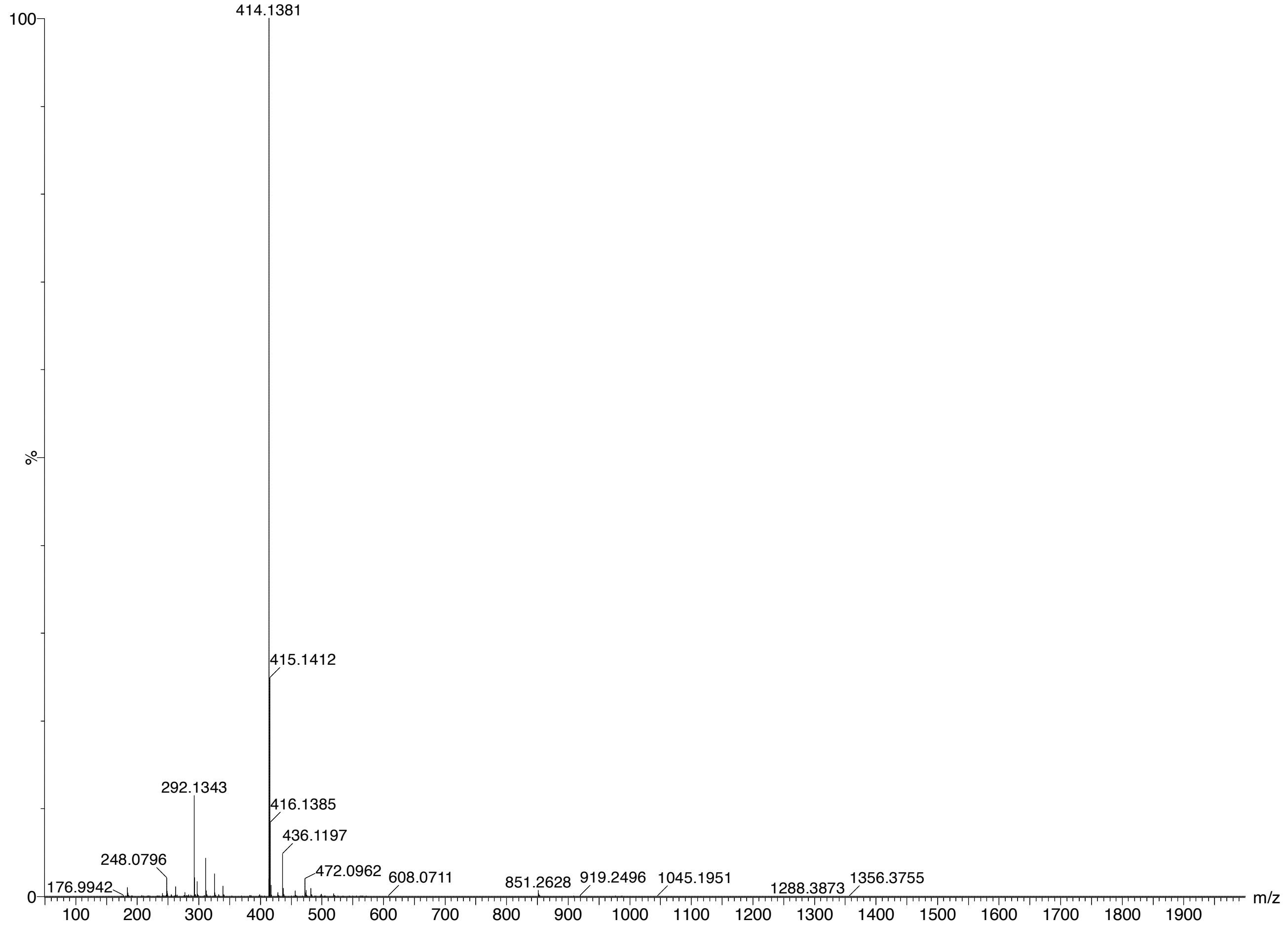

Figure S18. A high-resolution ESI-TOF mass spectrum of **7**.

Spiropyran **8**: Prepared according to the general procedure (see above), starting with 160 mg (0.56 mmol) of **S0** and 102 mg (0.57 mmol) of **S8**. Yield = 160 mg (64%). ^{1}H NMR (500 MHz, DMSO-d_6, 298 K): δ (ppm) = 11.39 (s, 1 H), 8.54 (d, *J* = 16.3 Hz, 1 H), 8.41 (d, *J* = 8.2 Hz, 1 H), 8.08 (m, 1 H), 8.01 (d, *J* = 16.5 Hz, 1 H), 7.88 (m, 1 H), 7.63 (m, 3 H), 7.49 (d, *J* = 8.3 Hz, 1 H), 4.85 (t, *J* = 7.5 Hz, 2 H), 3.88 (s, 3 H), 2.66 (t, *J* = 6.2 Hz, 2 H), 2.20 (m, 2 H), 1.78 (s, 6 H); ^{13}C NMR (125 MHz, DMSO-d_6, 298 K): δ (ppm) = 181.8, 165.4, 158.4, 146.8, 143.8, 140.9, 134.5, 130.1, 129.6, 129.2, 125.2, 123.0, 119.9, 116.8, 115.4, 114.1, 52.5, 52.2, 47.3, 45.9, 26.1, 24.7; ESI-HRMS calcd. for $C_{23}H_{24}NO_6S$ $[M – H]^–$: 442.1324, found: 442.1331.

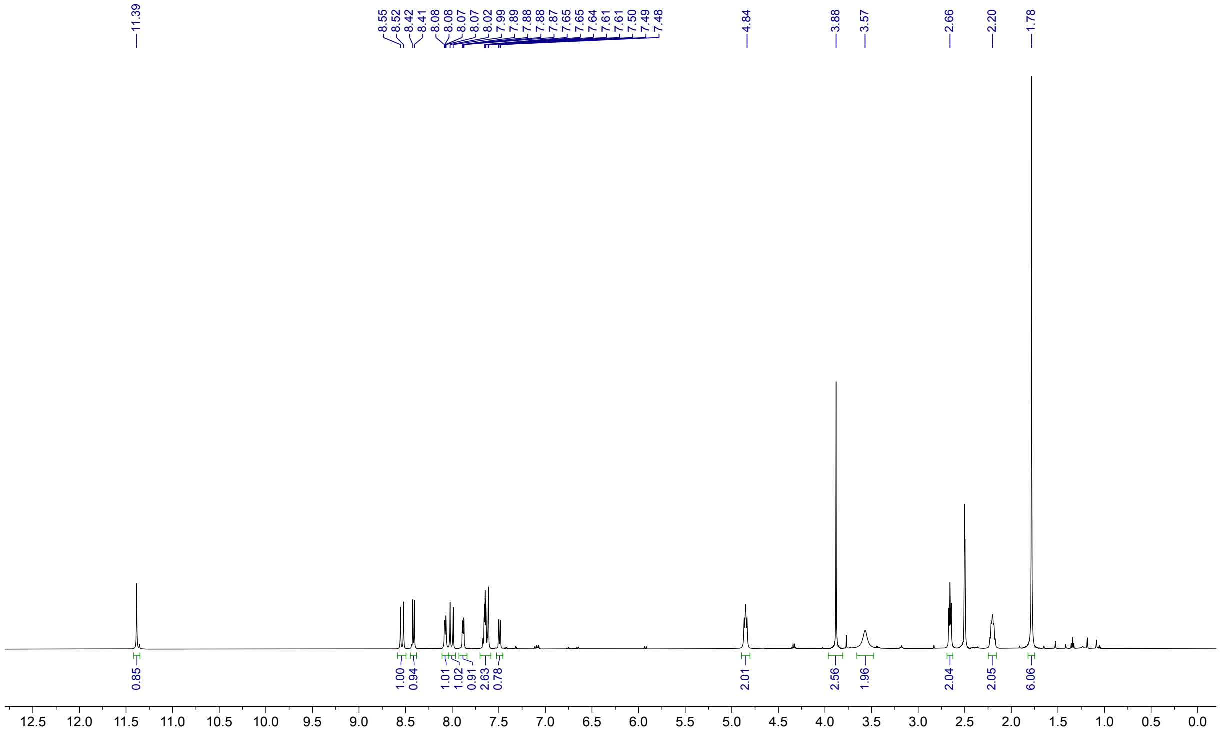

Figure S19. ^{1}H NMR spectrum of **8** (500 MHz, DMSO-d_6, 298 K).

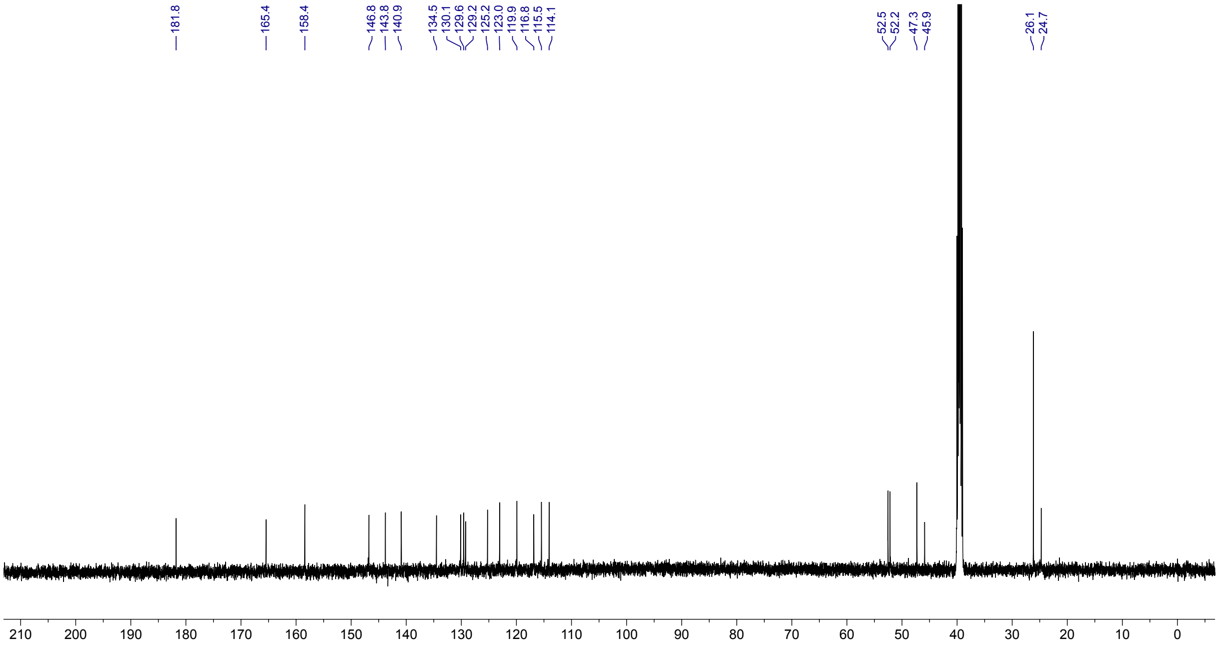

Figure S20. ^{13}C NMR spectrum of **8** (125 MHz, DMSO-d_6, 298 K).

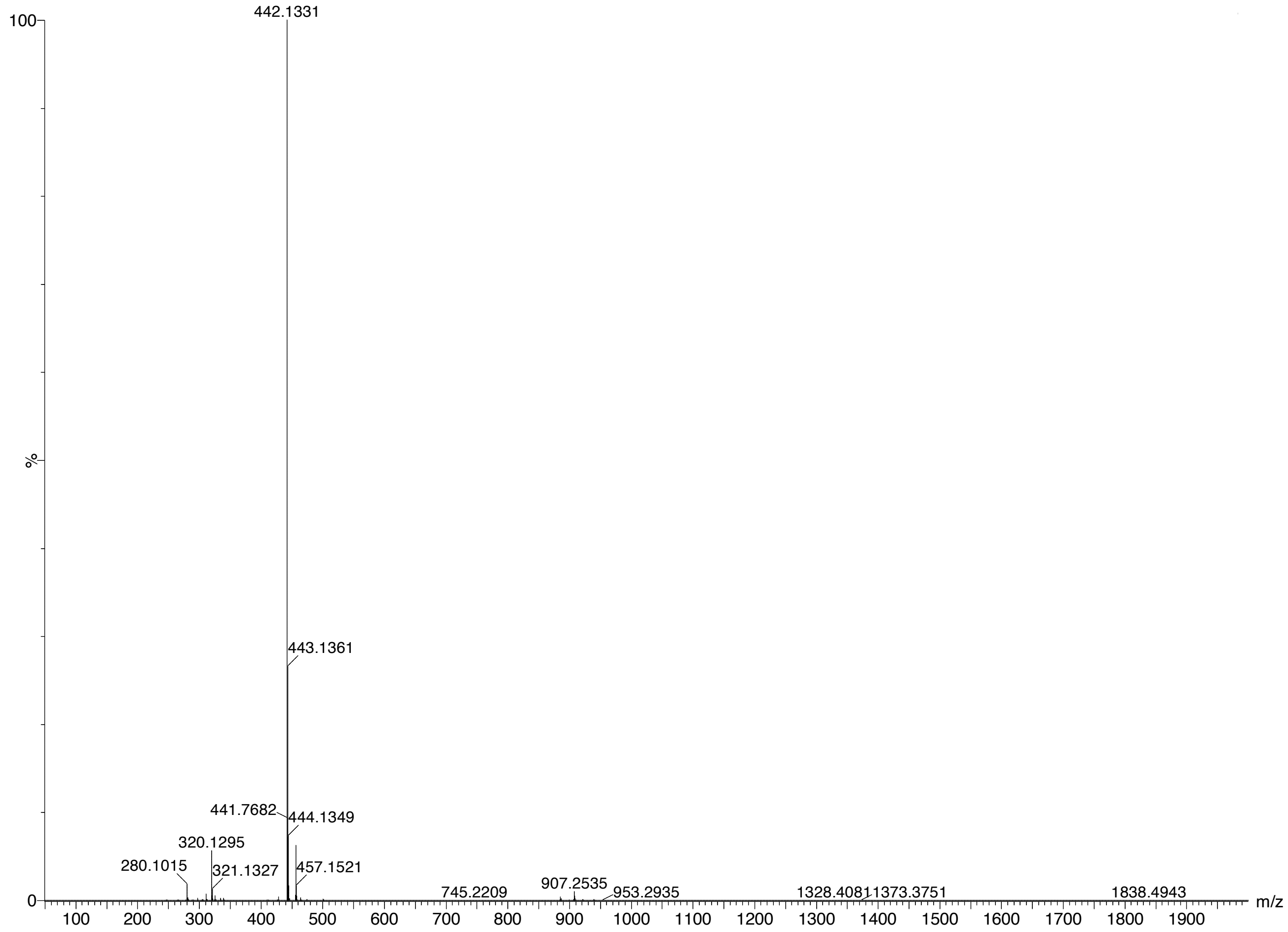

Figure S21. A high-resolution ESI-TOF mass spectrum of spiropyran **8**.

3. Encapsulation of spiropyrans studied by UV/vis absorption spectroscopy

General comments: UV/vis titration experiments were performed by adding aliquots of stock solutions of cage **1** and **2** (both in H_2O, concentration is 1 mM) into the solutions of spiropyrans (50 µM in H_2O with 5% MeOH, 1 mM stock solution in MeOH). UV/vis spectra of each addition were recorded after equilibrium reached. Binding stoichiometry was determined to be 1:1 based on Job's plot. Binding constants for the inclusion complexes were estimated by non-linear curve fitting of the titration traces of the maximum absorbance based on 1:1 binding model. The relationship between observed changes of absorbance (ΔA) and the concentration of the host (C_{host}) is given bellow according to 1:1 binding model:[2,10,11]

$$\Delta A = \frac{L \cdot (1 + K_{\text{assoc}} \cdot C_G + K_{\text{assoc}} \cdot C_H) - \sqrt{L^2 \cdot (1 + K_{\text{assoc}} \cdot C_G + K_{\text{assoc}} \cdot C_H)^2 - 4L^2 \cdot K_{\text{assoc}}{}^2 \cdot C_G \cdot C_H}}{2K_{\text{assoc}} \cdot C_G}$$

where K_{assoc} is the binding constant, L is the maximum change of absorbance, and C_H and C_G are the concentrations of the host and guest, respectively.

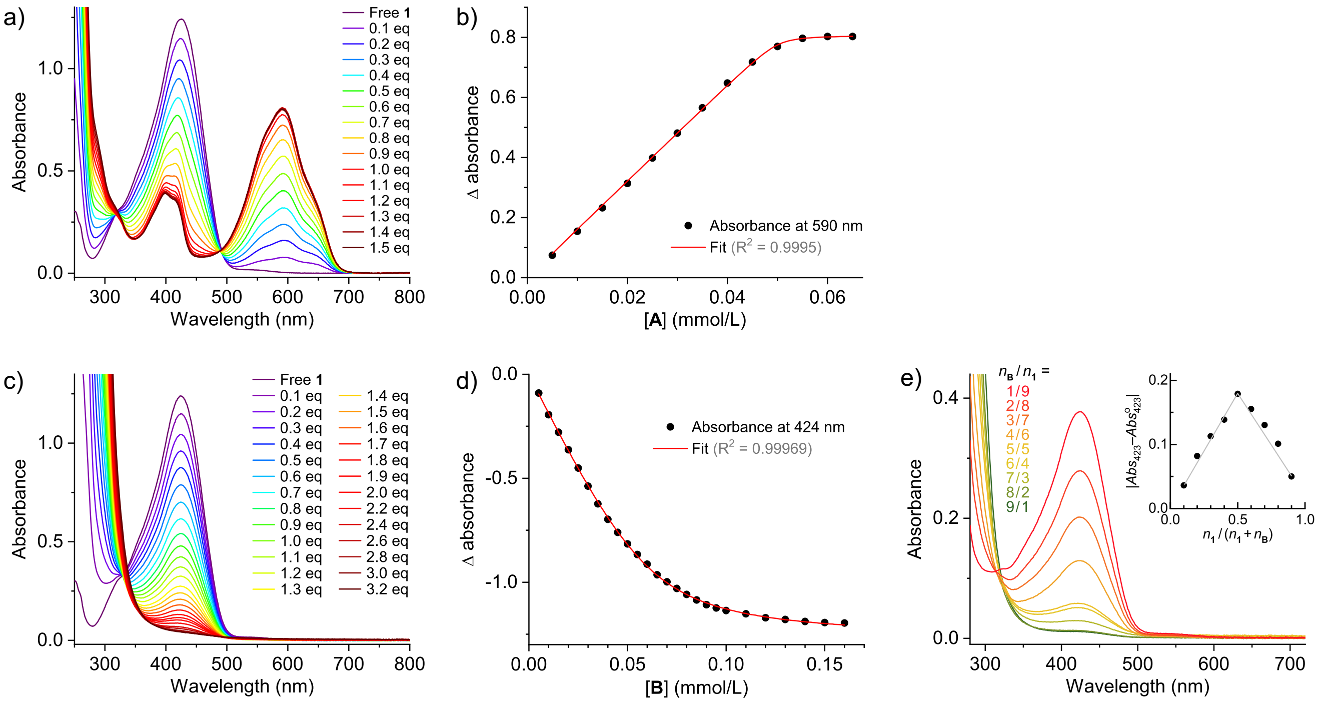

Figure S22. Studying the complexation of spiropyran **1** by cages **A** and **B**. (a) UV/vis absorption spectra (dilution-corrected) of spiropyran **1** (initial concentration = 50 µM) in the presence of increasing amounts of cage **A**. (b) Changes in the absorbance at 590 nm (the wavelength of maximum absorption of **1**'s MC form within **A**) in the presence of increasing amounts of **A**. Nonlinear curve fitting based on a 1:1 binding model gives an association constant (K_{assoc}) of 1.20(±0.63)×10^7 M^{-1} (error estimated from fitting). (c) UV/vis spectra (dilution-corrected) of **1** (initial concentration = 50 µM) in the presence of increasing amounts of cage **B**. (d) Changes in the absorbance at 424 nm (the wavelength of maximum absorption of **1**'s MCH form) in the presence of increasing amounts of **B**. Nonlinear curve fitting based on a 1:1 binding model gives a K_{assoc} of 1.67(±0.10)×10^5 M^{-1} (error estimated from fitting). (e) A series of UV/vis spectra at different molar ratios of **1** to **B**. The resulting Job's plot (inset) confirms the 1:1 stoichiometry of the inclusion complex. $|Abs_{423}–Abs^{o}_{423}|$ denotes the absolute difference between the observed absorbance at 423 nm and the absorbance at 423 nm of free **1** at the same concentration.

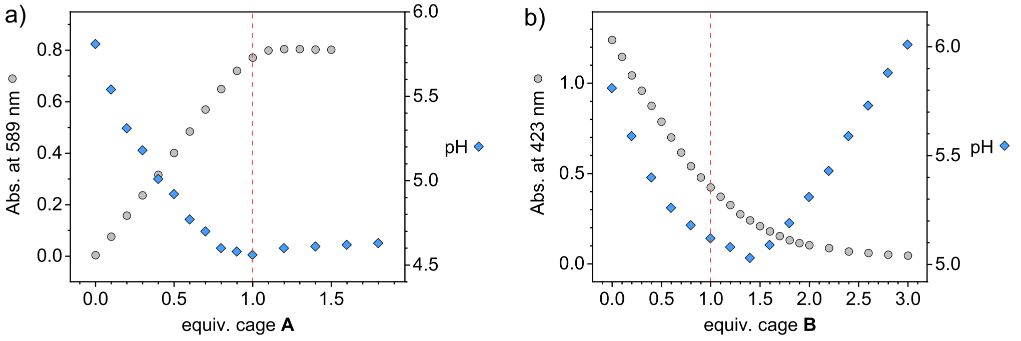

Figure S23. Monitoring the pH during titration of spiropyran **1** with (a) cage **A** and (b) cage **B**. The initial pH = 5.81 (for 50 μM $\mathbf{1}_{MCH}$; initial volume = 1000 μL); titration with 1 mM solutions of cage **A** and **B** (pH = 7.28 and 7.54, respectively); each 0.1 equiv aliquot of the cage = 50 μL. Note that at 1.0 equiv of cage **A**, c_1 = 47.6 μM; a quantitative deprotonation of a strong acid at this concentration would give rise to pH = 4.32 – i.e., close to the observed pH = 4.56 (increased slightly by the addition of the weakly basic **A**). The other spiropyrans behaved analogously.

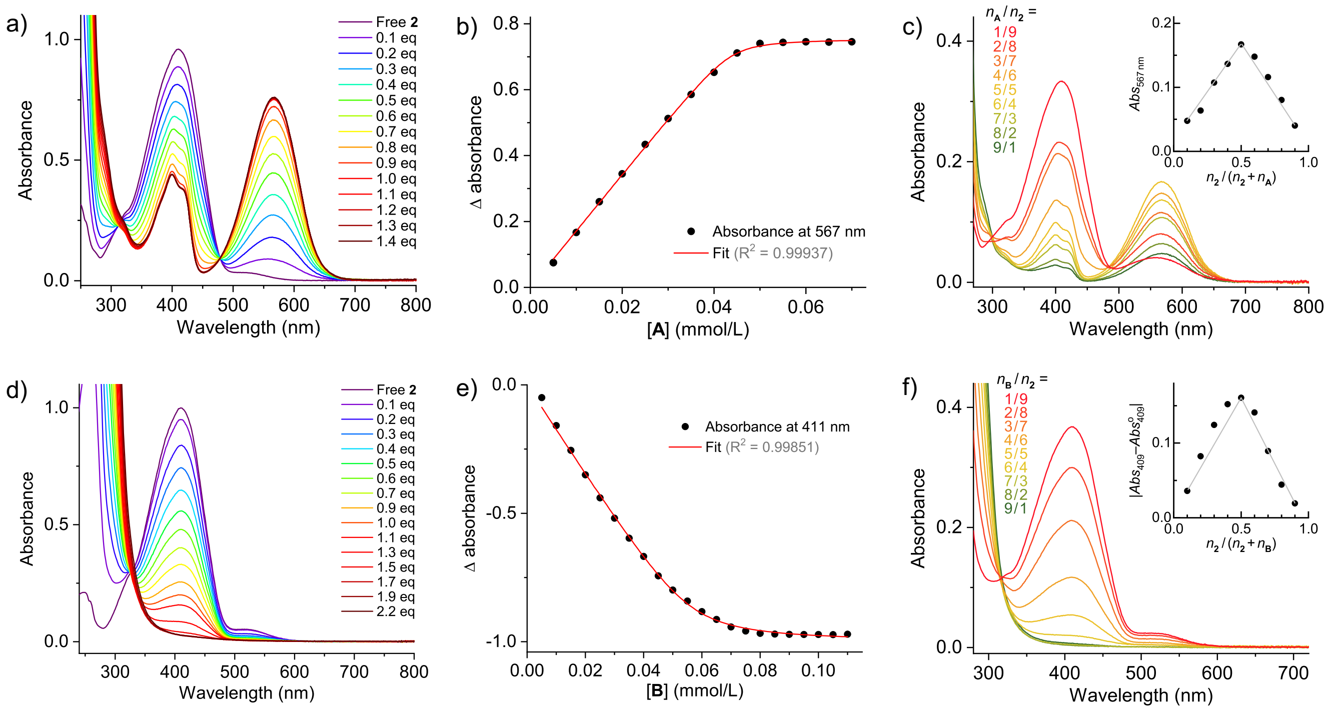

Figure S24. Studying the complexation of spiropyran **2** by cages **A** and **B**. (a) UV/vis spectra (dilution-corrected) of spiropyran **2** (initial concentration = 50 μM) in the presence of increasing amounts of cage **A**. (b) Changes in the absorbance at 567 nm (the wavelength of maximum absorption of $\mathbf{2}_{MC}$ within **A**) in the presence of increasing amounts of **A**. Nonlinear curve fitting based on a 1:1 binding model gives a K_{assoc} of 4.16(±1.39)×10^6 M^{-1} (error estimated from fitting). (c) A series of UV/vis spectra at several different molar ratios of **2** to **A**. The resulting Job's plot (inset) confirms the 1:1 stoichiometry of the inclusion complex. (d) UV/vis spectra (dilution-corrected) of **2** (initial concentration = 50 μM) in the presence of increasing amounts of cage **B**. (e) Changes in the absorbance at 409 nm (the wavelength of maximum absorption of **2**'s MCH form) in the presence of increasing amounts of **B**. Nonlinear curve fitting based on a 1:1 binding model gives a K_{assoc} of 7.67(±1.65)×10^5 M^{-1} (error estimated from fitting). (f) A series of UV/vis spectra at different molar ratios of **2** to **B**. The resulting Job's plot (inset) confirms the 1:1 stoichiometry of the inclusion complex. $|Abs_{409}–Abs°_{409}|$ denotes the absolute difference between the observed absorbance at 409 nm and the absorbance at 409 nm of free **2** at the same concentration.

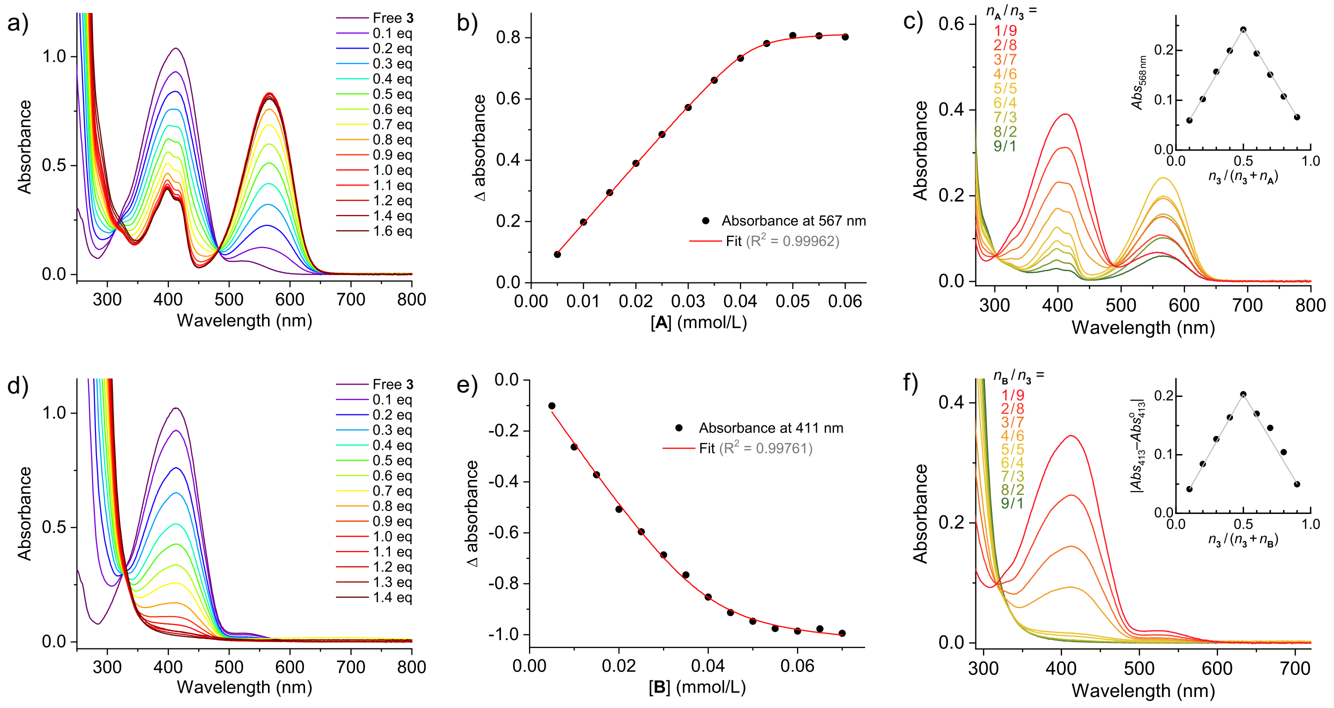

Figure S25. Studying the complexation of spiropyran **3** by cages **A** and **B**. (a) UV/vis spectra (dilution-corrected) of spiropyran **3** (initial concentration = 50 μM) in the presence of increasing amounts of cage **A**. (b) Changes in the absorbance at 567 nm (the wavelength of maximum absorption of $\mathbf{3}_{\mathrm{MC}}$ within **A**) in the presence of increasing amounts of **A**. Nonlinear curve fitting based on a 1:1 binding model gives a K_{assoc} of 3.57(±0.91)×10^6 M^{-1} (error estimated from fitting). (c) A series of UV/vis spectra at several different molar ratios of **3** to **A**. The resulting Job's plot (inset) confirms the 1:1 stoichiometry of the inclusion complex. (d) UV/vis spectra (dilution-corrected) of **3** (initial concentration = 50 μM) in the presence of increasing amounts of cage **B**. (e) Changes in the absorbance at 411 nm (the wavelength of maximum absorption of **3**'s MCH form) in the presence of increasing amounts of **B**. Nonlinear curve fitting based on a 1:1 binding model gives a K_{assoc} of 5.54(±1.76)×10^5 M^{-1} (error estimated from fitting). (f) A series of UV/vis spectra at different molar ratios of **3** to **B**. The resulting Job's plot (inset) confirms the 1:1 stoichiometry of the inclusion complex. $|Abs_{413}–Abs^{\mathrm{o}}_{413}|$ denotes the absolute difference between the observed absorbance at 413 nm and the absorbance at 413 nm of free **3** at the same concentration.

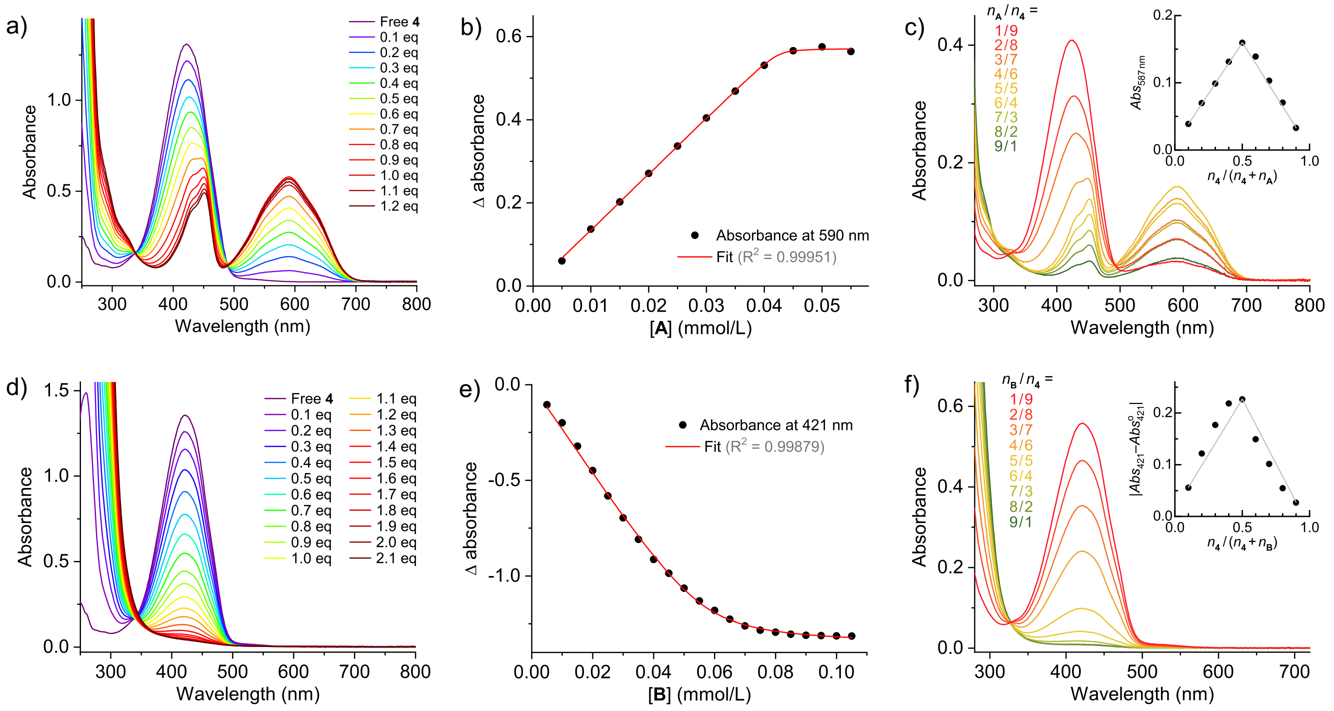

Figure S26. Studying the complexation of spiropyran **4** by cages **A** and **B**. (a) UV/vis spectra (dilution-corrected) of spiropyran **4** (initial concentration = 50 μM) in the presence of increasing amounts of cage **A**. (b) Changes in the absorbance at 590 nm (the wavelength of maximum absorption of $\mathbf{4}_{\mathrm{MC}}$ within **A**) in the presence of increasing amounts of **A**. Nonlinear curve fitting based on a 1:1 binding model gives a K_{assoc} of 2.81(±2.53)×10^7 M^{-1} (error estimated from fitting). (c) A series of UV/vis spectra at several different molar ratios of **4** to **A**. The resulting Job's plot (inset) confirms the 1:1 stoichiometry of the inclusion complex. (d) UV/vis spectra (dilution-corrected) of **4** (initial concentration = 50 μM) in the presence of increasing amounts of cage **B**. (e) Changes in the absorbance at 421 nm (the wavelength of maximum absorption of **4**'s MCH form) in the presence of increasing amounts of **B**. Nonlinear curve fitting based on a 1:1 binding model gives a K_{assoc} of 6.80(±1.35)×10^5 M^{-1} (error estimated from fitting). (f) A series of UV/vis spectra at different molar ratios of **4** to **B**. The resulting Job's plot (inset) confirms the 1:1 stoichiometry of the inclusion complex. $|Abs_{421}–Abs^{o}_{421}|$ denotes the absolute difference between the observed absorbance at 421 nm and the absorbance at 421 nm of free **4** at the same concentration.

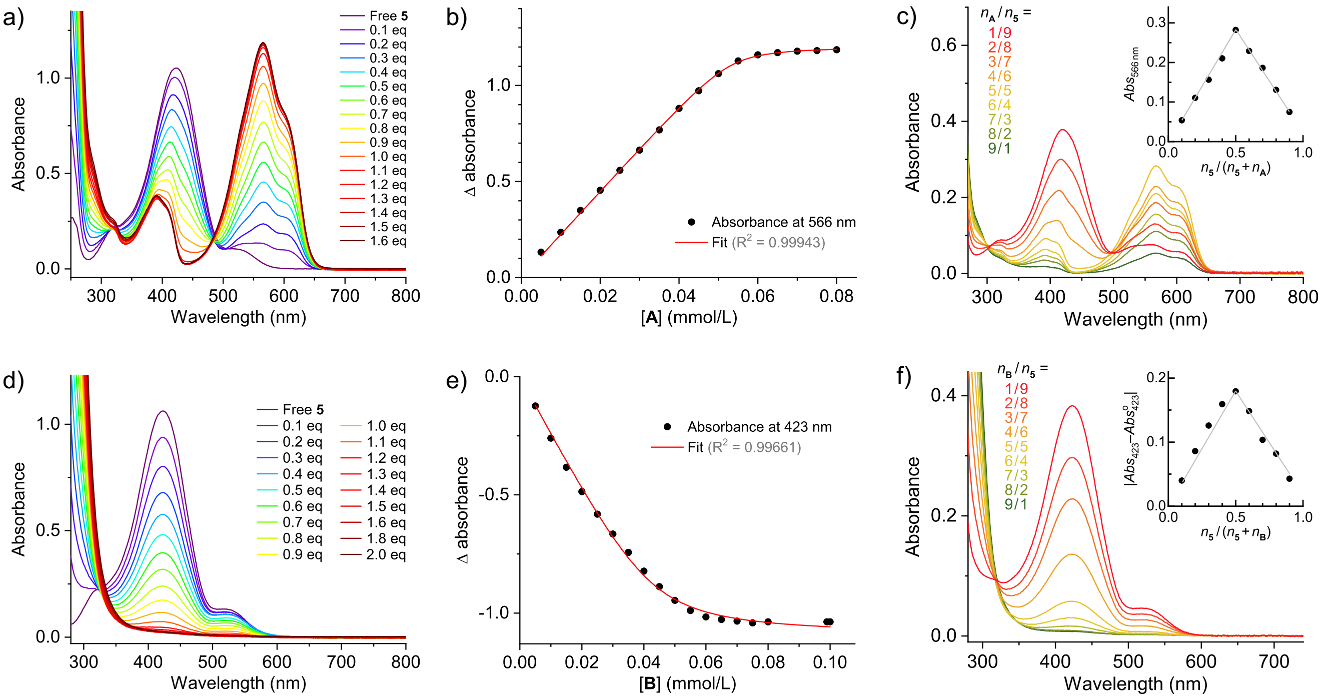

Figure S27. Studying the complexation of spiropyran **5** by cages **A** and **B**. (a) UV/vis spectra (dilution-corrected) of spiropyran **5** (initial concentration = 50 μM) in the presence of increasing amounts of cage **A**. (b) Changes in the absorbance at 566 nm (the wavelength of maximum absorption of $\mathbf{5}_{MC}$ within **A**) in the presence of increasing amounts of **A**. Nonlinear curve fitting based on a 1:1 binding model gives a K_{assoc} of 2.43(±0.60)×10^6 M^{-1} (error estimated from fitting). (c) A series of UV/vis spectra at several different molar ratios of **5** to **A**. The resulting Job's plot (inset) confirms the 1:1 stoichiometry of the inclusion complex. (d) UV/vis spectra (dilution-corrected) of **5** (initial concentration = 50 μM) in the presence of increasing amounts of cage **B**. (e) Changes in the absorbance at 423 nm (the wavelength of maximum absorption of **5**'s MCH form) in the presence of increasing amounts of **B**. Nonlinear curve fitting based on a 1:1 binding model gives a K_{assoc} of 4.62(±1.20)×10^5 M^{-1} (error estimated from fitting). (f) A series of UV/vis spectra at different molar ratios of **5** to **B**. The resulting Job's plot (inset) confirms the 1:1 stoichiometry of the inclusion complex. $|Abs_{423}–Abs^{o}_{423}|$ denotes the absolute difference between the observed absorbance at 423 nm and the absorbance at 423 nm of free **5** at the same concentration.

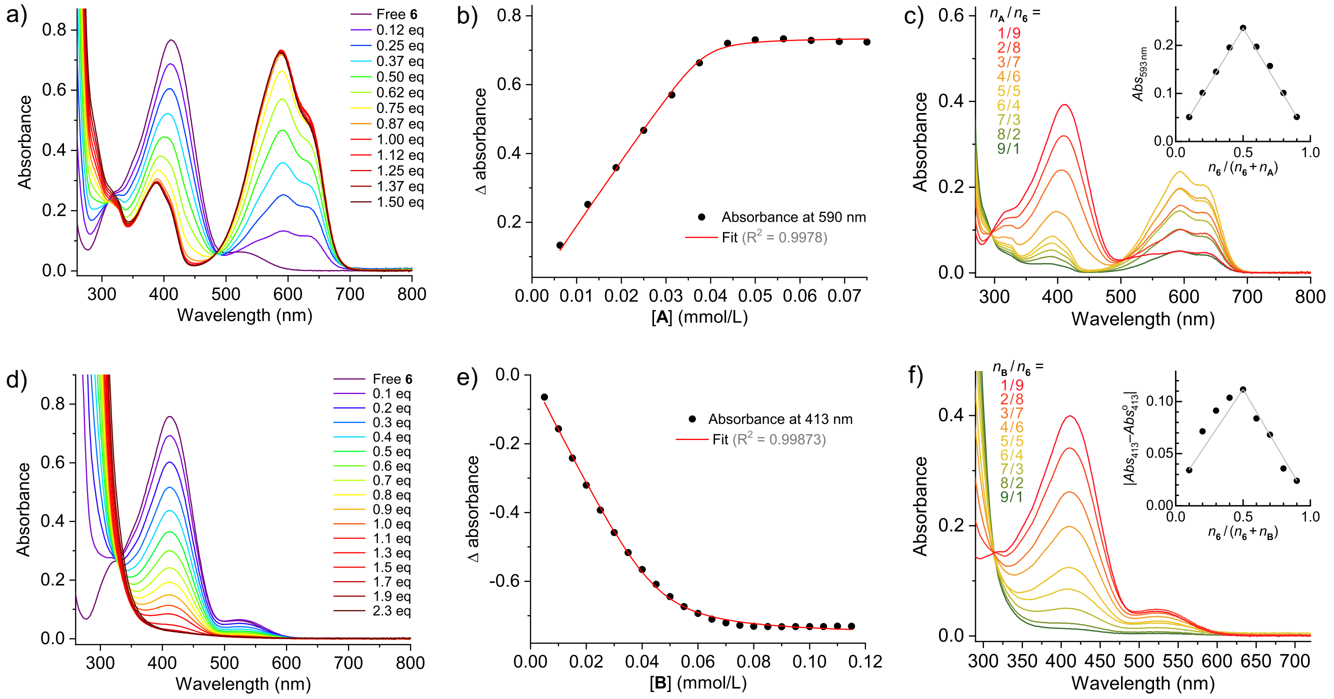

Figure S28. Studying the complexation of spiropyran **6** by cages **A** and **B**. (a) UV/vis spectra (dilution-corrected) of spiropyran **6** (initial concentration = 50 μM) in the presence of increasing amounts of cage **A**. (b) Changes in the absorbance at 590 nm (the wavelength of maximum absorption of $\mathbf{6}_{\mathrm{MC}}$ within **A**) in the presence of increasing amounts of **A**. Nonlinear curve fitting based on a 1:1 binding model gives a K_{assoc} of $3.90(\pm1.98)\times10^{6}$ M^{-1} (error estimated from fitting). (c) A series of UV/vis spectra at several different molar ratios of **6** to **A**. The resulting Job's plot (inset) confirms the 1:1 stoichiometry of the inclusion complex. (d) UV/vis spectra (dilution-corrected) of **6** (initial concentration = 50 μM) in the presence of increasing amounts of cage **B**. (e) Changes in the absorbance at 413 nm (the wavelength of maximum absorption of **6**'s MCH form) in the presence of increasing amounts of **B**. Nonlinear curve fitting based on a 1:1 binding model gives a K_{assoc} of $5.36(\pm0.77)\times10^{5}$ M^{-1} (error estimated from fitting). (f) A series of UV/vis spectra at different molar ratios of **6** to **B**. The resulting Job's plot (inset) confirms the 1:1 stoichiometry of the inclusion complex. $|Abs_{413}–Abs^{o}_{413}|$ denotes the absolute difference between the observed absorbance at 413 nm and the absorbance at 413 nm of free **6** at the same concentration.

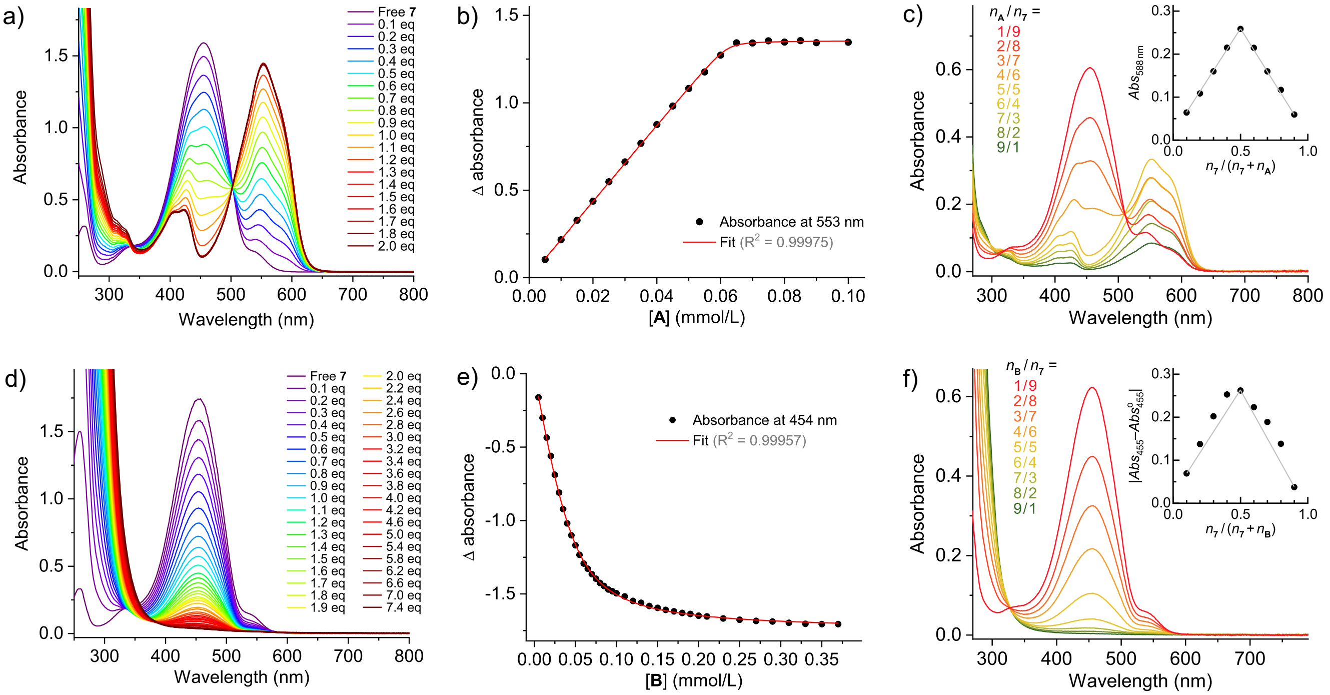

Figure S29. Studying the complexation of spiropyran **7** by cages **A** and **B**. (a) UV/vis spectra (dilution-corrected) of spiropyran **7** (initial concentration = 50 μM) in the presence of increasing amounts of cage **A**. (b) Changes in the absorbance at 590 nm (the wavelength of maximum absorption of $\mathbf{7}_{\mathrm{MC}}$ within **A**) in the presence of increasing amounts of **A**. Nonlinear curve fitting based on a 1:1 binding model gives a K_{assoc} of $1.22(\pm0.41)\times10^{7}$ M^{-1} (error estimated from fitting). (c) A series of UV/vis spectra at several different molar ratios of **7** to **A**. The resulting Job's plot (inset) confirms the 1:1 stoichiometry of the inclusion complex. (d) UV/vis spectra (dilution-corrected) of **7** (initial concentration = 50 μM) in the presence of increasing amounts of cage **B**. (e) Changes in the absorbance at 454 nm (the wavelength of maximum absorption of **7**'s MCH form) in the presence of increasing amounts of **B**. Nonlinear curve fitting based on a 1:1 binding model gives a K_{assoc} of $1.01(\pm0.03)\times10^{5}$ M^{-1} (error estimated from fitting). (f) A series of UV/vis spectra at different molar ratios of **7** to **B**. The resulting Job's plot (inset) confirms the 1:1 stoichiometry of the inclusion complex. $|Abs_{455}–Abs^{o}_{455}|$ denotes the absolute difference between the observed absorbance at 455 nm and the absorbance at 455 nm of free **7** at the same concentration.

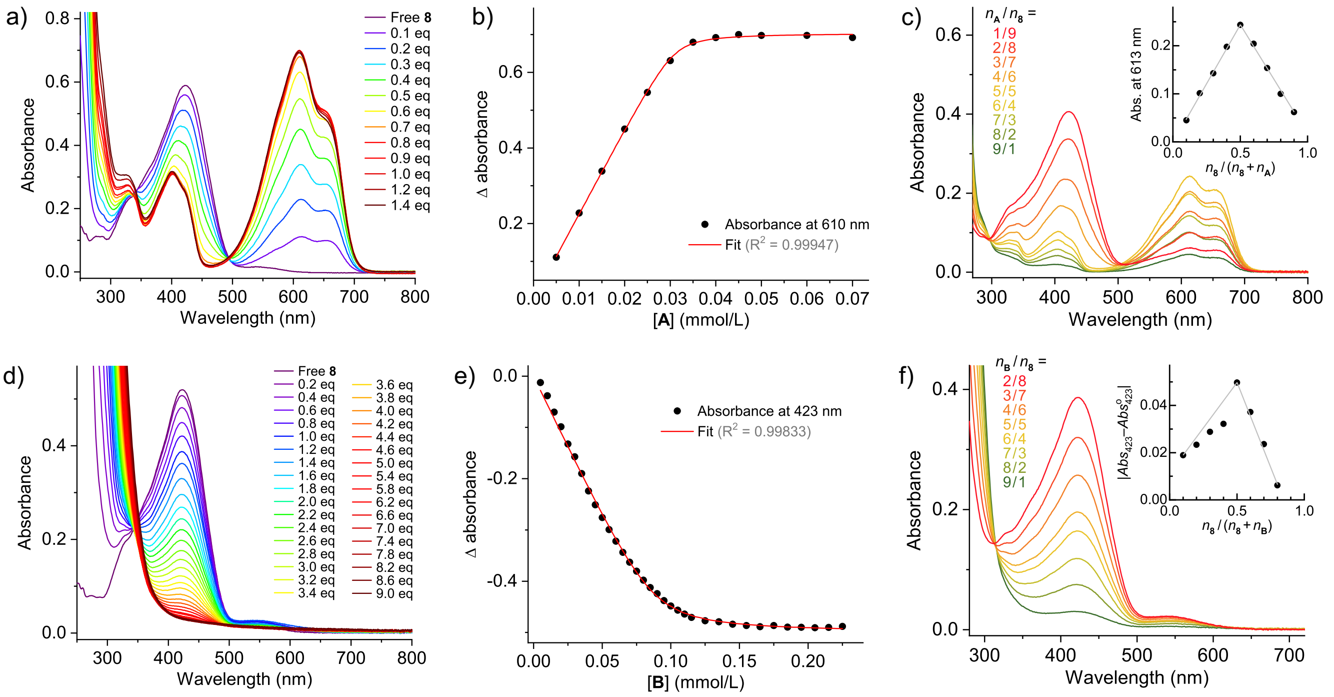

Figure S30. Studying the complexation of spiropyran **8** by cages **A** and **B**. (a) UV/vis spectra (dilution-corrected) of spiropyran **8** (initial concentration = 50 μM) in the presence of increasing amounts of cage **A**. (b) Changes in the absorbance at 610 nm (the wavelength of maximum absorption of $\mathbf{8}_{MC}$ within **A**) in the presence of increasing amounts of **A**. Nonlinear curve fitting based on a 1:1 binding model gives a K_{assoc} of 4.62(±1.11)×10^6 M^{-1} (error estimated from fitting). (c) A series of UV/vis spectra at several different molar ratios of **8** to **A**. The resulting Job's plot (inset) confirms the 1:1 stoichiometry of the inclusion complex. (d) UV/vis spectra (dilution-corrected) of **8** (initial concentration = 50 μM) in the presence of increasing amounts of cage **B**. (e) Changes in the absorbance at 423 nm (the wavelength of maximum absorption of **8**'s MCH form) in the presence of increasing amounts of **B**. Nonlinear curve fitting based on a 1:1 binding model gives a K_{assoc} of 4.10(±0.64)×10^5 M^{-1} (error estimated from fitting). (f) A series of UV/vis spectra at different molar ratios of **8** to **B**. The resulting Job's plot (inset) confirms the 1:1 stoichiometry of the inclusion complex. $|Abs_{423}–Abs^{o}_{423}|$ denotes the absolute difference between the observed absorbance at 423 nm and the absorbance at 423 nm of free **8** at the same concentration.

4. Stabilization of the MC form under acidic conditions

To determine whether cage **A** is capable of stabilizing the deprotonated MC form of the guest even under acidic conditions, we prepared a solution of $\mathbf{1}_{MC}\subset\mathbf{A}$ at the UV/vis concentration and titrated it with HNO_3, recording an absorption spectrum after the addition of each aliquot of the acid. Before the addition of the cage, the spectrum of **1** features an intense band centered at ~425 nm, characteristic of the MCH form (the pink spectrum in Figure S31). After the cage has been added, the sample exhibits strong absorption at ~590 nm (due to the encapsulated MC form; dark blue in Figure S31; at that point, pH = 4.77). Then, we added 0.2 equiv (with respect to the encapsulated MC) aliquots of HNO_3 and found that the ~590 nm peak gradually decreased at the expense of the ~425 nm band. However, even upon the addition of 2.0 equiv of HNO_3 (the red spectrum in Figure S31), the absorbance at the former wavelength is much higher than at the latter wavelength, suggesting that a vast majority of **1** remains in the MC form.

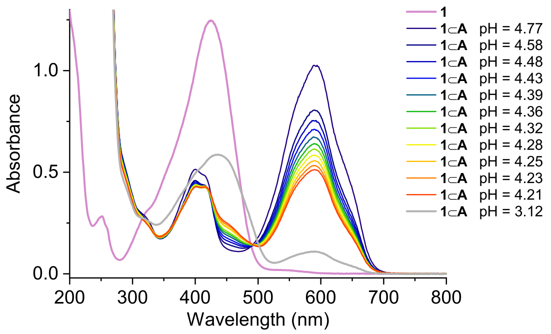

Figure S31. A series of UV/vis spectra of free (pink) and encapsulated 1, before (dark blue) and after the addition of increasing amounts of HNO_3. The following amounts of HNO_3 were added (with respect to the cage): 0.2 equiv for pH = 4.58; 0.4 equiv for pH = 4.48; 0.6 equiv for pH = 4.43; 0.8 equiv for pH = 4.39; 1.0 equiv for pH = 4.36; 1.2 equiv for pH = 4.32; 1.4 equiv for pH = 4.28; 1.6 equiv for pH = 4.25; 1.8 equiv for pH = 4.23; 2.0 equiv for pH = 4.21; and 22 equiv for pH = 3.12.

Upon the addition of an extra 20 equiv of HNO_3, the gray spectrum was obtained, whereby the blue-shifted absorption band is higher, as in the initial solution of free $\mathbf{1}_{MCH}$. However, as the series of ^{1}H NMR spectra in Figure S32 shows, this shift is (at least in part) due to the degradation of the cage and consequently the release of spiropyran to aqueous solution, where it exists in the MCH form. Specifically, we titrated a solution of cage **A** in D_2O with HCl and recorded NMR spectra at increasingly smaller pH values. The spectra recorded at pH 6.06, 5.52, and 4.13 were practically undistinguishable. However, further addition of HCl (to pH = 3.11) resulted in the appearance of new signals; at lower pH values, these signals grew and additional signals appeared. We attribute these changes to the protonation of the imidazolyl groups, which lose their ability to coordinate to Pd^{2+}, inducing disassembly of the cage. Nevertheless, these results demonstrate that cage **A** remains intact at pH = 4.11, i.e., in all the UV/vis spectra shown in Figure S31 except the last one.

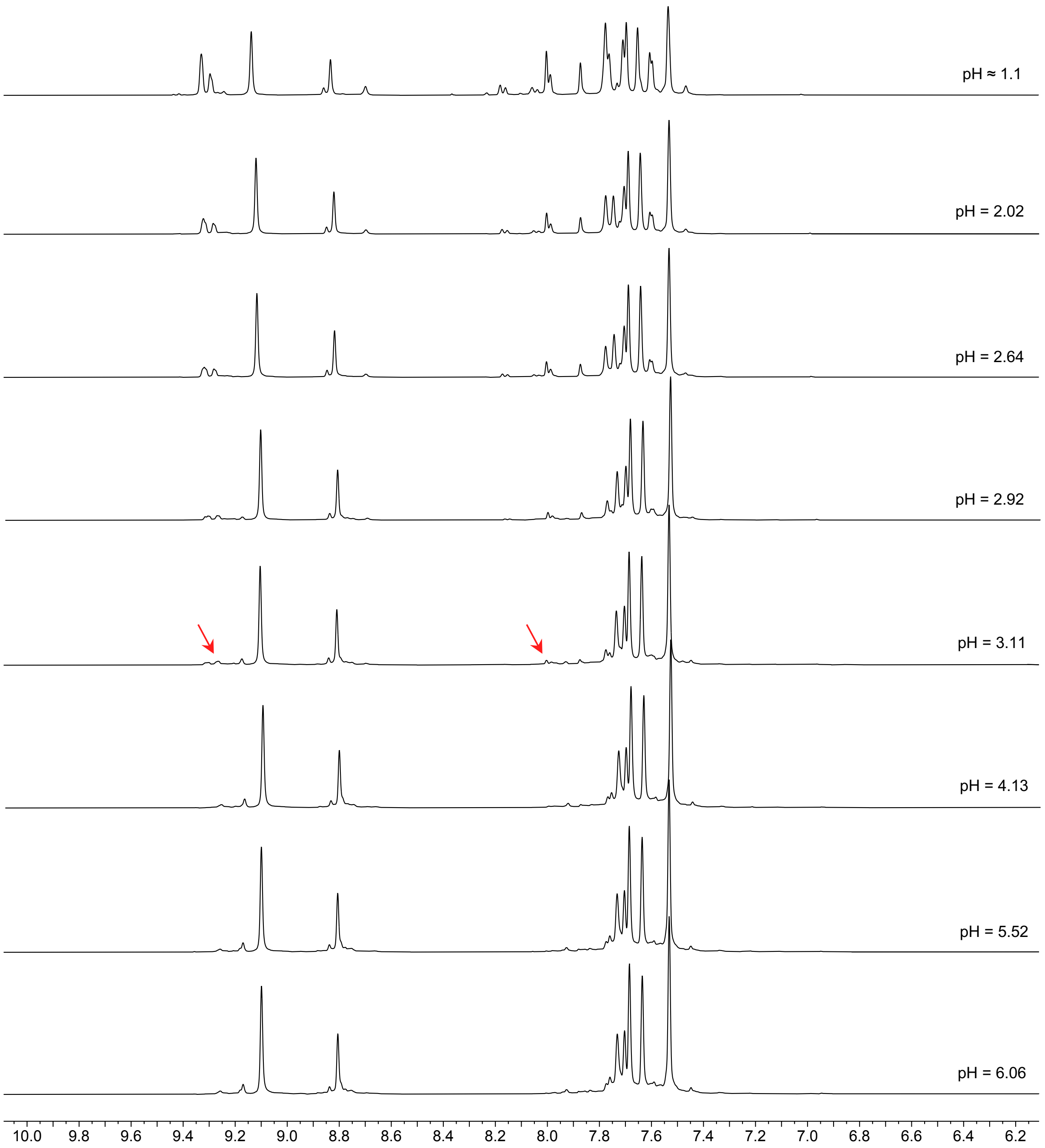

Figure S32. A series of partial ^{1}H NMR spectra of cage **A** in the presence of an increasing (bottom to top) amount of HCl (500 MHz, D_2O, 298 K). The red arrows denote new signals that appeared at upon acidifying the solution from pH 4.13 to 3.11.

5. Cage **A** protects the MC form of spiropyrans against hydrolysis

To study the stabilization of the MC form by cage **A**, we prepared all eight inclusion complexes (20 μM solutions in water), treated them with 1 equiv of KOH, and monitored the changes in the UV/vis spectra (the pH of the resulting solutions was well below 10, which is the pH above which **A** starts decomposing). As control experiments, we solubilized **1–8** in MeCN (20 μM) and monitored their UV/vis spectra after 1 equiv of KOH (concentrated solution in water) was added (the final solvent composition = MeCN with 2 vol% H_2O). The results of these experiments are shown in Figures S33–S40.

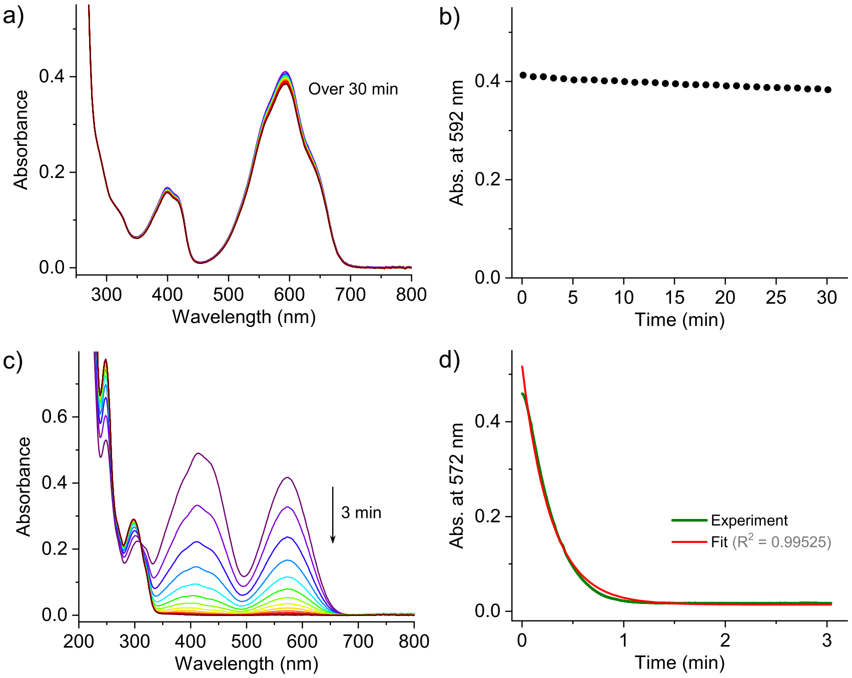

Figure S33. Comparison of the stability of free and encapsulated $\mathbf{1}_{MC}$. (a) A series of UV/vis spectra of $\mathbf{1}_{MC}\subset\mathbf{A}$ recorded over a period of 30 min. (b) Change in the absorbance at 592 nm of $\mathbf{1}_{MC}\subset\mathbf{A}$ over 30 min. (c) A series of UV/vis spectra of free $\mathbf{1}_{MC}$ recorded over a period of 30 min (one spectrum every 6 s). (d) Change in $\mathbf{1}_{MC}$'s absorbance at 572 nm over 3 min; fitting to a first-order rate equation gives a rate constant of $k = 3.55\ \mathrm{min}^{-1}$.

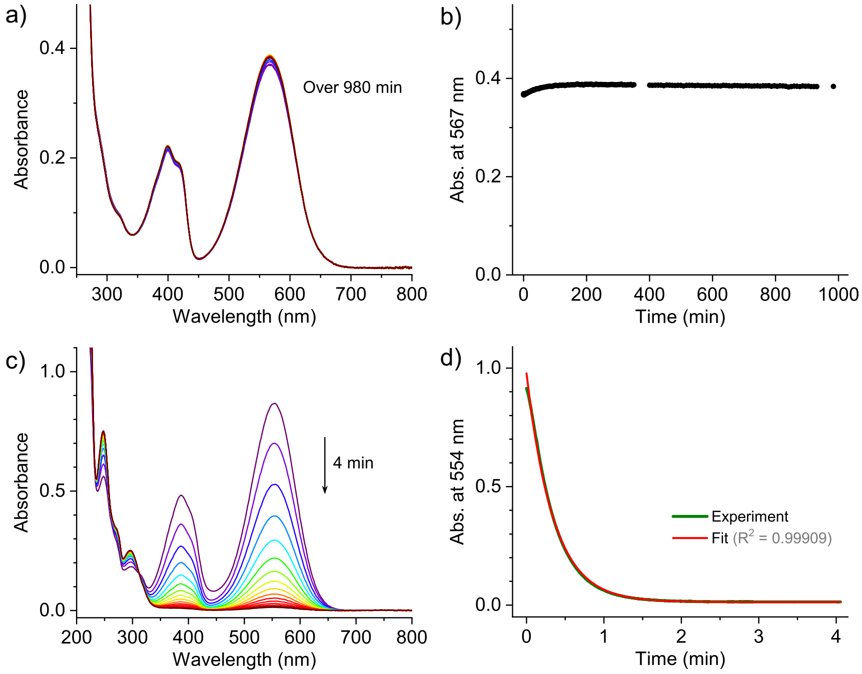

Figure S34. Comparison of the stability of free and encapsulated $\mathbf{2}_{\mathrm{MC}}$. (a) A series of UV/vis spectra of $\mathbf{2}_{\mathrm{MC}}\subset\mathbf{A}$ recorded over a period of 980 min. (b) Change in the absorbance at 567 nm of the $\mathbf{2}_{\mathrm{MC}}\subset\mathbf{A}$ solution over 30 min. (c) A series of UV/vis spectra of free $\mathbf{2}_{\mathrm{MC}}$ recorded over a period of 3 min (one spectrum every 6 s). (d) Change in the absorbance at 554 nm of the $\mathbf{2}_{\mathrm{MC}}$ solution over 4 min; fitting to a first-order rate equation gives a rate constant of $k = 2.91\ \mathrm{min}^{-1}$.

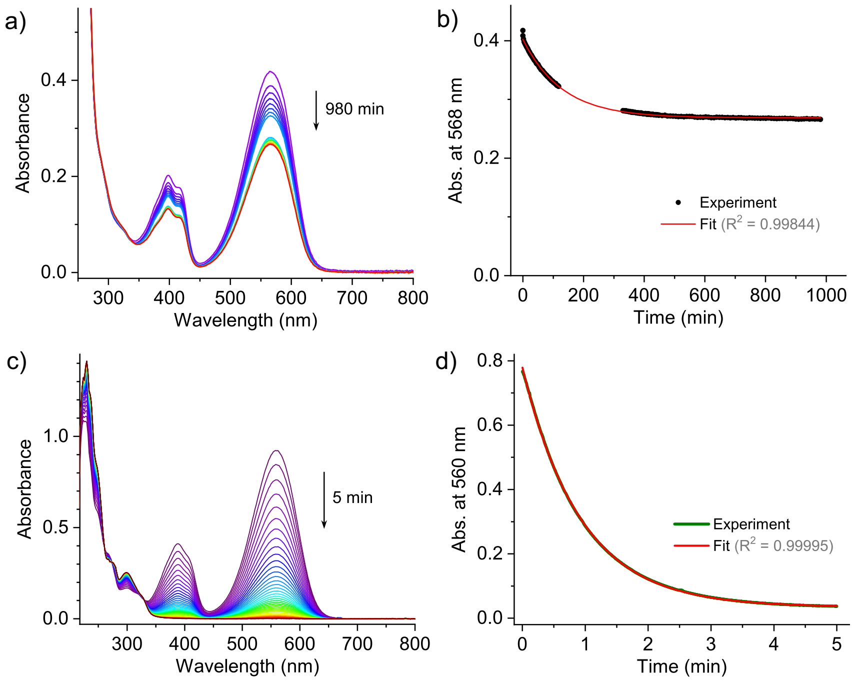

Figure S35. Comparison of the stability of free and encapsulated $\mathbf{3}_{\mathrm{MC}}$. (a) A series of UV/vis spectra of $\mathbf{3}_{\mathrm{MC}}\subset\mathbf{A}$ recorded over a period of 980 min. (b) Change in the absorbance at 568 nm of the $\mathbf{3}_{\mathrm{MC}}\subset\mathbf{A}$ solution over 980 min; fitting to a first-order rate equation gives a rate constant of $k = 0.0078\ \mathrm{min}^{-1}$. (c) A series of UV/vis spectra of free $\mathbf{3}_{\mathrm{MC}}$ recorded over a period of 3 min (one spectrum every 6 s). (d) Change in the absorbance at 560 nm of the $\mathbf{3}_{\mathrm{MC}}$ solution over 3 min; fitting to a first-order rate equation gives a rate constant of $k = 1.08\ \mathrm{min}^{-1}$.

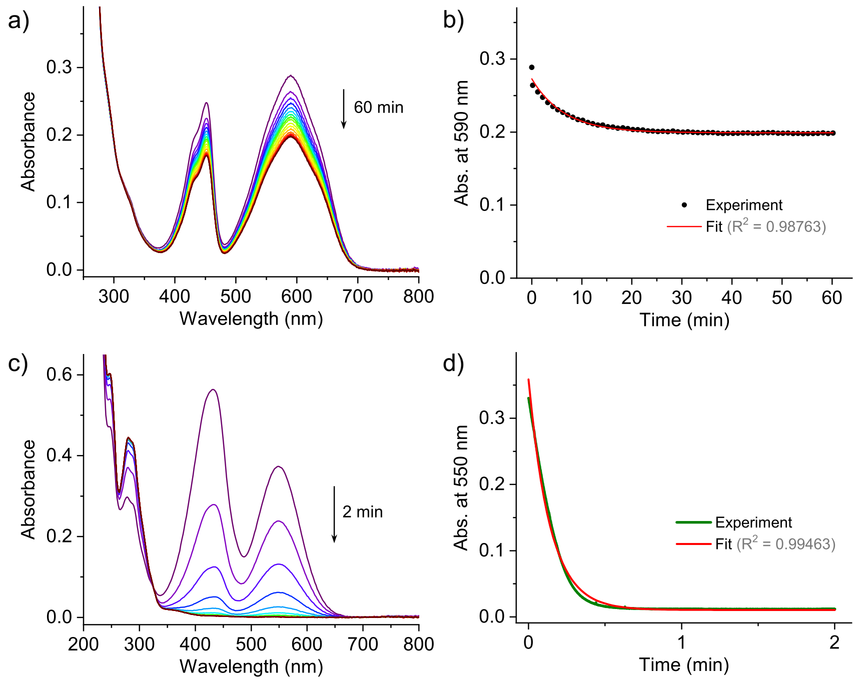

Figure S36. Comparison of the stability of free and encapsulated **4**$_{MC}$. (a) A series of UV/vis spectra of **4**$_{MC}$⊂**A** recorded over a period of 60 min. (b) Change in the absorbance at 590 nm of the **4**$_{MC}$⊂**A** solution over 60 min; fitting to a first-order rate equation gives a rate constant of k = 0.15 min^{-1}. (c) A series of UV/vis spectra of free **4**$_{MC}$ recorded over a period of 2 min (one spectrum every 6 s). (d) Change in the absorbance at 550 nm of the **4**$_{MC}$ solution over 2 min; fitting to a first-order rate equation gives a rate constant of k = 7.16 min^{-1}.

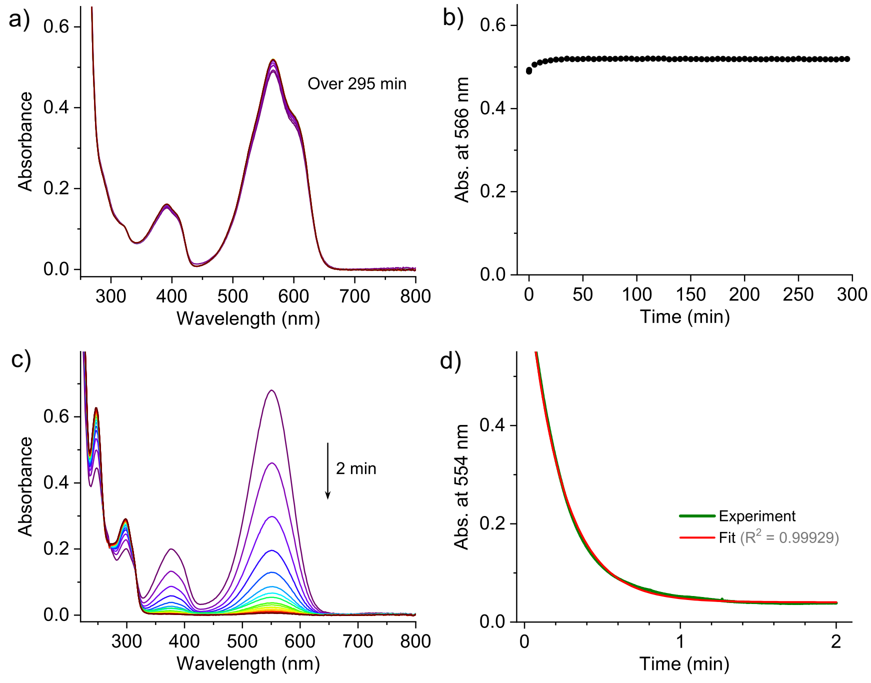

Figure S37. Comparison of the stability of free and encapsulated **5**$_{MC}$. (a) A series of UV/vis spectra of **5**$_{MC}$⊂**A** recorded over a period of 295 min. (b) Change in the absorbance at 566 nm of the **5**$_{MC}$⊂**A** solution over 295 min. (c) A series of UV/vis spectra of free **5**$_{MC}$ recorded over a period of 2 min (one spectrum every 6 s). (d) Change in the absorbance at 554 nm of the **5**$_{MC}$ solution over 2 min; fitting to a first-order rate equation gives a rate constant of k = 4.46 min^{-1}.

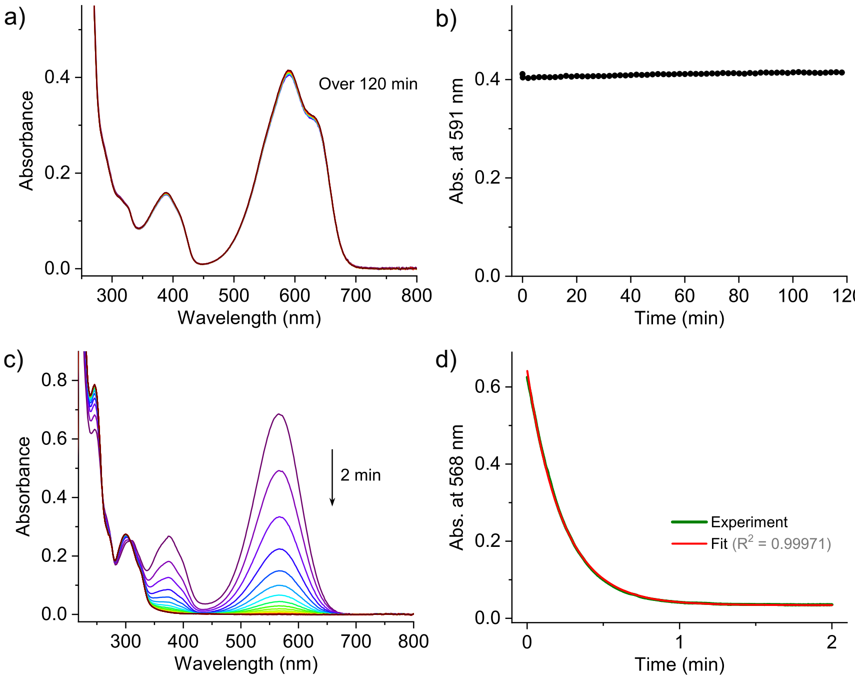

Figure S38. Comparison of the stability of free and encapsulated **6**$_{MC}$. (a) A series of UV/vis spectra of **6**$_{MC}$⊂**A** recorded over a period of 120 min. (b) Change in the absorbance at 591 nm of the **6**$_{MC}$⊂**A** solution over 120 min. (c) A series of UV/vis spectra of free **6**$_{MC}$ recorded over a period of 2 min (one spectrum every 6 s). (d) Change in the absorbance at 568 nm of the **6**$_{MC}$ solution over 2 min; fitting to a first-order rate equation gives a rate constant of $k = 4.38$ min^{-1}.

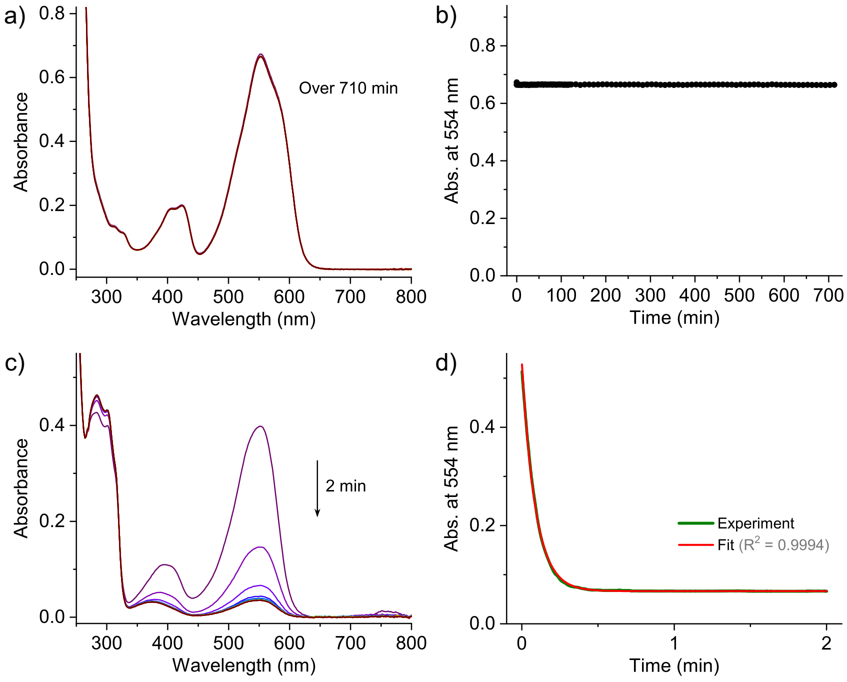

Figure S39. Comparison of the stability of free and encapsulated **7**$_{MC}$. (a) A series of UV/vis spectra of **7**$_{MC}$⊂**A** recorded over a period of 710 min. (b) Change in the absorbance at 554 nm of the **7**$_{MC}$⊂**A** solution over 710 min. (c) A series of UV/vis spectra of free **7**$_{MC}$ recorded over a period of 2 min (one spectrum every 6 s). (d) Change in the absorbance at 554 nm of the **7**$_{MC}$ solution over 2 min; fitting to a first-order rate equation gives a rate constant of $k = 11.3$ min^{-1}.

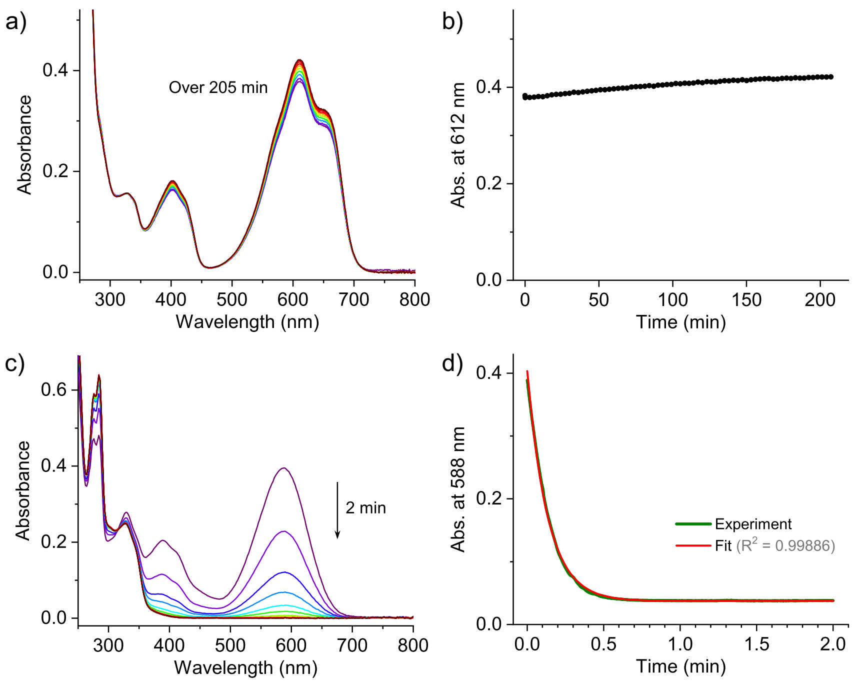

Figure S40. Comparison of the stability of free and encapsulated **8**$_{MC}$. (a) A series of UV/vis spectra of **8**$_{MC}$⊂**A** recorded over a period of 205 min. (b) Change in the absorbance at 612 nm of the **8**$_{MC}$⊂**A** solution over 205 min. (c) A series of UV/vis spectra of free **8**$_{MC}$ recorded over a period of 2 min (one spectrum every 6 s). (d) Change in the absorbance at 588 nm of the **8**$_{MC}$ solution over 2 min; fitting to a first-order rate equation gives a rate constant of $k = 7.50$ min^{-1}.

6. NMR spectra of encapsulated spiropyrans

Samples for NMR spectroscopy were prepared by stirring concentrated solutions of cages **A** and **B** in D_2O above solid spiropyrans **1**–**8** (which, in the free form, have poor solubilities in water). After stirring overnight, the suspensions were filtered through a syringe filter to remove any undissolved solids and the resulting solutions were analyzed by NMR spectroscopy. The ^{1}H NMR spectra of inclusion complexes **1**⊂**A** through **8**⊂**A** are shown in Figures S41–48; the spectra of **1**⊂**B** through **8**⊂**B** s are shown in Figures S49–56.

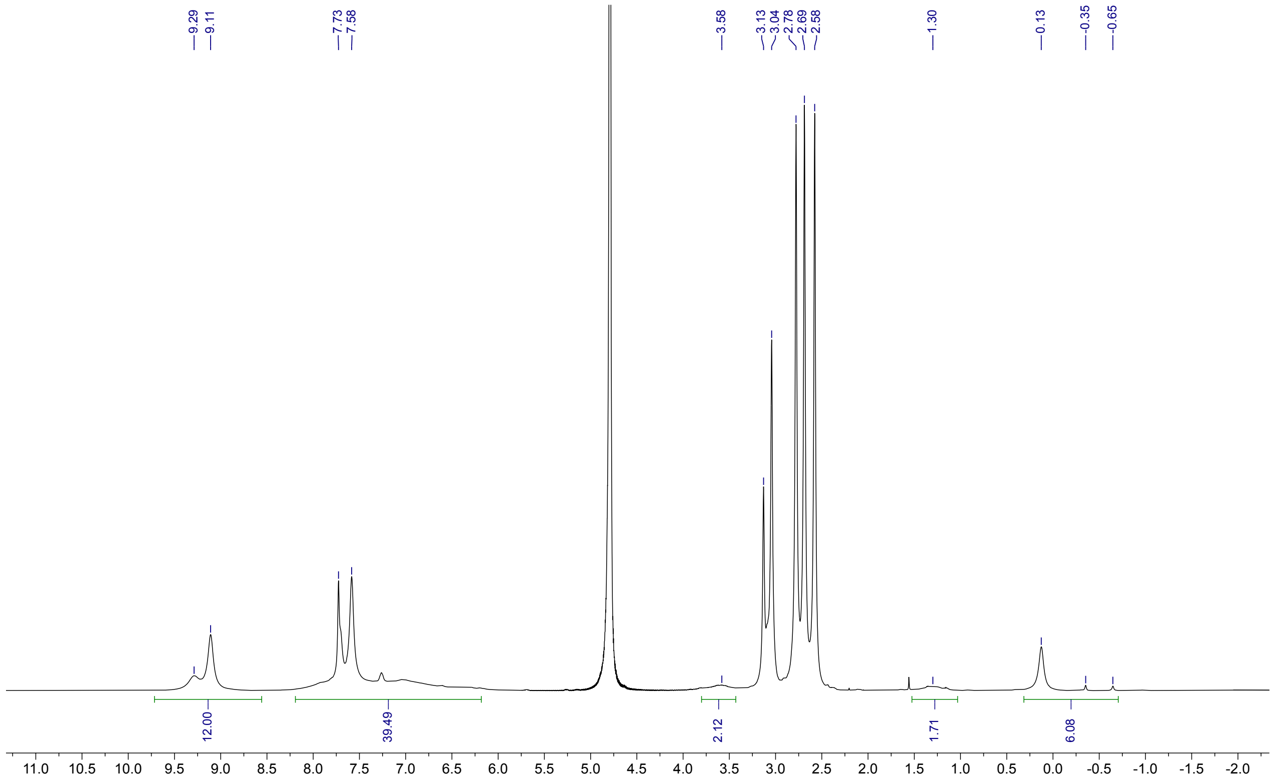

Figure S41. ^{1}H NMR spectrum of **1⊂A** (500 MHz, D_2O, 298 K).

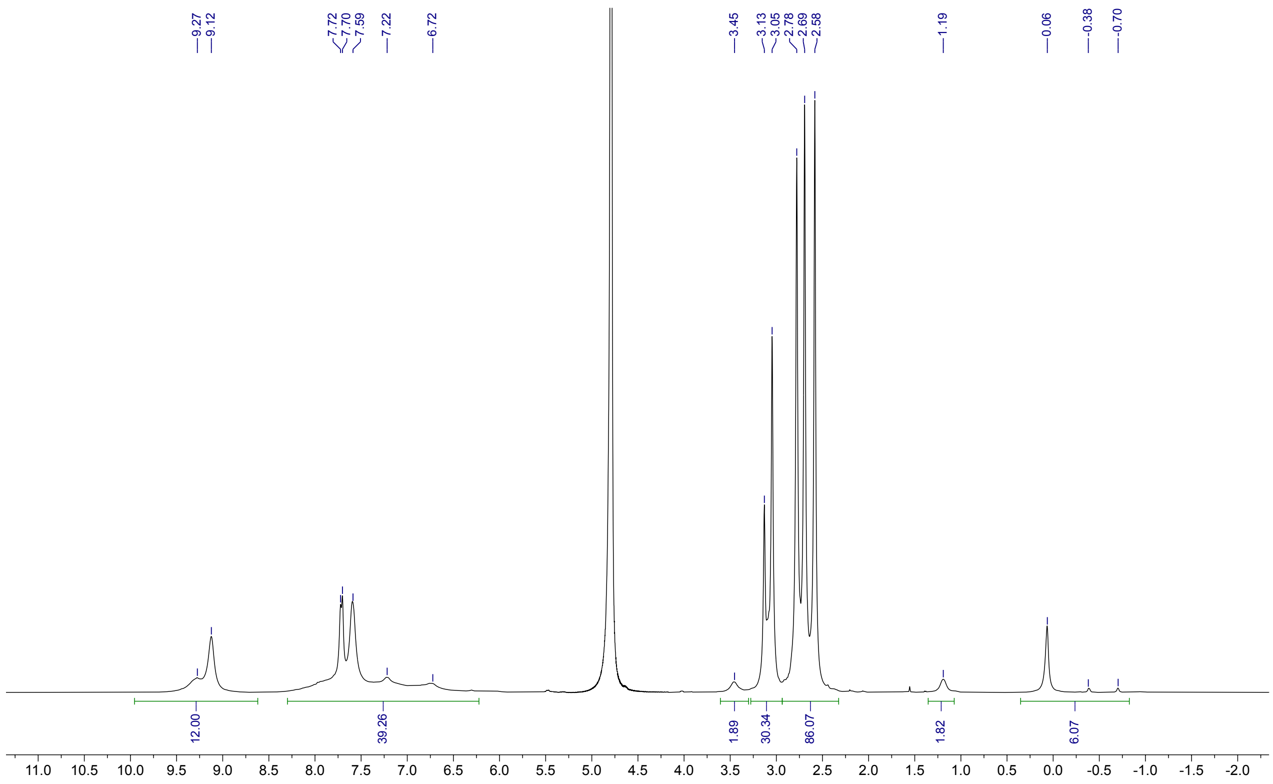

Figure S42. ^{1}H NMR spectrum of **2⊂A** (500 MHz, D_2O, 298 K).

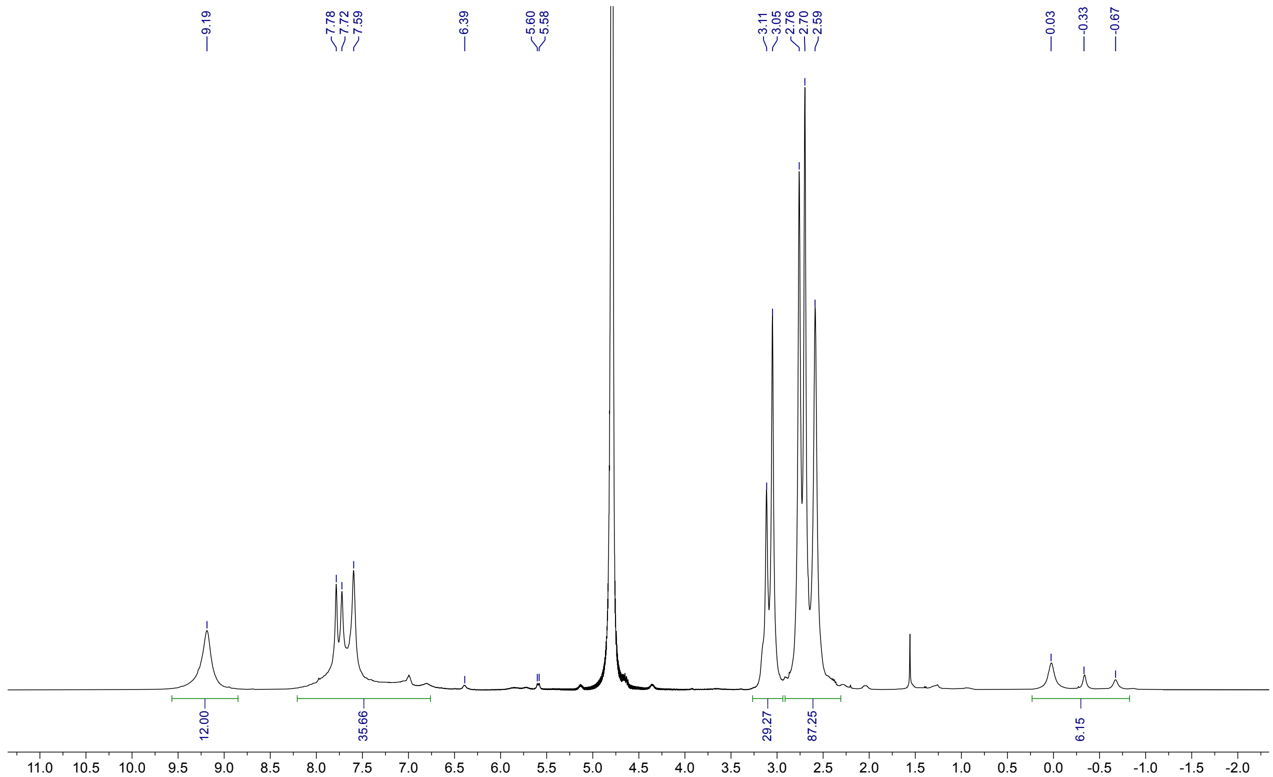

Figure S43. ^{1}H NMR spectrum of **3⊂A** (500 MHz, D_2O, 298 K).

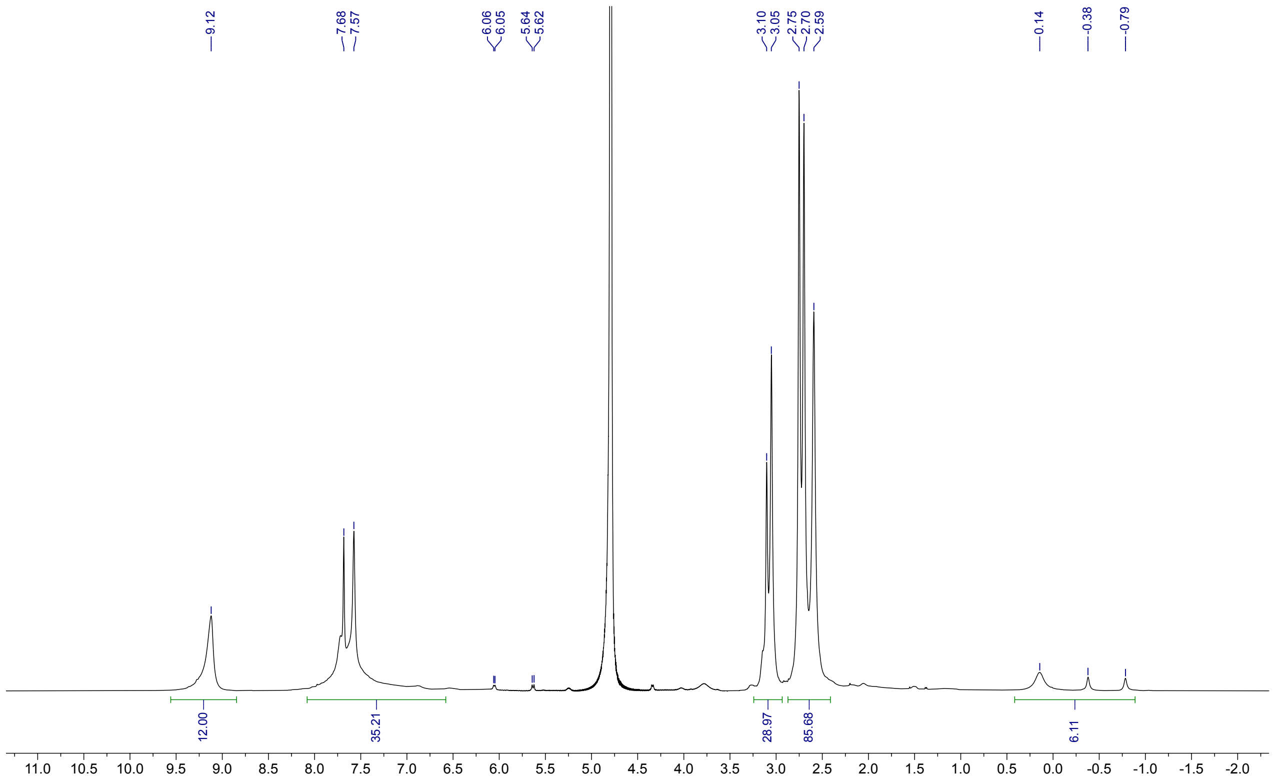

Figure S44. ^{1}H NMR spectrum of **4⊂A** (500 MHz, D_2O, 298 K).

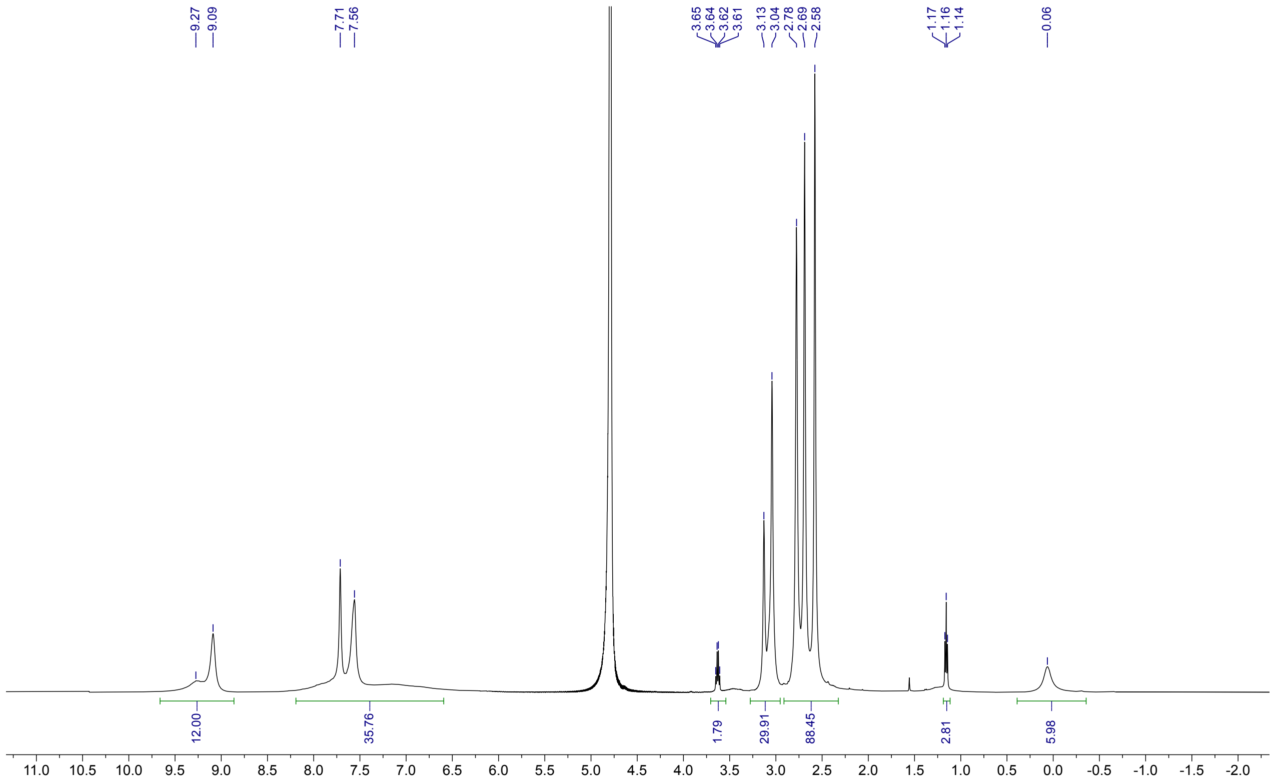

Figure S45. ^{1}H NMR spectrum of **5⊂A** (500 MHz, D_2O, 298 K).

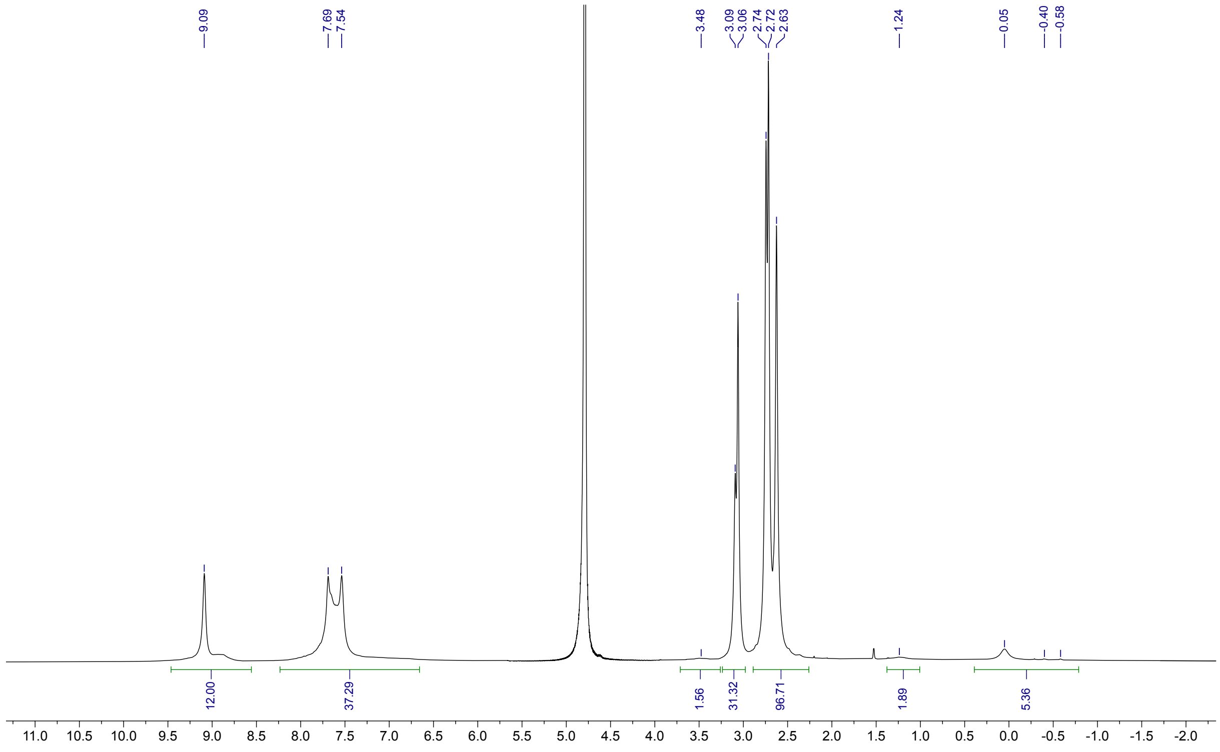

Figure S46. ^{1}H NMR spectrum of **6⊂A** (500 MHz, D_2O, 298 K).

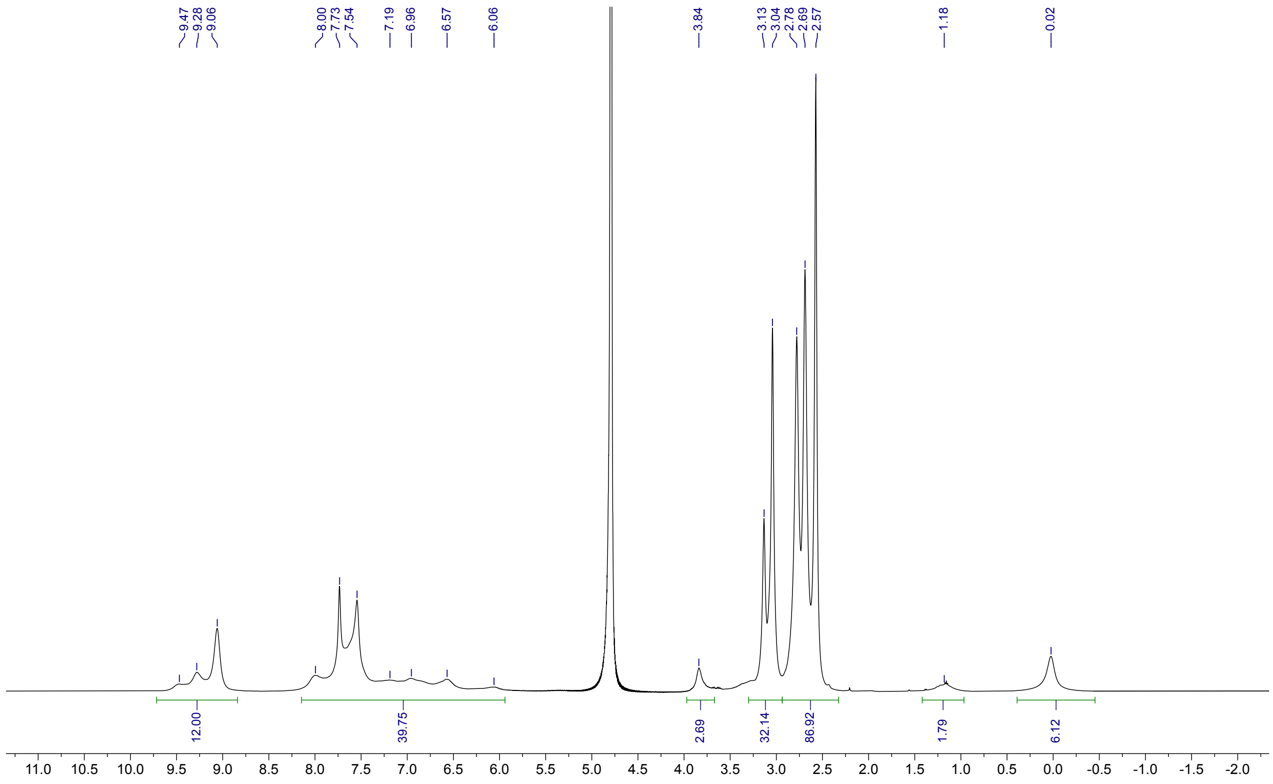

Figure S47. ^{1}H NMR spectrum of **7⊂A** (500 MHz, D_2O, 298 K).

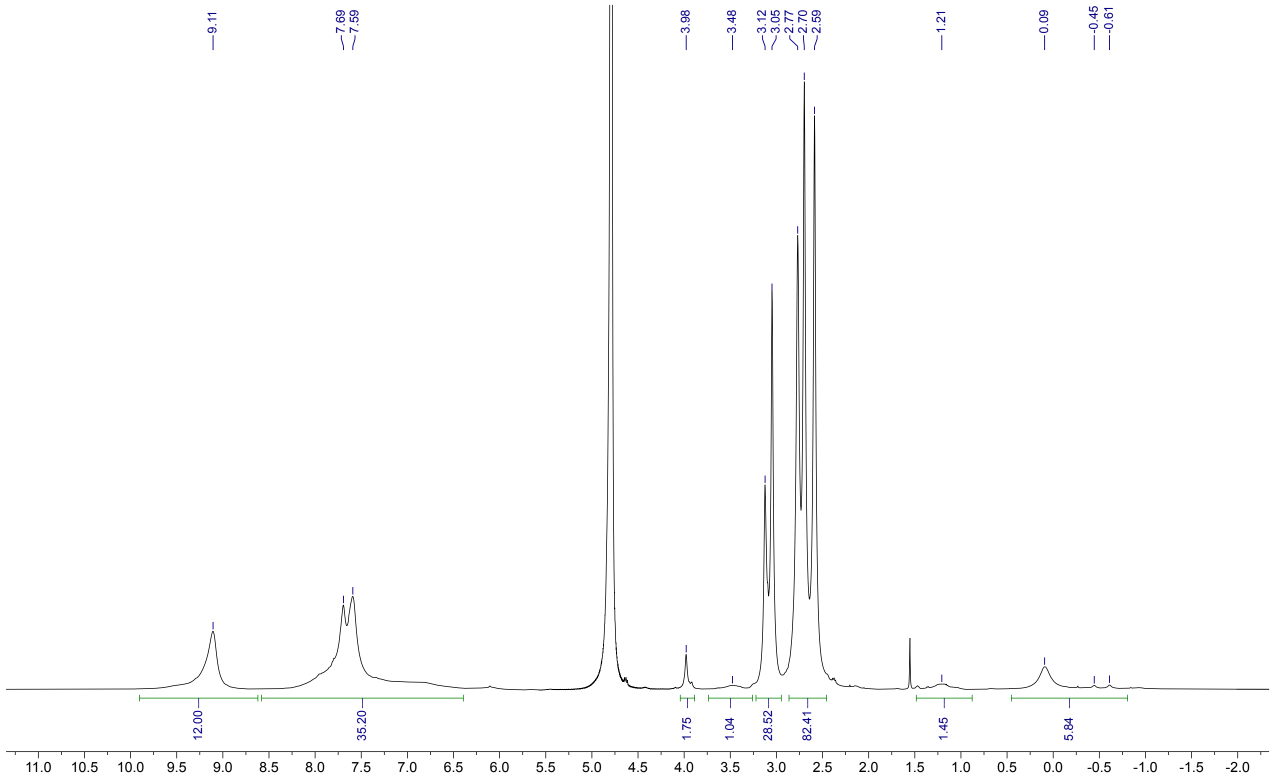

Figure S48. ^{1}H NMR spectrum of **8⊂A** (500 MHz, D_2O, 298 K).

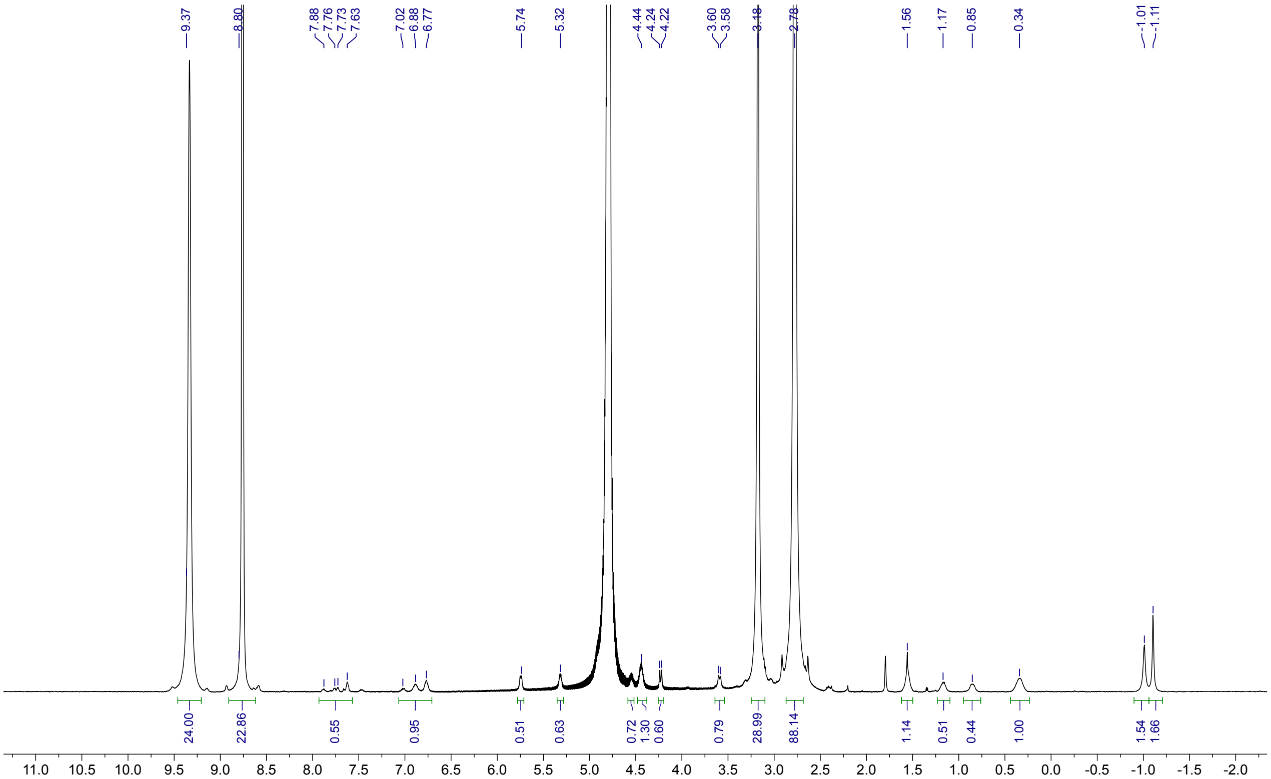

Figure S49. ^{1}H NMR spectrum of **1⊂B** (500 MHz, D_2O, 298 K).

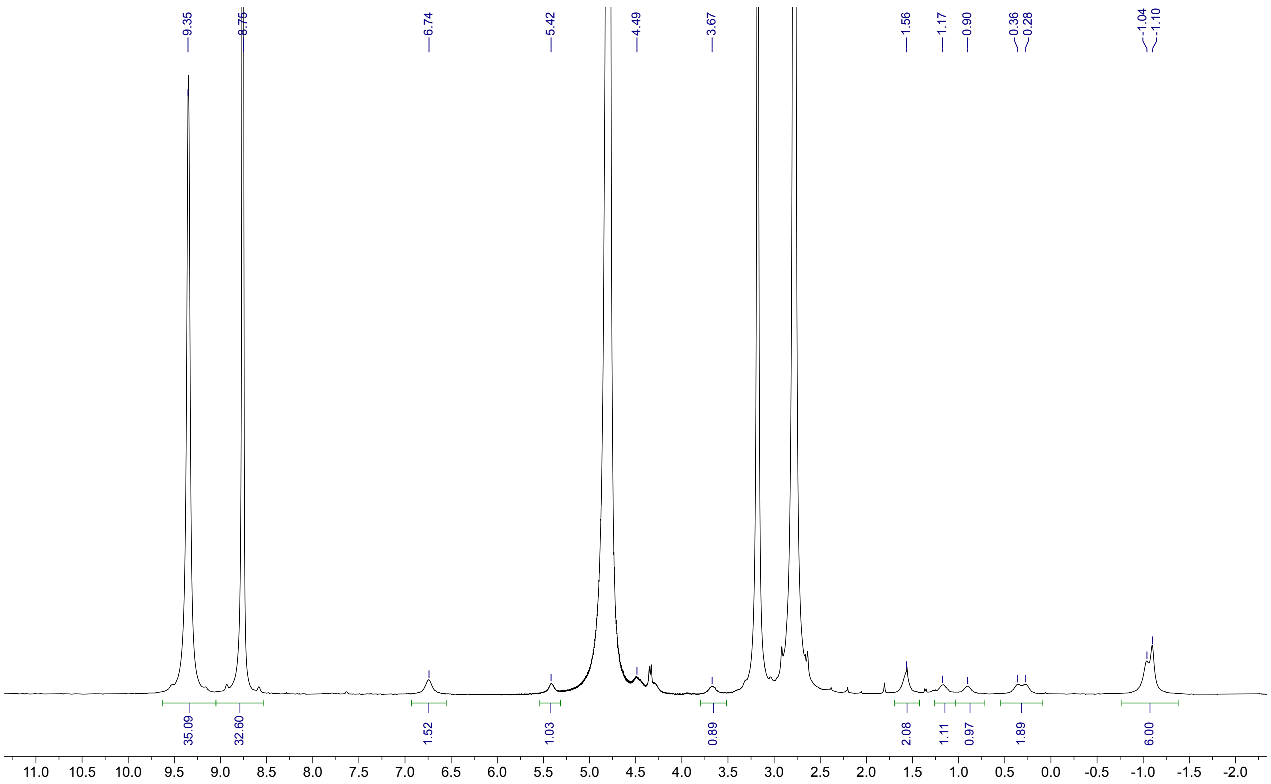

Figure S50. ^{1}H NMR spectrum of **2⊂B** (500 MHz, D_2O, 298 K).

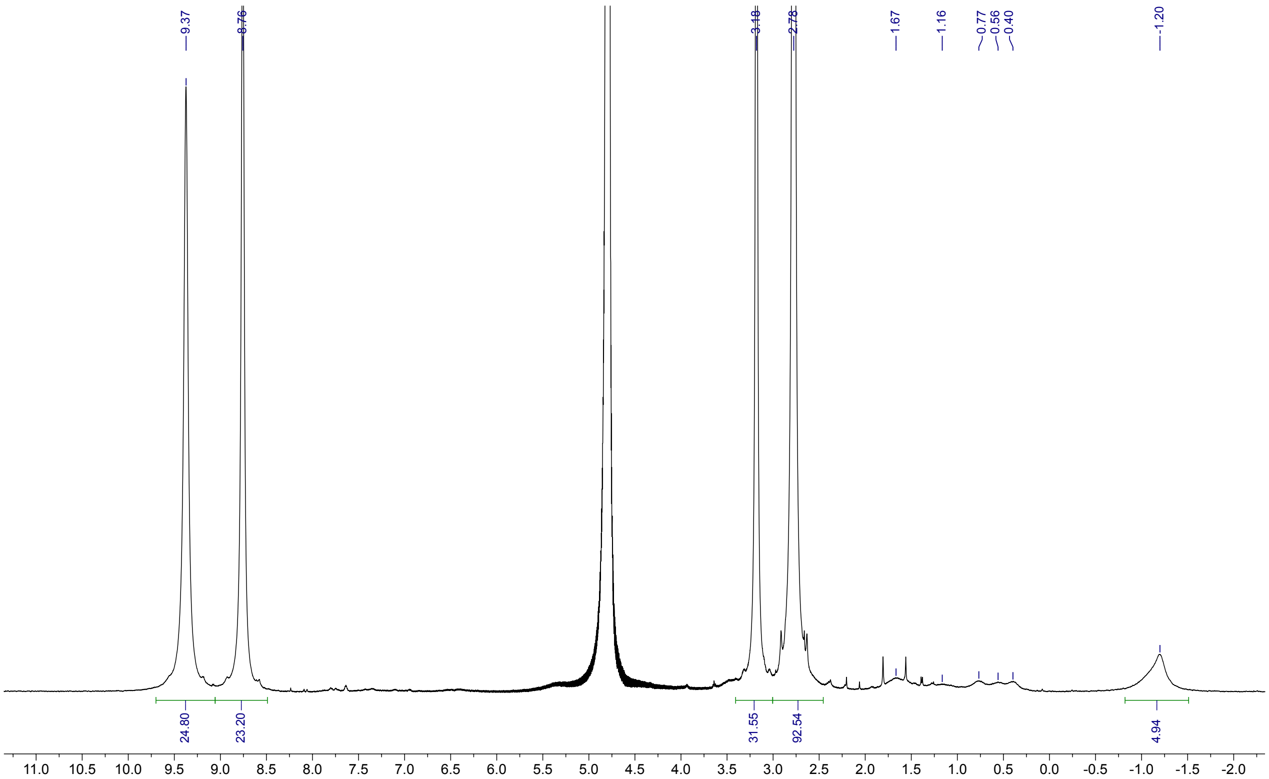

Figure S51. ^{1}H NMR spectrum of **3⊂B** (500 MHz, D_2O, 298 K).

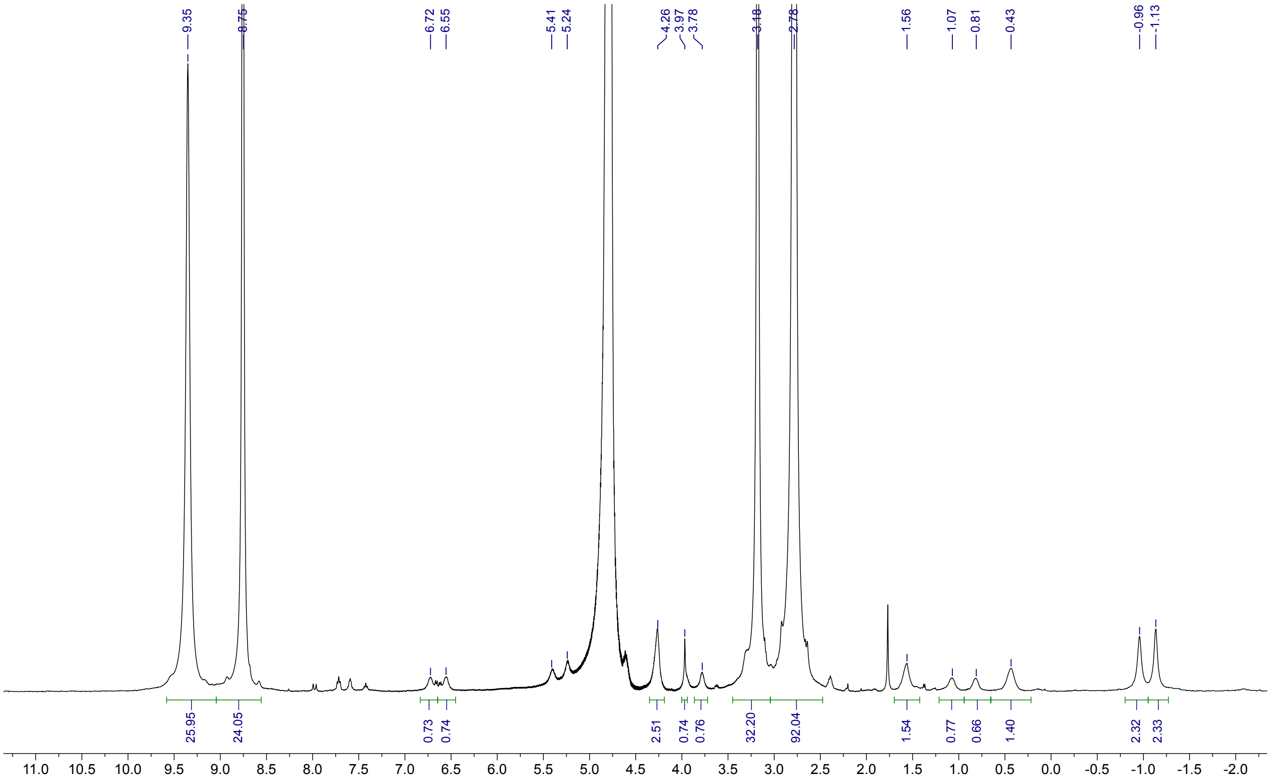

Figure S52. ^{1}H NMR spectrum of **4⊂B** (500 MHz, D_2O, 298 K).

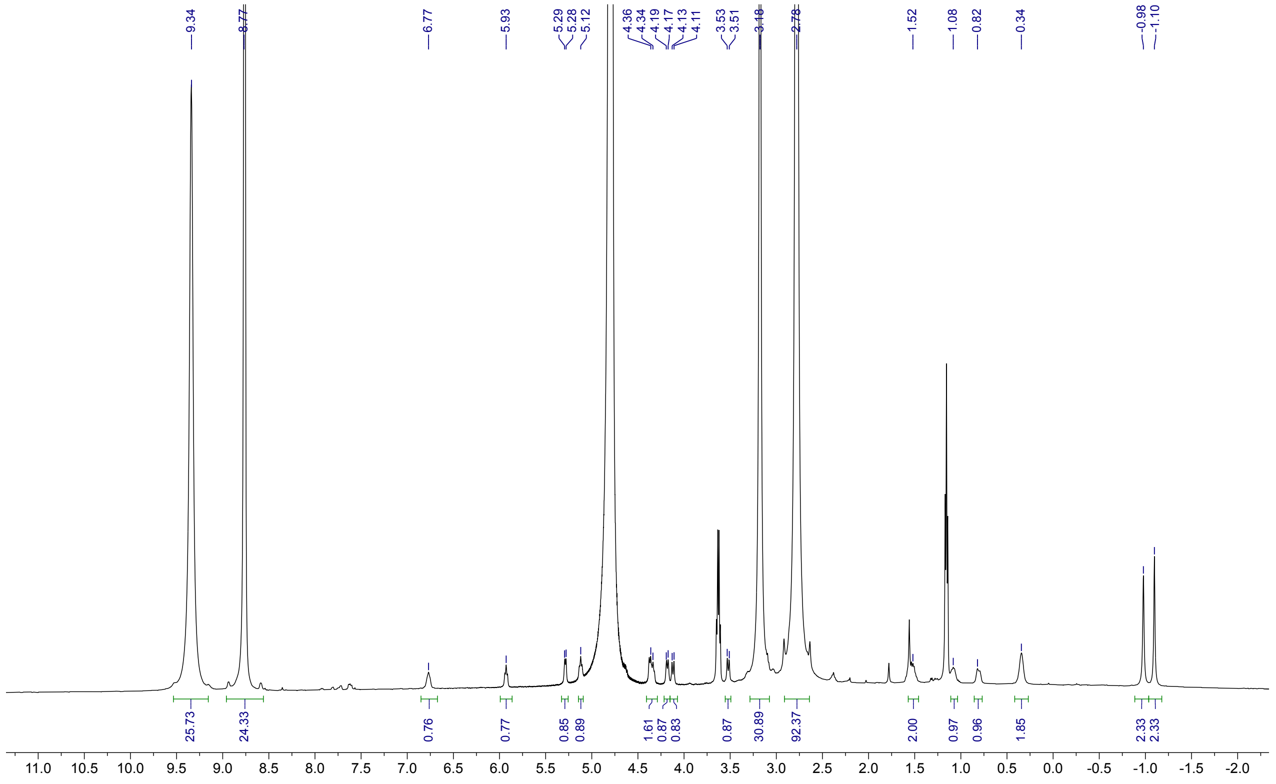

Figure S53. ^{1}H NMR spectrum of **5⊂B** (500 MHz, D_2O, 298 K).

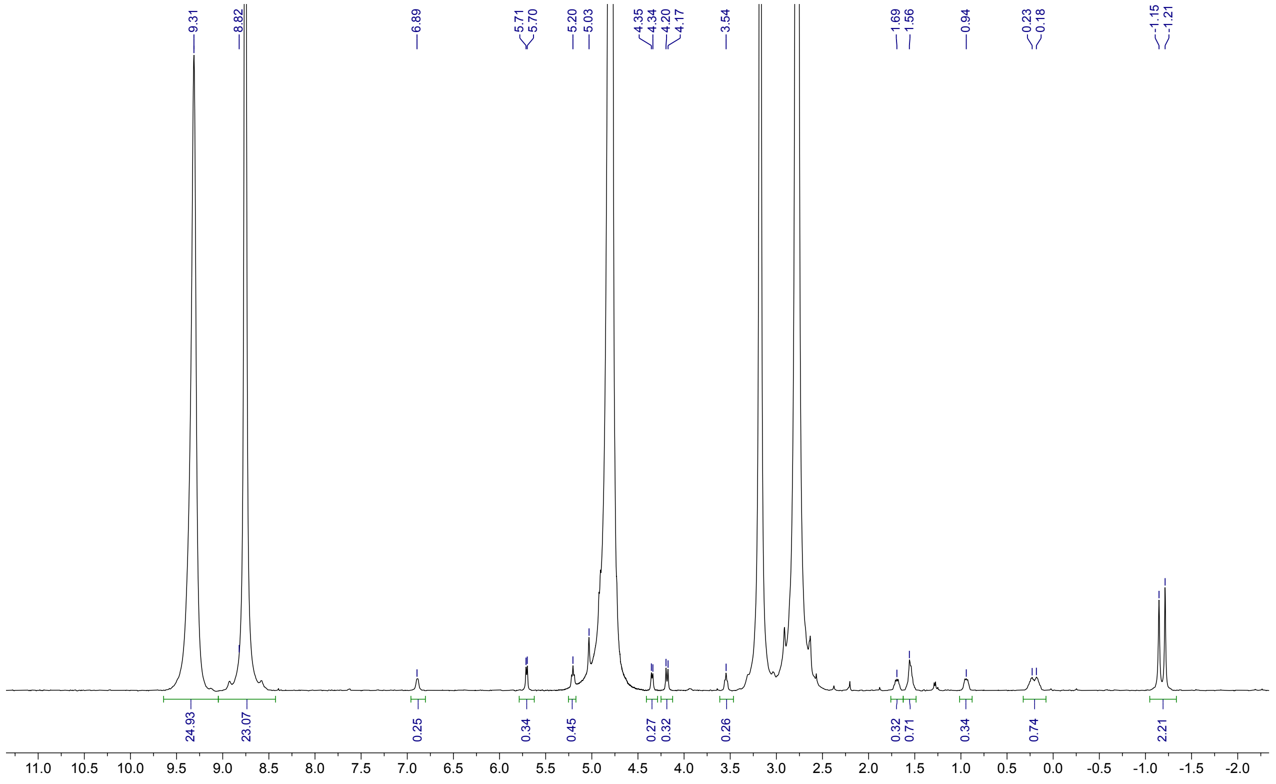

Figure S54. ^{1}H NMR spectrum of **6⊂B** (500 MHz, D_2O, 298 K).

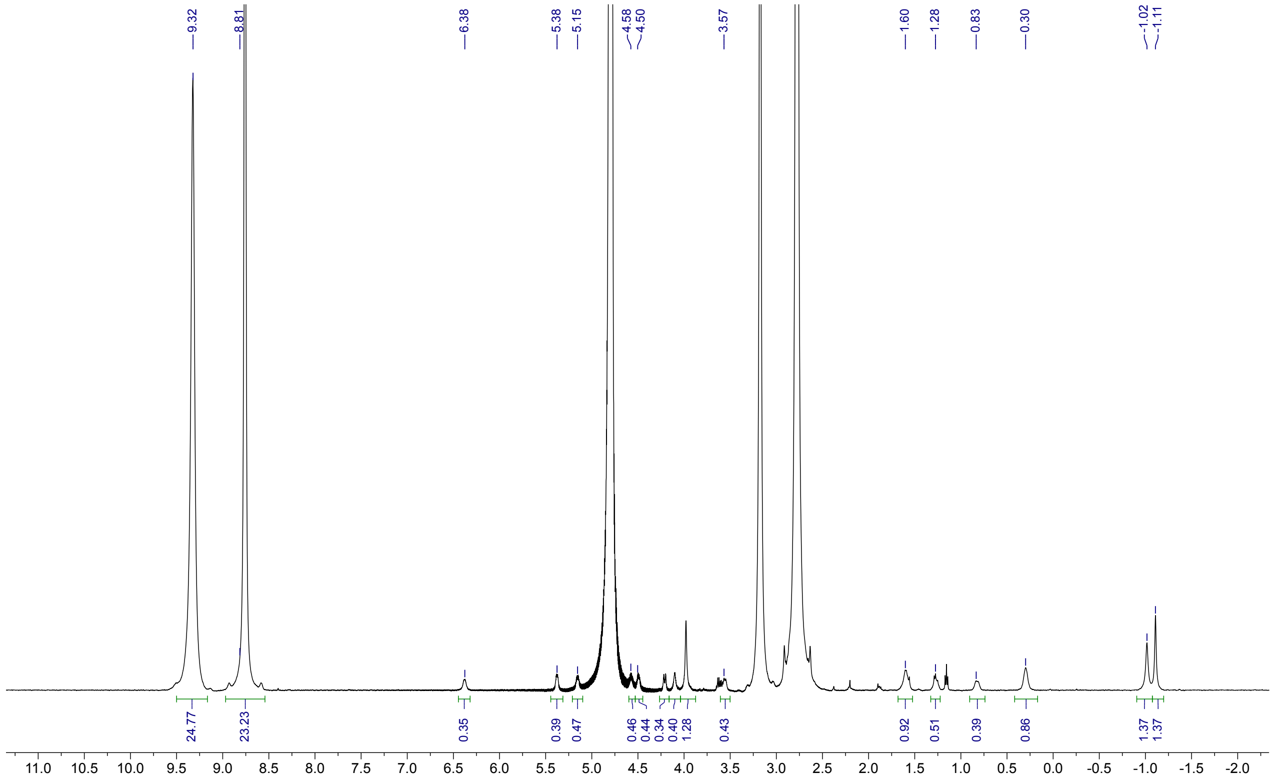

Figure S55. ^{1}H NMR spectrum of **7⊂B** (500 MHz, D_2O, 298 K).

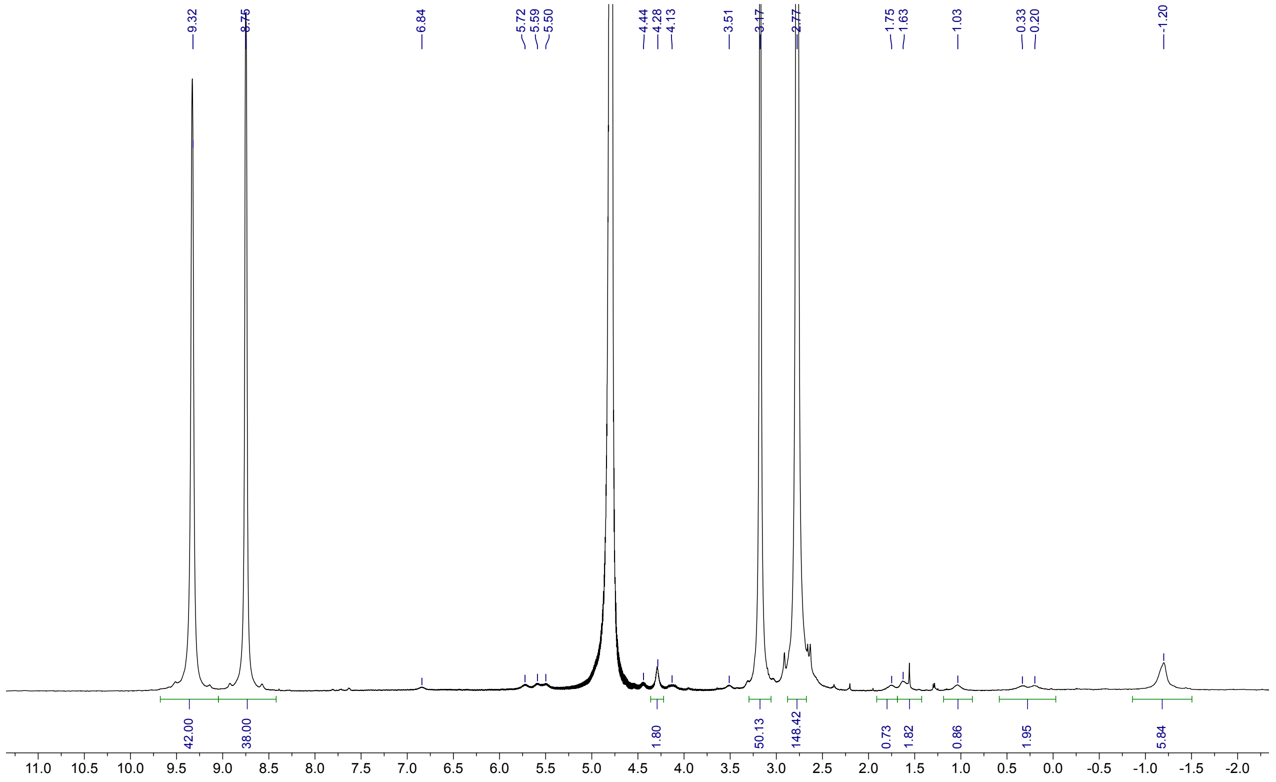

Figure S56. ^{1}H NMR spectrum of **8⊂B** (500 MHz, D_2O, 298 K).

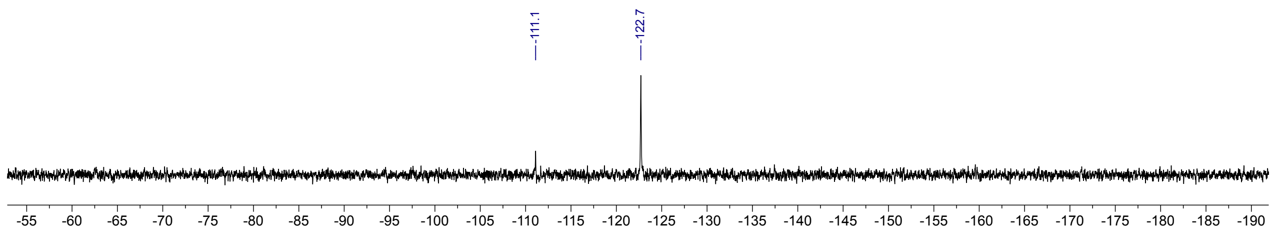

Figure S57. ^{19}F NMR spectrum of **2⊂B** in the presence of excess **2** (470 MHz, D_2O, 298 K). The signals at –122.7 ppm and –111.1 ppm originate from the encapsulated and free **2**, respectively.

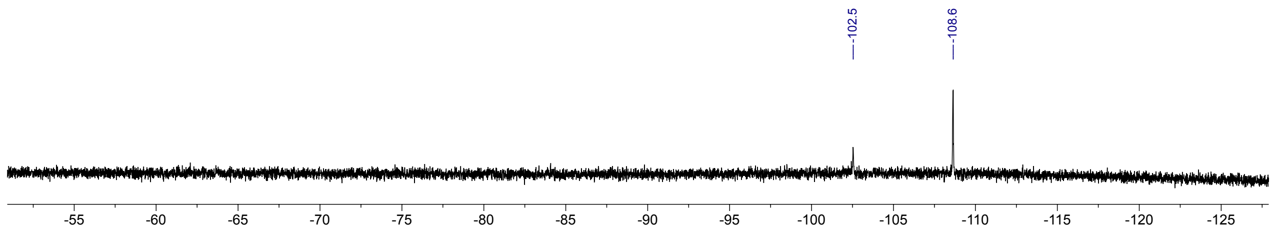

Figure S58. ^{19}F NMR spectrum of **5⊂B** in the presence of excess **5** (376 MHz, D_2O, 298 K). The signals at –108.6 ppm and –102.5 ppm originate from the encapsulated and free **5**, respectively.

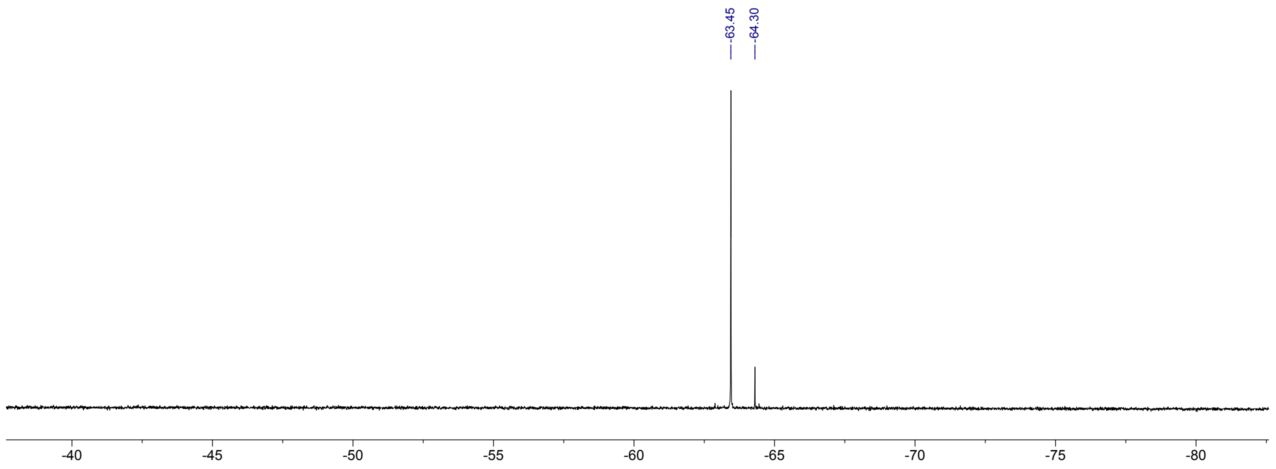

Figure S59. ^{19}F NMR spectrum of **6⊂B** in the presence of excess **6** (376 MHz, D_2O, 298 K). The signals at –63.4 ppm and –64.3 ppm originate from the encapsulated and free **6**, respectively.

7. X-ray data collection and structure refinement

Single crystals of inclusion complexes **2⊂A** and **5⊂A** were obtained by slow evaporation of water from the concentrated aqueous solution of the respective complex (typically, the crystals appeared within 2–3 weeks at room temperature). For **2⊂A**, the diffraction data were collected on a Rigaku Synergy diffractometer using Mo-Kα radiation (0.71073 Å) and a Dectris Pilatus 3R 300K CdTe detector. For **5⊂A**, the diffraction data were collected on a Rigaku XtaLAB Synergy R rotating anode system diffractometer using Cu-Kα radiation (1.54184 Å) and a HyPix-Arc 150 detector. The collected data were processed with the CrysAlis^{PRO} software package. The structures were solved using SHELXT[12] and refined by full-matrix least-squares methods on F^2 with SHELXL[13] with the aid of OLEX2.[14] All non-hydrogen atoms were further refined by SHELXL with anisotropic displacement coefficients; the positions of hydrogen atoms were calculated and refined in a riding mode. The contribution of disordered water molecules from the electron density map was eliminated using the SQUEEZE procedure.[15] Crystallographic data and refinement parameters are summarized in Table S1.

	**2⊂A**	**5⊂A**
**CCDC deposition number**	2194230	2194231
**Empirical formula**	$C_{117}H_{165}FN_{45}O_{30}Pd_6S$	$C_{232}H_{324}F_2N_{96}O_{75}Pd_{12}S_2$
**Formula weight**	3378.30	7034.00
**Crystal system, space group**	Triclinic, $P\bar{1}$	Orthorhombic, *Pnnm*
**Crystal size (mm)**	0.567 × 0.389 × 0.278	0.077 × 0.057 × 0.054
**Crystal color and shape**	Orange block	Red prism
**Temperature (K)**	100(10)	120(10)
**X-ray wavelength (Å)**	0.71073	1.54184
**a, b, c (Å)**	17.8297(2), 24.2936(4), 24.7553(4)	29.7896(2), 27.7843(2), 23.6881(2)
**α, β, γ (°)**	74.4380(10), 84.2830(10), 82.4890(10)	90, 90, 90
**Volume (Å^3)**	10217.6(3)	19606.2(3)
***F*(000)**	4431	9469
**θ range for data collection**	1.669–31.703	3.967–79.616
**Limiting indices**	$-21 \leq h \leq 21$; $-28 \leq k \leq 28$; $-29 \leq l \leq 29$	$-37 \leq h \leq 35$; $-32 \leq k \leq 35$; $-29 \leq l \leq 30$
**Calculated density (g·cm^{-3})**	1.098	1.191
Z	2	2
**Absorption coefficient (mm^{-1})**	0.588	5.037
**Reflections collected / unique (R_{int})**	171010 / 36066 (0.0403)	161557 / 21779 (0.0396)
**Completeness**	99.9%	98.9%
**Largest electron/hole density (e·Å^{-3})**	2.003 / –1.315	1.989 / –1.168
**Data / restraints / parameters**	36066 / 489 / 2249	21779 / 143 / 1158
**Goodness-of-fit on F^2**	1.036	1.059
**Final *R* indices [I>2σ(I)]**	R_1 = 0.0732, wR_2 = 0.1869	R_1 = 0.0707, wR_2 = 0.2079
***R* indices (all data)**	R_1 = 0.1055, wR_2 = 0.2190	R_1 = 0.0742, wR_2 = 0.2126

Table S1. Crystallographic data and refinement parameters for **2⊂A** and **5⊂A**.

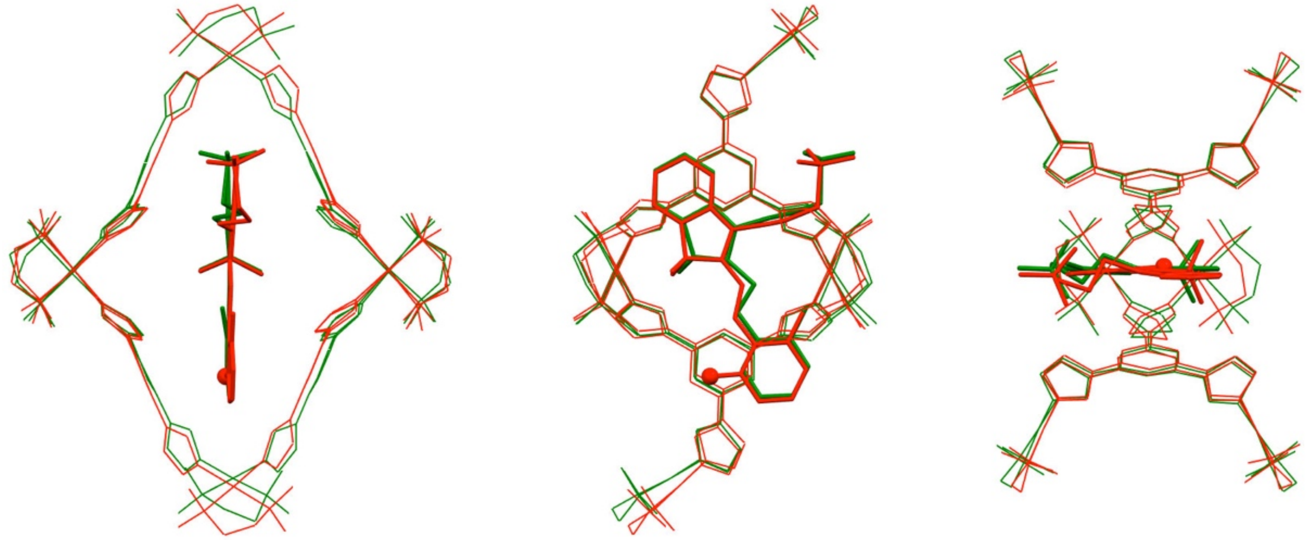

Front view Side view Top view

Figure S60. Comparison of the single-crystal X-ray structures of **2⊂A** (red) and the previously reported[2] **1⊂A** (green). Hydrogens, small ions, and water molecules were omitted for clarity. Guest **2**'s fluorine added denoted with a sphere.

8. Guest exchange saturation transfer (GEST) experiments

GEST experiments (^{19}F z-spectra): Multi-power ^{19}F-GEST experiments were performed as described before.[16] In brief, a set of presaturation pulses B_1 with a duration of 600 ms and varied powers were applied prior to the 90° radiofrequency (RF) pulse. The frequency of the presaturation pulse was swept from a $\Delta\omega$ = +13.4 ppm to a $\Delta\omega$ = –13.4 ppm offset (in 100-Hz, i.e., 0.21-ppm steps) for guests **2** and **5** and from a $\Delta\omega$ = +7.4 ppm to a $\Delta\omega$ = –7.4 ppm offset (also in 100-Hz, i.e., 0.21-ppm steps) for guest **6** (ω values relative to the resonance frequency of the encapsulated ^{19}F-guest, set to 0.0 ppm). In addition, a ^{19}F-NMR spectrum, where the RF presaturation pulse was applied at a $\Delta\omega$ of +65 ppm, was acquired as a reference spectrum (M^o). For each frequency offset, the data was acquired with 64 scans (for guests **2** and **5**) or 16 scans (for guest **6**) for sufficient signal-to-noise ratio, using a repetition time of five times of T_1. The normalized signal ($M^{\Delta\omega i}/M^o$) at each frequency offset was plotted as a function of the frequency offset of the presaturation pulse to obtain a characteristic z-spectrum for each experiment.

GEST simulations (^{19}F z-spectra): For k_{ex} estimations of each of the studied host–guest systems, the experimentally obtained z-spectra were compared to analytical solutions of the Bloch–McConnell equation (which describes the expected z-spectrum). The code for the simulations (which follows the publications by Zaiss and Bachert[17,18]) can be found at http://www.cest-sources.org.

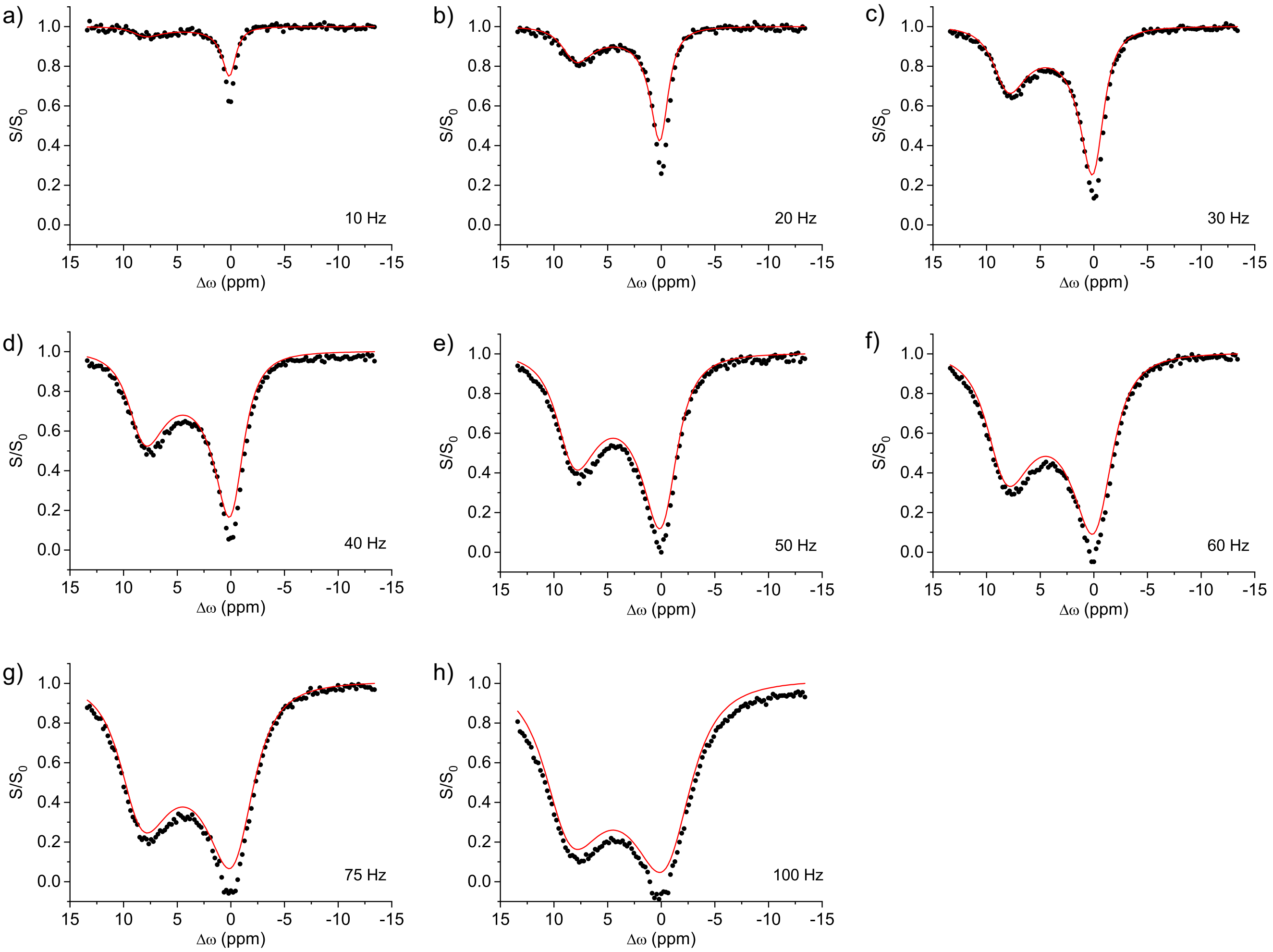

Figure S61. The z-spectra for the **2⊂A** complex in water (15 mM) recorded at different powers: 10 Hz (a), 20 Hz (b), 30 Hz (c), 40 Hz (d), 50 Hz (e), 60 Hz (f), 75 Hz (g), and 100 Hz (h). Black markers: experimental GEST data; red lines: fits from Bloch–McConnell simulations (two-pool model).

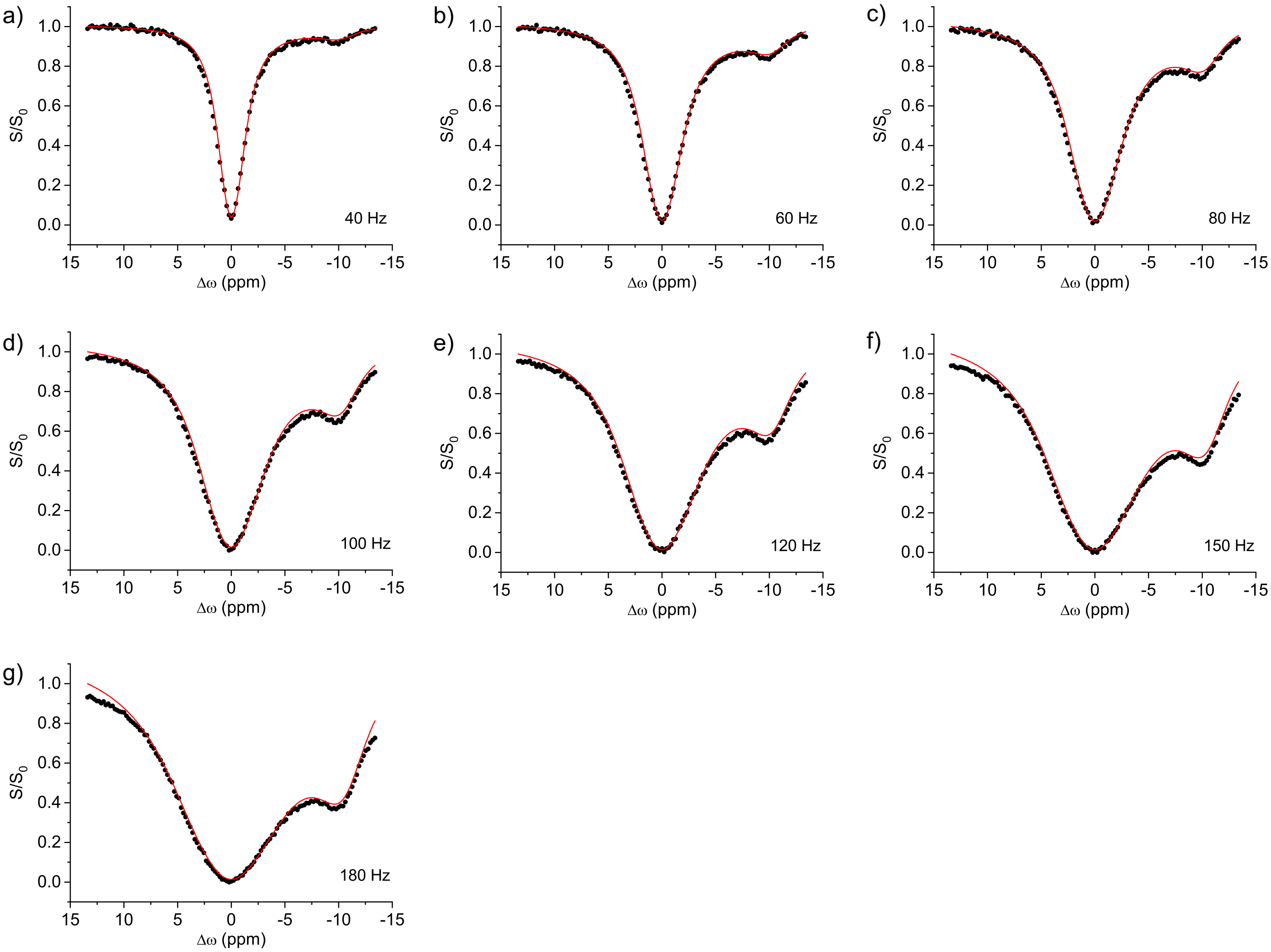

Figure S62. The z-spectra for the **5⊂A** complex in water (15 mM) recorded at different powers: 40 Hz (a), 60 Hz (b), 80 Hz (c), 100 Hz (d), 120 Hz (e), 150 Hz (f), and 180 Hz (g). Black markers: experimental GEST data; red lines: fits from Bloch–McConnell simulations (two-pool model).

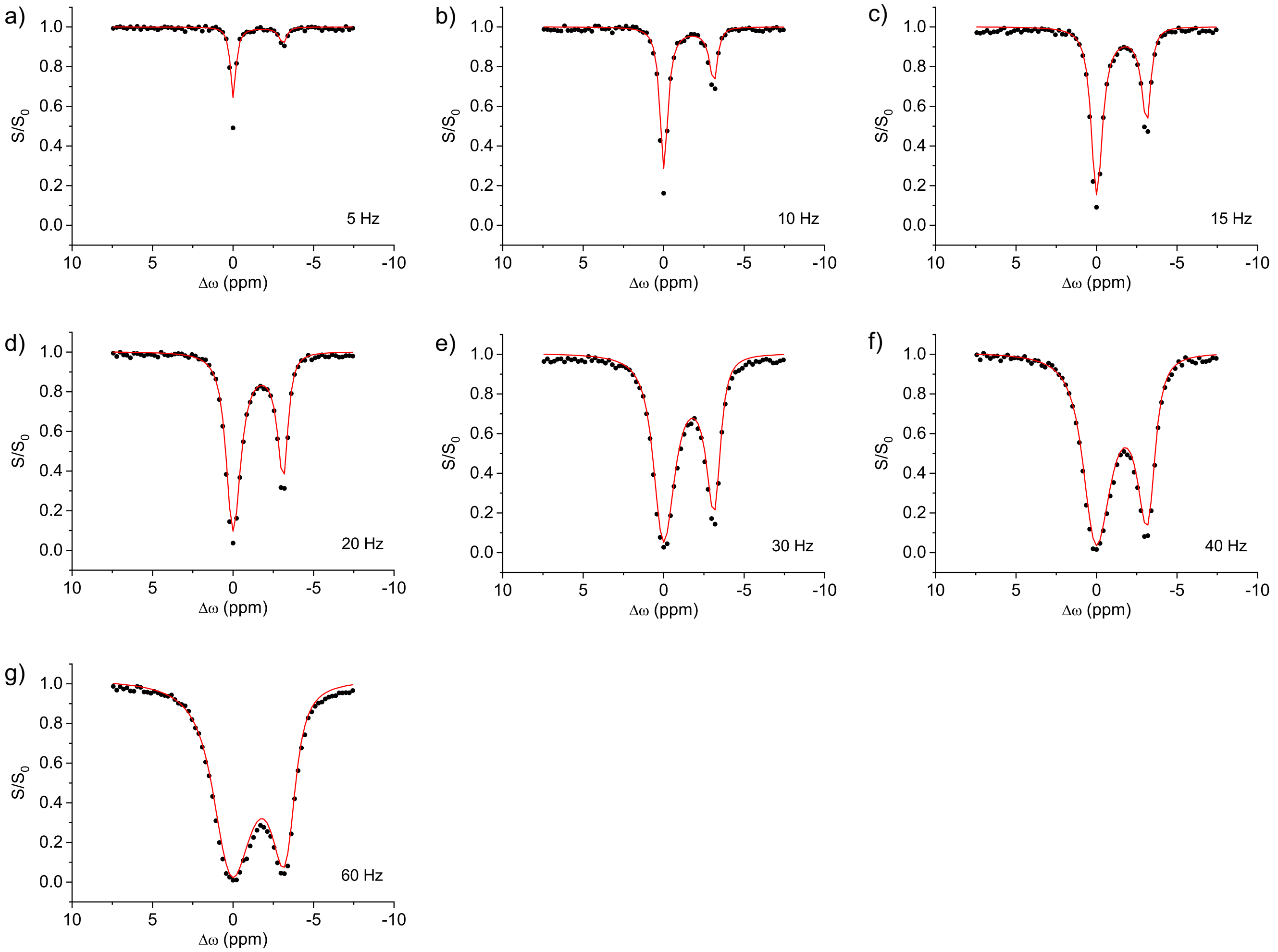

Figure S63. The z-spectra for the **6⊂A** complex in water (15 mM) recorded at different powers: 5 Hz (a), 10 Hz (b), 15 Hz (c), 20 Hz (d), 30 Hz (e), 40 Hz (f), and 60 Hz (g). Black markers: experimental GEST data; red lines: fits from Bloch–McConnell simulations (two-pool model).

9. Photoresponsive properties of free and encapsulated spiropyrans

9.1. Photoswitching of free spiropyrans

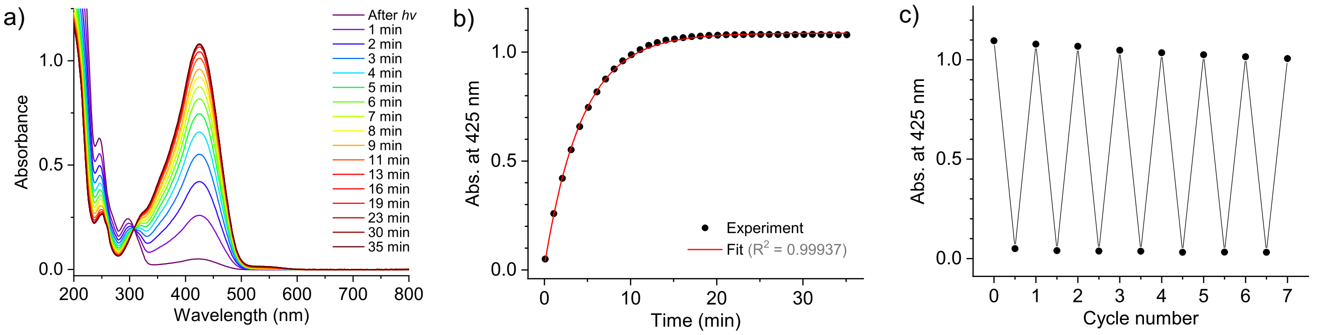

Figure S64. (a) A series of UV/vis spectra of **1**$_{MCH}$ (50 μM in water with 2% *v*/*v* methanol) immediately after irradiation with 420 nm light (MCH → SP + H$^+$) and after different periods in the dark. (b) Spontaneous recovery of MCH in the dark (MCH → SP + H$^+$) by monitoring the solution's absorbance at 425 nm (the wavelength of maximum absorption of **1**$_{MCH}$ in water); fitting to a first-order rate equation gives a rate constant of $k = 4.46$ min^{-1}. (c) Seven cycles of reversible photoisomerization (30 s of irradiation per cycle) followed by UV/vis spectroscopy.

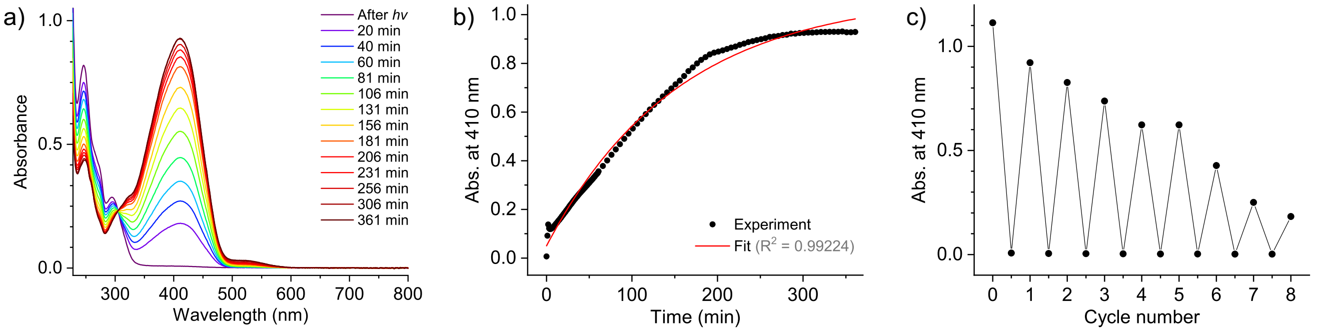

Figure S65. (a) A series of UV/vis spectra of **2**$_{MCH}$ (50 μM in water with 2% *v*/*v* methanol) immediately after irradiation with 420 nm light and after different periods in the dark. (b) Spontaneous recovery of MCH in the dark by monitoring the solution's absorbance at 410 nm (the wavelength of maximum absorption of **2**$_{MCH}$ in water); fitting to a first-order rate equation gives a rate constant of k = 0.0065 min^{-1}. (c) Eight cycles of reversible photoisomerization (30 s of irradiation per cycle) followed by UV/vis spectroscopy.

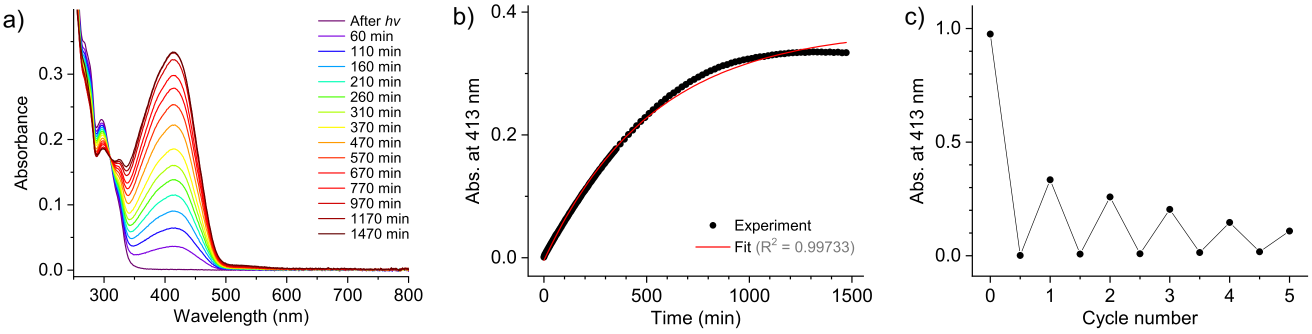

Figure S66. (a) A series of UV/vis spectra of $\mathbf{3}_{\mathrm{MCH}}$ (50 μM in water with 2% *v*/*v* methanol) immediately after irradiation with 420 nm light and after different periods in the dark. (b) Spontaneous recovery of MCH in the dark by monitoring the solution's absorbance at 413 nm (the wavelength of maximum absorption of $\mathbf{3}_{\mathrm{MCH}}$ in water); fitting to a first-order rate equation gives a rate constant of k = 0.0019 min^{-1}. (c) Five cycles of reversible photoisomerization (30 s of irradiation per cycle) followed by UV/vis spectroscopy.

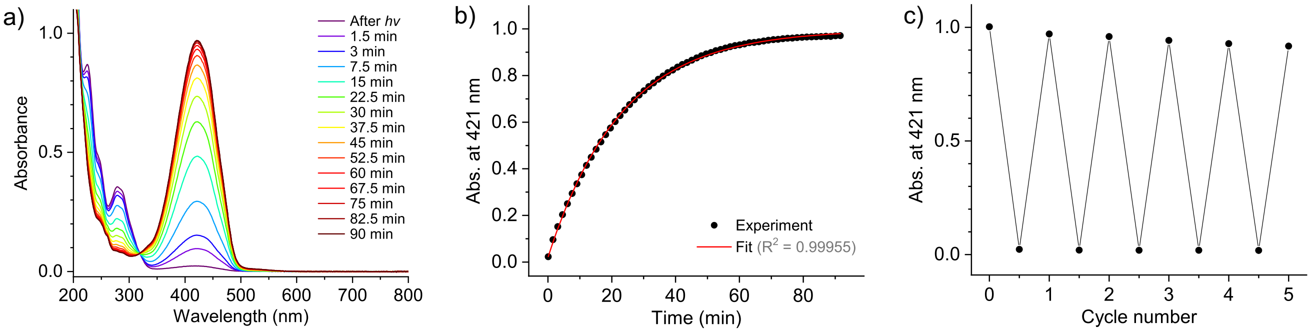

Figure S67. (a) A series of UV/vis spectra of $\mathbf{4}_{\mathrm{MCH}}$ (50 μM in water with 2% *v*/*v* methanol) immediately after irradiation with 420 nm light and after different periods in the dark. (b) Spontaneous recovery of MCH in the dark by monitoring the solution's absorbance at 421 nm (the wavelength of maximum absorption of $\mathbf{4}_{\mathrm{MCH}}$ in water); fitting to a first-order rate equation gives a rate constant of k = 0.044 min^{-1}. (c) Five cycles of reversible photoisomerization (30 s of irradiation per cycle) followed by UV/vis spectroscopy.

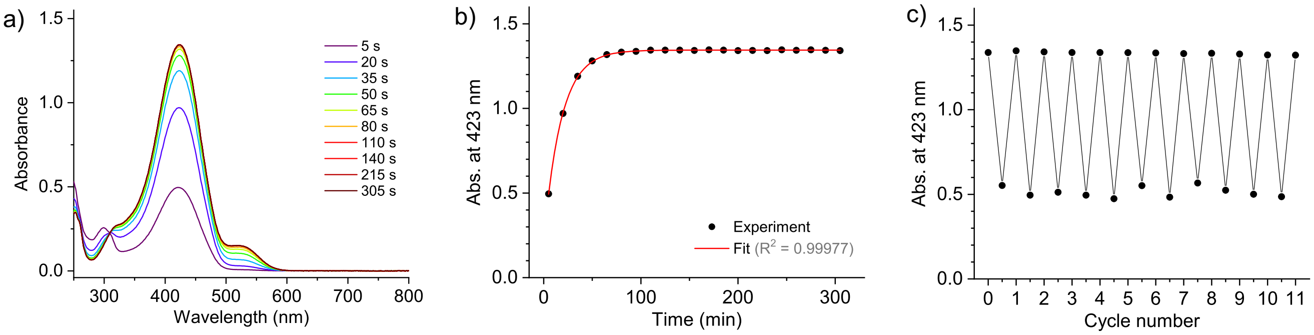

Figure S68. (a) A series of UV/vis spectra of $\mathbf{5}_{\mathrm{MCH}}$ (50 μM in water with 2% *v*/*v* methanol) immediately after irradiation with 420 nm light and after different periods in the dark. (b) Spontaneous recovery of MCH in the dark by monitoring the solution's absorbance at 423 nm (the wavelength of maximum absorption of $\mathbf{4}_{\mathrm{MCH}}$ in water); fitting to a first-order rate equation gives a rate constant of k = 0.056 min^{-1}. (c) Eleven cycles of reversible photoisomerization (30 s of irradiation per cycle) followed by UV/vis spectroscopy.

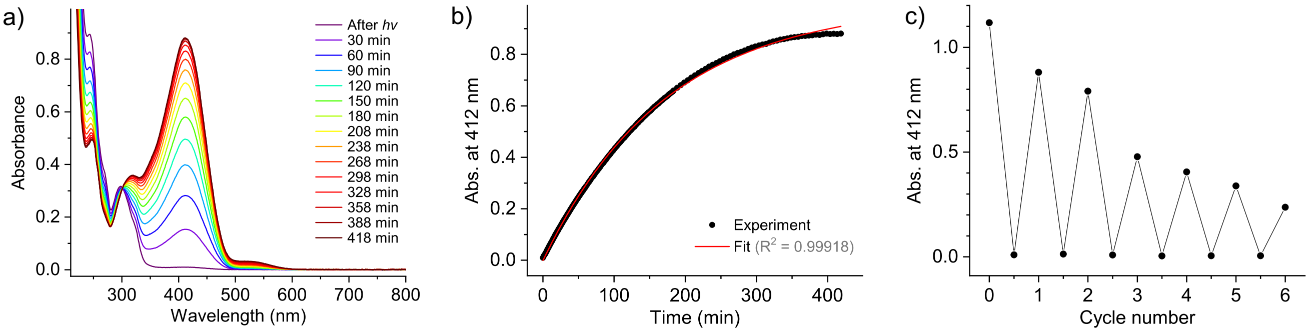

Figure S69. (a) A series of UV/vis spectra of $\mathbf{6}_{\mathrm{MCH}}$ (50 µM in water with 2% *v*/*v* methanol) immediately after irradiation with 420 nm light and after different periods in the dark. (b) Spontaneous recovery of MCH in the dark by monitoring the solution's absorbance at 412 nm (the wavelength of maximum absorption of $\mathbf{6}_{\mathrm{MCH}}$ in water); fitting to a first-order rate equation gives a rate constant of k = 0.0058 min^{-1}. (c) Six cycles of reversible photoisomerization (30 s of irradiation per cycle) followed by UV/vis spectroscopy.

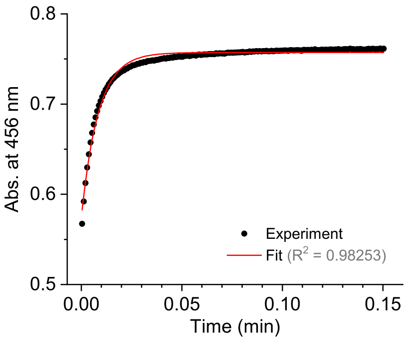

Figure S70. Spontaneous recovery of $\mathbf{7}_{\mathrm{MCH}}$ in the dark by monitoring the solution's absorbance at 456 nm (the wavelength of maximum absorption of $\mathbf{7}_{\mathrm{MCH}}$ in water) (50 µM in water with 2% *v*/*v* methanol). Fitting to a first-order rate equation gives a rate constant of k = 119 min^{-1}. Because of the very fast back-switching, it was not possible to record full-range UV/vis spectra.

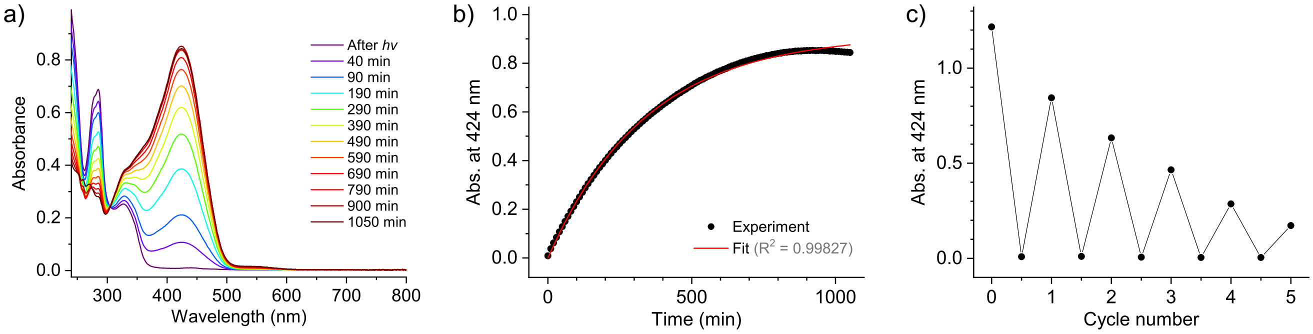

Figure S71. (a) A series of UV/vis spectra of $\mathbf{8}_{\mathrm{MCH}}$ (50 µM in water with 2% *v*/*v* methanol) immediately after irradiation with 420 nm light and after different periods in the dark. (b) Spontaneous recovery of MCH in the dark by monitoring the solution's absorbance at 425 nm (the wavelength of maximum absorption of $\mathbf{8}_{\mathrm{MCH}}$ in water); fitting to a first-order rate equation gives a rate constant of k = 0.0029 min^{-1}. (c) Five cycles of reversible photoisomerization (30 s of irradiation per cycle) followed by UV/vis spectroscopy.

9.2. Photoswitching of spiropyrans within cage **A**

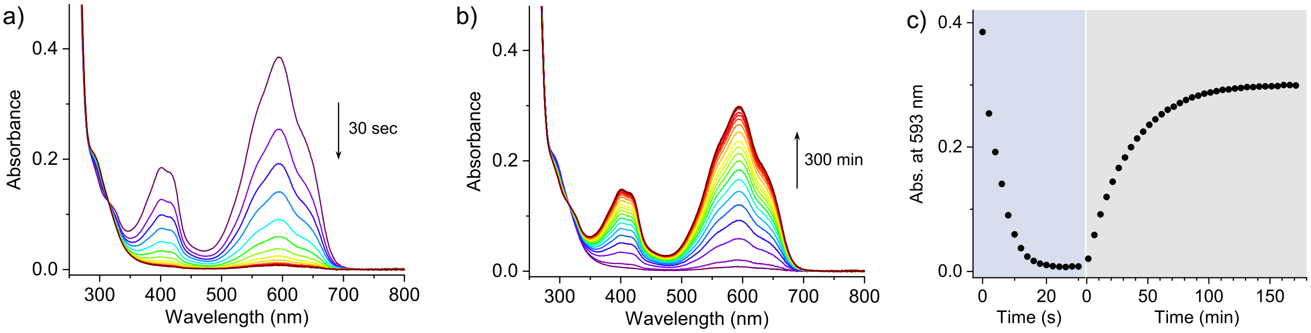

Figure S72. (a) Evolution of the UV/vis spectra of **1**⊂**A** (20 μM in water) during irradiation with 420 nm light. (b) Thermal recovery of **1**$_{MC}$⊂**A** in the dark followed by UV/vis spectroscopy. (c) Following the reversible isomerization of **1**⊂**A** by monitoring the absorbance at 593 nm; blue shading: 420 nm light irradiation; gray shading: incubation in the dark.

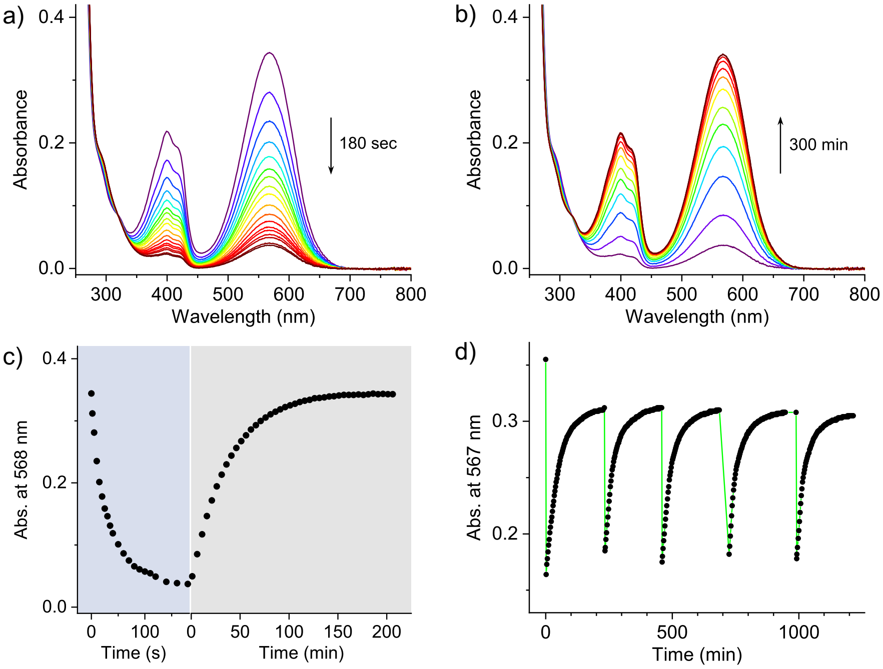

Figure S73. (a) Evolution of the UV/vis spectra of **2**⊂**A** (20 μM in water) during irradiation with 460 nm light. (b) Thermal recovery of **2**$_{MC}$⊂**A** in the dark followed by UV/vis spectroscopy. (c) Following the reversible isomerization of **2**⊂**A** by monitoring the absorbance at 568 nm; blue shading: 460 nm light irradiation; gray shading: incubation in the dark. (d) Five cycles of reversible photoisomerization of **2** within **A** followed by UV/vis spectroscopy (30 s of 460 nm light irradiation per cycle).

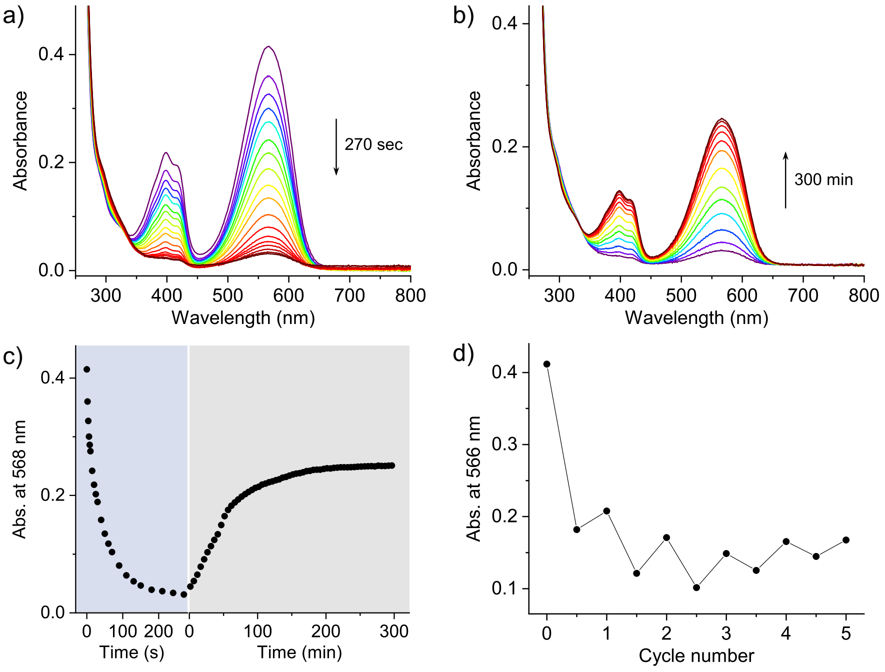

Figure S74. (a) Evolution of the UV/vis spectra of **3**⊂**A** (20 μM in water) during irradiation with 460 nm light. (b) Thermal recovery of $\mathbf{3}_{\mathrm{MC}}$⊂**A** in the dark followed by UV/vis spectroscopy. (c) Following the reversible isomerization of **3**⊂**A** by monitoring the absorbance at 568 nm; blue shading: 460 nm light irradiation; gray shading: incubation in the dark. (d) Five cycles of photoisomerization followed by UV/vis spectroscopy (30 s of 460 nm light irradiation per cycle).

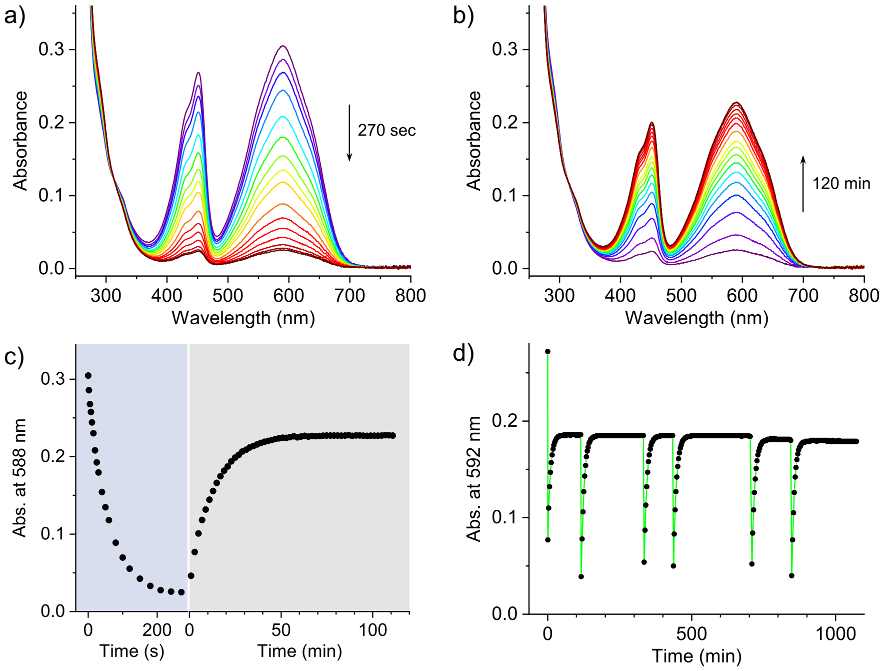

Figure S75. (a) Evolution of the UV/vis spectra of **4**⊂**A** (20 μM in water) during irradiation with 420 nm light. (b) Thermal recovery of $\mathbf{4}_{\mathrm{MC}}$⊂**A** in the dark followed by UV/vis spectroscopy. (c) Following the reversible isomerization of **4**⊂**A** by monitoring the absorbance at 588 nm; blue shading: 420 nm light irradiation; gray shading: incubation in the dark. (d) Six cycles of reversible photoisomerization followed by UV/vis spectroscopy (60 s of 460 nm light irradiation per cycle).

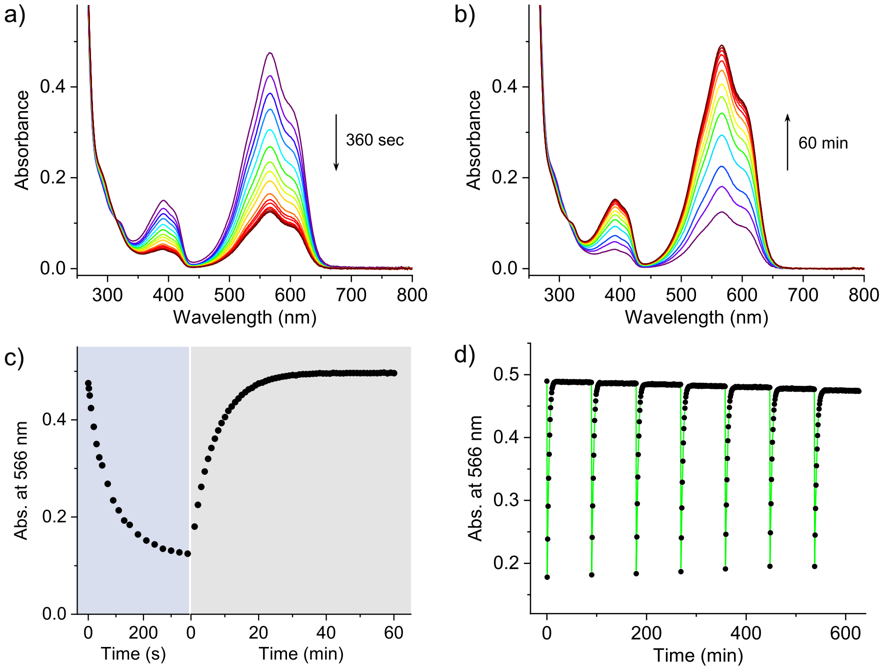

Figure S76. (a) Evolution of the UV/vis spectra of $\mathbf{5}\subset\mathbf{A}$ (20 μM in water) during irradiation with 460 nm light. (b) Thermal recovery of $\mathbf{5}_{MC}\subset\mathbf{A}$ in the dark followed by UV/vis spectroscopy. (c) Following the reversible isomerization of $\mathbf{5}\subset\mathbf{A}$ by monitoring the absorbance at 566 nm; blue shading: 420 nm light irradiation; gray shading: incubation in the dark. (d) Seven cycles of reversible photoisomerization followed by UV/vis spectroscopy (30 s of 460 nm light irradiation per cycle).

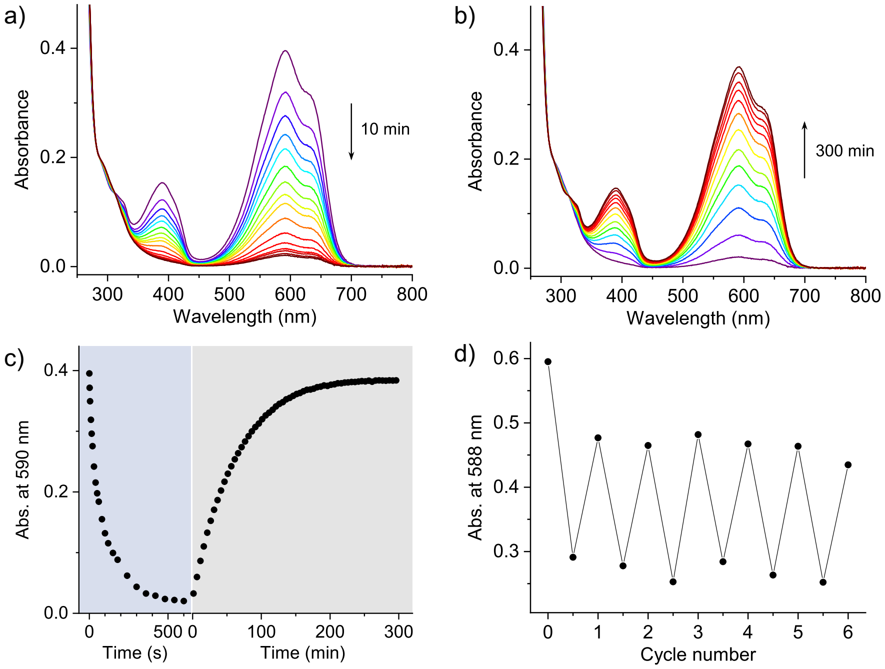

Figure S77. (a) Evolution of the UV/vis spectra of $\mathbf{6}\subset\mathbf{A}$ (20 μM in water) during irradiation with 460 nm light. (b) Thermal recovery of $\mathbf{6}_{MC}\subset\mathbf{A}$ in the dark followed by UV/vis spectroscopy. (c) Following the reversible isomerization of $\mathbf{6}\subset\mathbf{A}$ by monitoring the absorbance at 590 nm; blue shading: 420 nm light irradiation; gray shading: incubation in the dark. (d) Six cycles of reversible photoisomerization followed by UV/vis spectroscopy (30 s of 460 nm light irradiation per cycle).

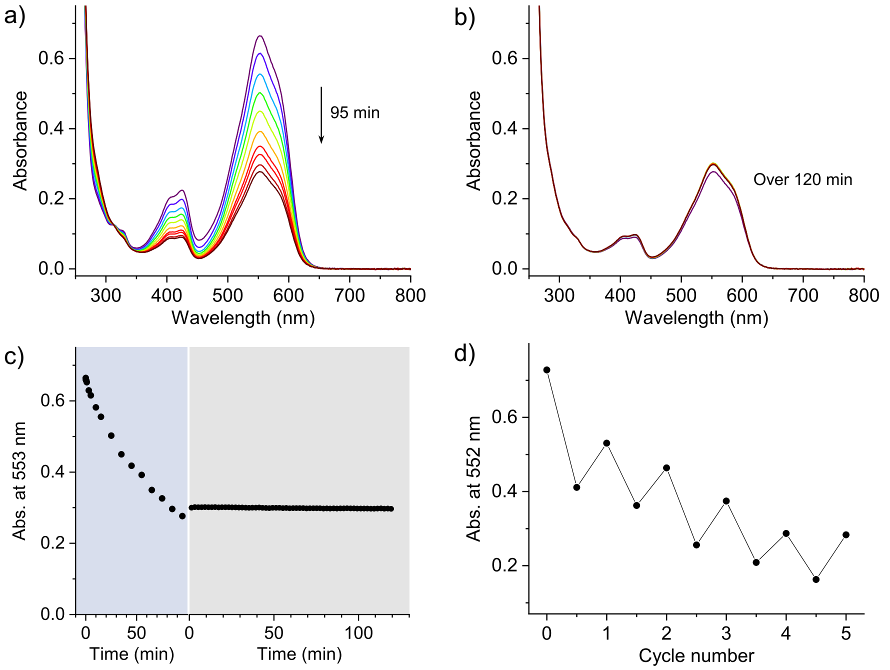

Figure S78. (a) Evolution of the UV/vis spectra of **7**⊂**A** (20 μM in water) during irradiation with 420 nm light. (b) Thermal recovery of $\mathbf{7}_{\mathrm{MC}}$⊂**A** in the dark followed by UV/vis spectroscopy. (c) Following the reversible isomerization of **7**⊂**A** by monitoring the absorbance at 552 nm. (d) Five cycles of photoisomerization followed by UV/vis spectroscopy (60 s of 460 nm light irradiation per cycle).

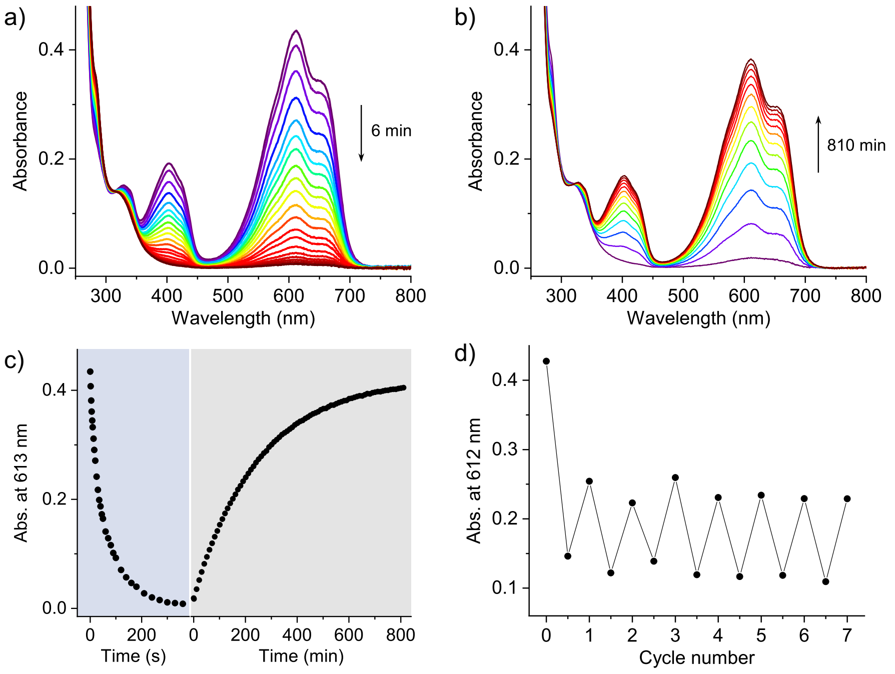

Figure S79. (a) Evolution of the UV/vis spectra of **8**⊂**A** (20 μM in water) during irradiation with 420 nm light. (b) Thermal recovery of $\mathbf{8}_{\mathrm{MC}}$⊂**A** in the dark followed by UV/vis spectroscopy. (c) Following the reversible isomerization of **8**⊂**A** by monitoring the absorbance at 613 nm. (d) Seven cycles of reversible photoisomerization followed by UV/vis spectroscopy (30 s of 460 nm light irradiation per cycle).

Photoisomerization of spiropyrans encapsulated within cage **A** can also be followed by NMR, as illustrated below for **4⊂A**.

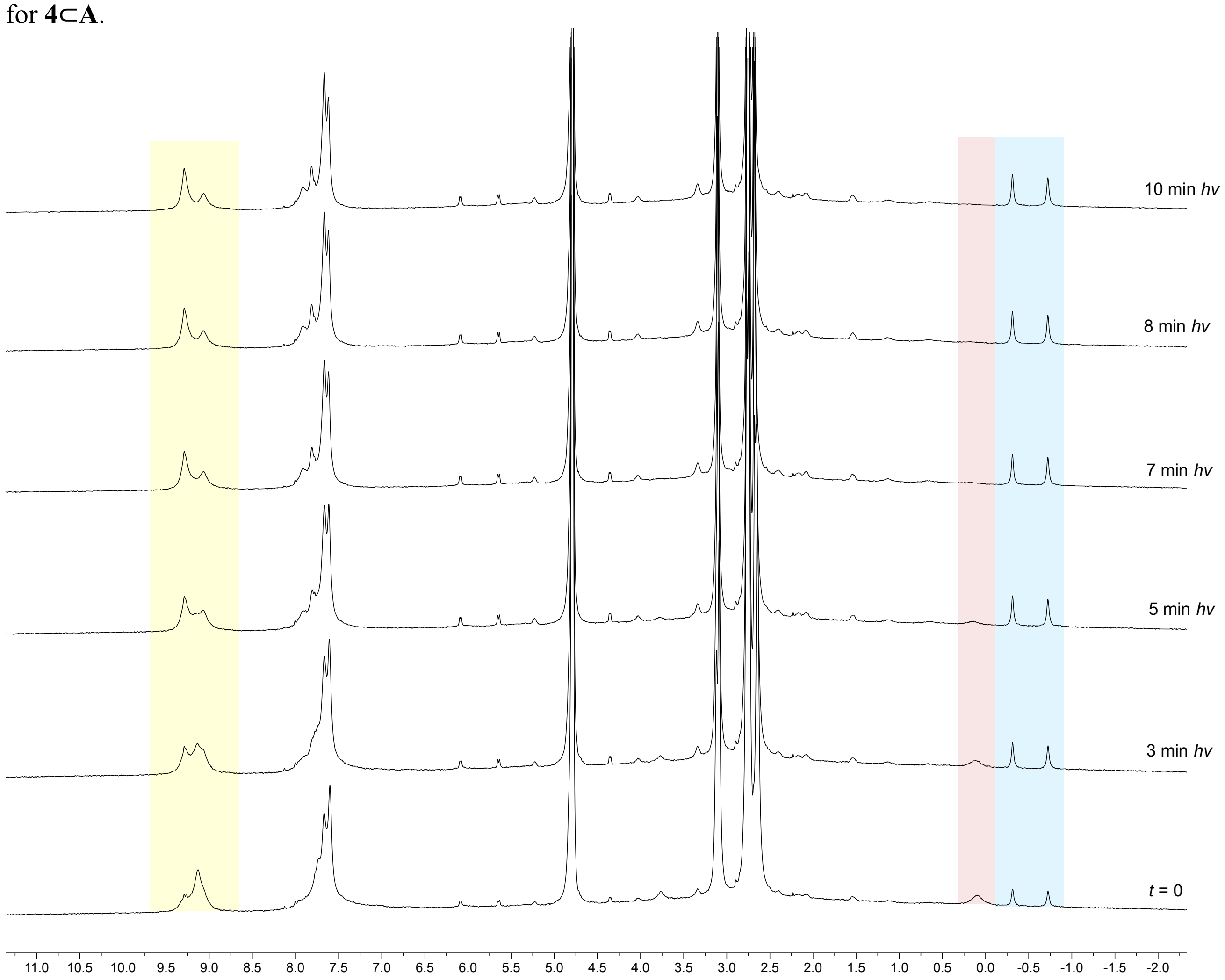

Figure S80. Evolution of the ^{1}H NMR spectra of **4⊂A** (500 MHz, D_2O, 298 K) during in-situ irradiation with 460 nm LED light for up to 10 min. The signal originating from $\mathbf{4}_{MC}$'s C**H**$_3$ protons are highlighted in red; those originating from $\mathbf{4}_{SP}$'s C**H**$_3$ protons are highlighted in blue. The **A**'s acidic imidazole protons are highlighted in yellow. Note that this particular spiropyran (**4**) within **A** contains sizeable fraction of the SP form even prior to light irradiation (see Figure S44 above and Figure 2e in the main text).

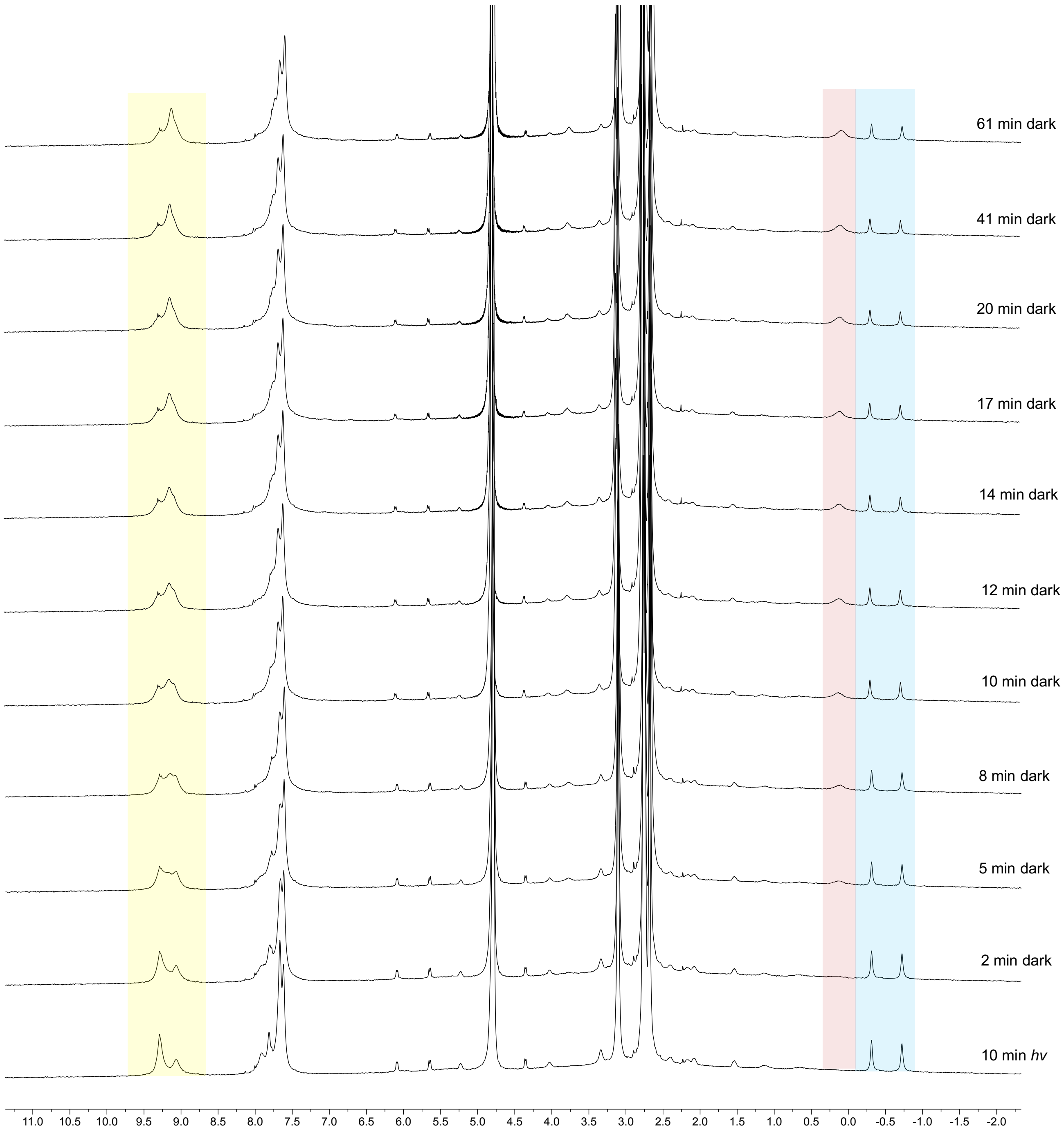

Figure S81. Following the thermal back-isomerization of $\mathbf{4}_{SP}\subset\mathbf{A}$ into $\mathbf{4}_{MC}\subset\mathbf{A}$ by ^{1}H NMR (500 MHz, D_2O, 298 K). The sample had been pre-irradiated with 460 nm light for 10 min (see Figure S80). The signal originating from $\mathbf{4}_{MC}$'s C$\mathbf{H}_3$ protons are highlighted in red and those originating from $\mathbf{4}_{SP}$'s C$\mathbf{H}_3$ protons are highlighted in blue. Also note the changes in the **A**'s acidic imidazole protons, highlighted in yellow.

Finally, we studied the effect of UV light on the equilibrium between encapsulated $\mathbf{1}_{MC}$ and $\mathbf{1}_{SP}$. To this end, we prepared a solution of $\mathbf{1}_{MC}$⊂**A** (Figure 82a, red) and exposed it to blue light (460 nm) for 30 sec, inducing photoisomerization to $\mathbf{1}_{SP}$⊂**A**, with negligible absorption in the visible region. Having determined that $\mathbf{1}_{SP}$⊂**A** recovers to $\mathbf{1}_{MC}$⊂**A** within several minutes (Figure S72b, c), we hypothesized that back-isomerization would proceed faster under UV light – the stimulus typically used to induce ring-opening of SP into MC. Surprisingly, however, the opposite was observed: the system was maintained in the SP state for as long as UV light was on (in the dark, back-isomerization proceeded as in Figure S72b, c). Analogous results were seen found for **4⊂A**, whose unique feature is that it contains a high fraction of the SP form at equilibrium (Figure 2e): upon exposure to UV, absorbance in the visible region decreased instead of increasing (Figure S82b). Once the light was turned off, the original spectrum was regenerated.

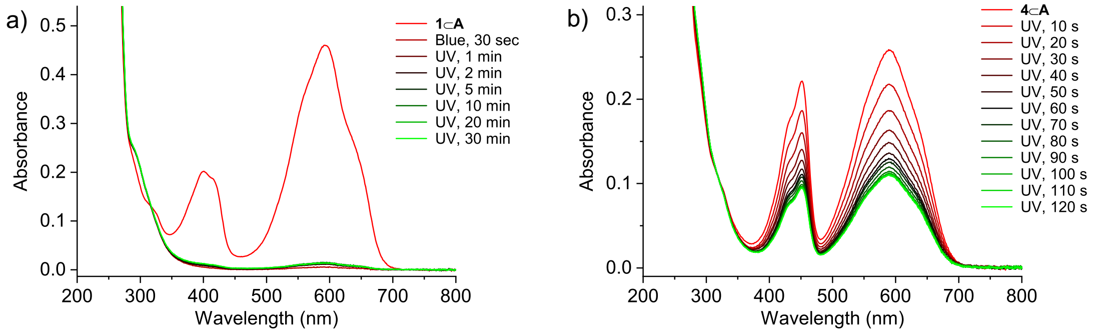

Figure S82. Evolution of the UV/vis spectra of **1⊂A** (a) and **4⊂A** (b) (both in water) during irradiation with a UV LED.

These results can be explained by the combination of the cage's high UV absorption coefficient, its optical transparency in the visible region, and the polychromatic nature of our UV LED. Although the emission peak of the LED is centered at 365 nm, its emission extends into the blue region. Blue light is very effective in inducing the isomerization of MC into SP; on the other hand, a substantial amount of UV light emitted by the LED can be absorbed by the cage. Although it is difficult to assess to what extent the SP→MC reaction occurs, the reverse reaction is obviously more efficient, shifting the equilibrium towards the SP form.

9.3. Attempts to photoswitch spiropyrans within cage **B**

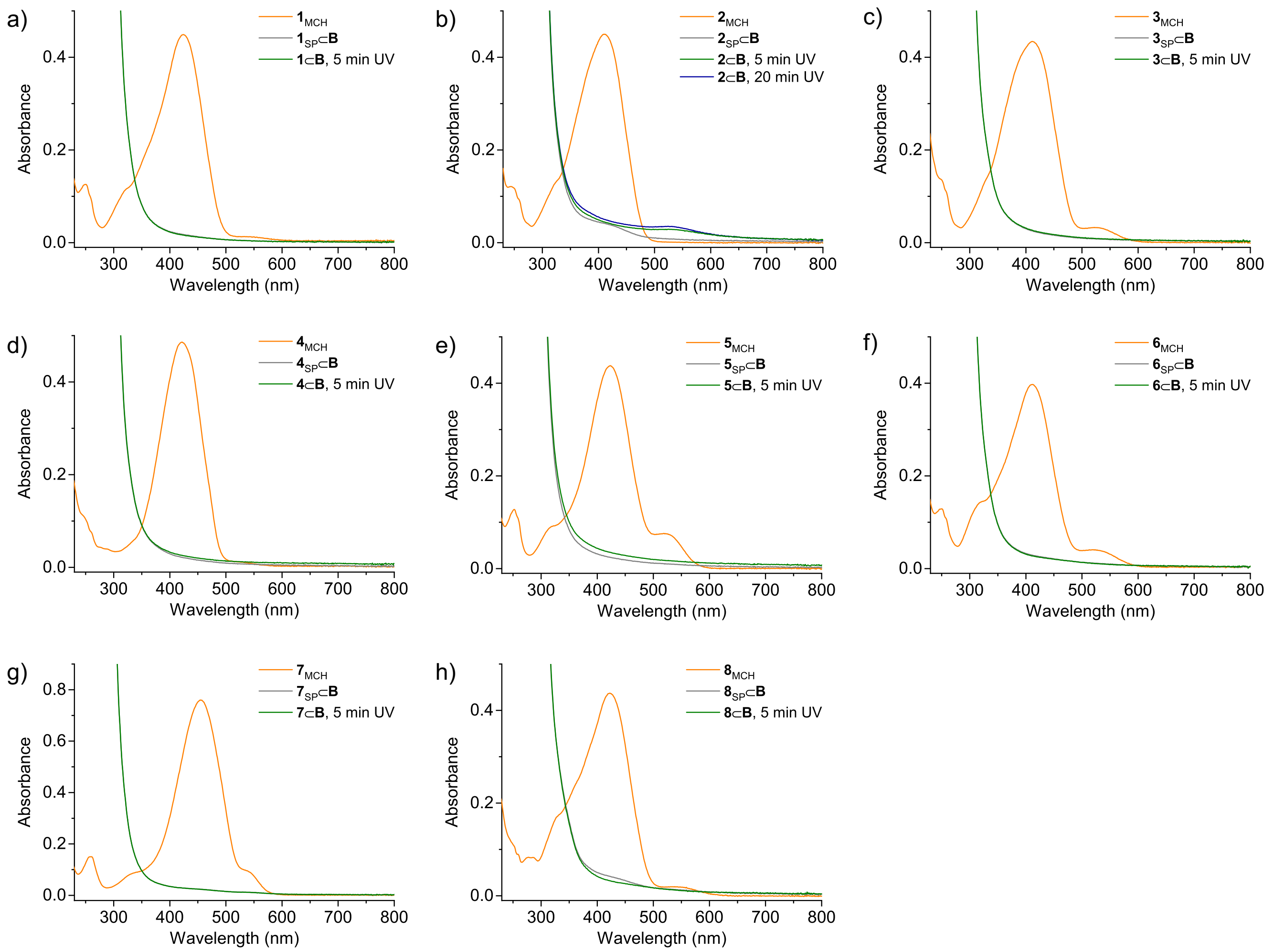

Figure S83. UV/vis absorption spectra of spiropyrans **1**–**8** (orange) and their inclusion complexes with cage **B** before (gray) and after (green) exposure to UV light (365 nm) for 4 min. No noticeable changes in the spectra can be observed. Note that in b), we intentionally kept a small fraction of spiropyran uncomplexed (by adding a smaller amount of the cage) to demonstrate that the free spiropyran remains photoisomerizable. This small amount of uncomplexed spiropyran (in the MCH form) is seen as a low-intensity band at ~420 nm in the gray spectrum in b). UV irradiation results in the appearance of another low-intensity band at ~540 nm, characteristic of the MC form in a highly polar environment (here, water).

10. DFT calculations

DFT calculations were performed at the B3LYP-D3/6-31G(d,p)/LANL2DZ(Pd) level of theory[19,20] using the Gaussian 16 software.[21] The structure was optimized as a dication with the symmetric distribution of nitrate anions around the cage (see file "1SPcA.pdb" attached to Supporting Information online). The optimization was performed in vacuo. The starting geometry was taken from the crystal structure of an open form of the guest ($\mathbf{1}_{MC}\subset\mathbf{A}$) and $\mathbf{1}_{MC}$ was transformed into $\mathbf{1}_{SP}$. No imaginary frequencies were found, thus confirming that the energetic minimum of the optimized model was obtained. Geometry preoptimization in implicit water led to a very similar result.

11. Extraction of guests from cage **B** to cage **A**

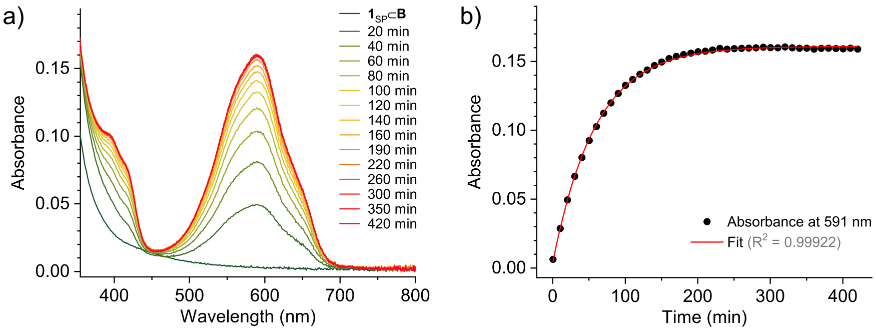

Figure S84. (a) Evolution of UV/vis spectra of **1**$_{SP}$⊂**B** (in the presence of 4 extra equivalents of **B** to ensure complete encapsulation of **1**) upon injecting 5 equiv of cage **A**. (b) Following the reaction by monitoring the absorbance at 591 nm (originating from **1**$_{MC}$ within **A**); fitting to a first-order rate equation gives a rate constant of $k = 0.017$ min^{-1}.

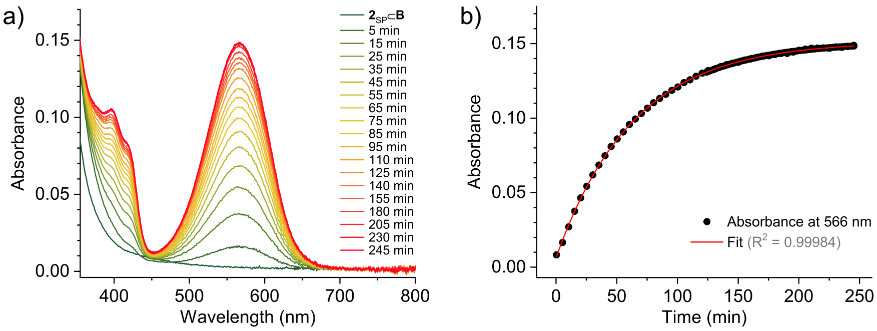

Figure S85. (a) Evolution of UV/vis spectra of **2**$_{SP}$⊂**B** (in the presence of 4 extra equivalents of **B** to ensure complete encapsulation of **2**) upon injecting 5 equiv of cage **A**. (b) Following the reaction by monitoring the absorbance at 566 nm (originating from **2**$_{MC}$ within **A**); fitting to a first-order rate equation gives a rate constant of $k = 0.016$ min^{-1}. We note that ~6% of **2** within **A** exists in the colorless SP form; therefore, the actual rate constant = k / 0.94 = 0.017 min^{-1}.

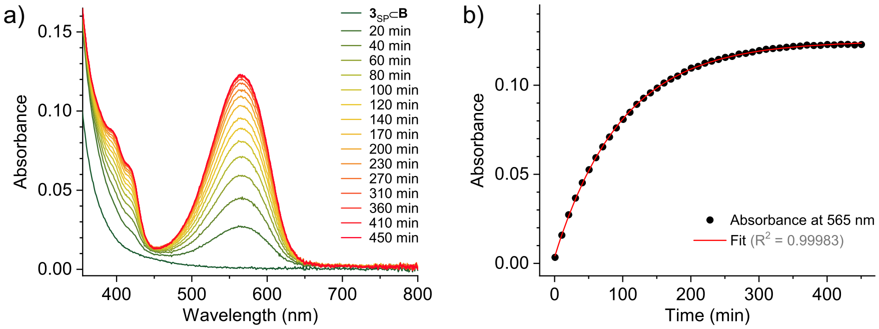

Figure S86. (a) Evolution of UV/vis spectra of **3**$_{SP}$⊂**B** (in the presence of 4 extra equivalents of **B** to ensure complete encapsulation of **3**) upon injecting 5 equiv of cage **A**. (b) Following the reaction by monitoring the absorbance at 565 nm (originating from **3**$_{MC}$ within **A**); fitting to a first-order rate equation gives a rate constant of $k = 0.010$ min^{-1}. We note that ~24% of **3** within **A** exists in the colorless SP form; therefore, the actual rate constant = k / 0.76 = 0.013 min^{-1}.

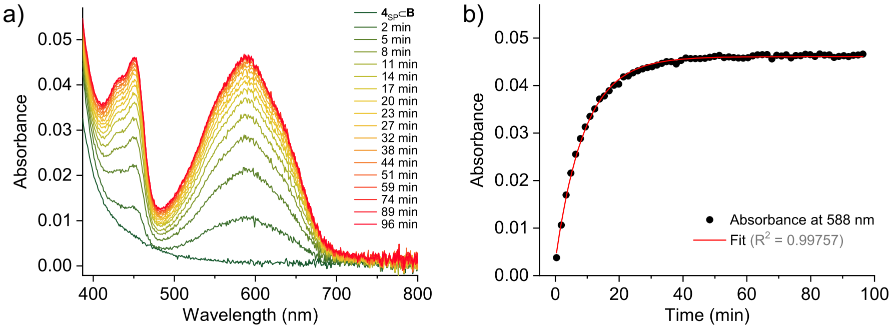

Figure S87. (a) Evolution of UV/vis spectra of $\mathbf{4}_{SP}\subset\mathbf{B}$ (in the presence of 4 extra equivalents of **B** to ensure complete encapsulation of **4**) upon injecting 5 equiv of cage **A**. (b) Following the reaction by monitoring the absorbance at 588 nm (originating from $\mathbf{4}_{MC}$ within **A**); fitting to a first-order rate equation gives a rate constant of k = 0.11 min^{-1}. We note that ~40% of **3** within **A** exists in the colorless SP form; therefore, the actual rate constant = k / 0.60 = 0.18 min^{-1}.

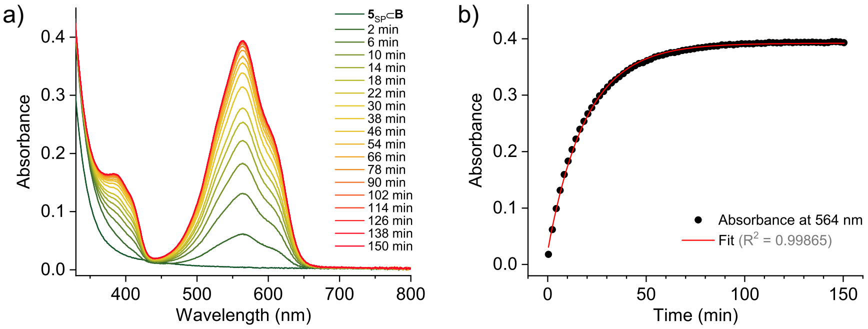

Figure S88. (a) Evolution of UV/vis spectra of $\mathbf{5}_{SP}\subset\mathbf{B}$ (in the presence of 4 extra equivalents of **B** to ensure complete encapsulation of **5**) upon injecting 5 equiv of cage **A**. (b) Following the reaction by monitoring the absorbance at 564 nm (originating from $\mathbf{5}_{MC}$ within **A**); fitting to a first-order rate equation gives a rate constant of k = 0.052 min^{-1}.

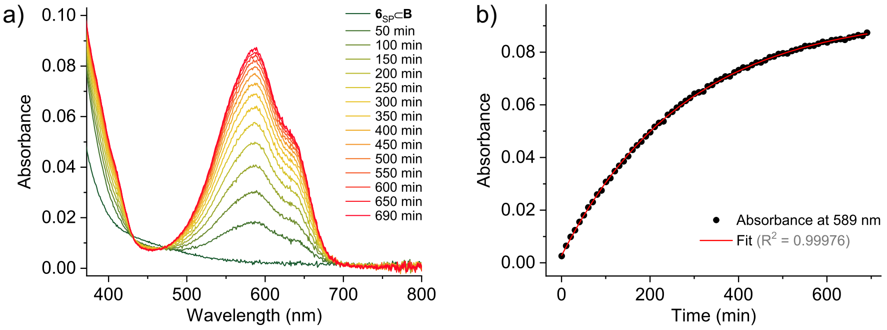

Figure S89. (a) Evolution of UV/vis spectra of $\mathbf{6}_{SP}\subset\mathbf{B}$ (in the presence of 4 extra equivalents of **B** to ensure complete encapsulation of **6**) upon injecting 5 equiv of cage **A**. (b) Following the reaction by monitoring the absorbance at 589 nm (originating from $\mathbf{6}_{MC}$ within **A**); fitting to a first-order rate equation gives a rate constant of k = 0.036 min^{-1}.

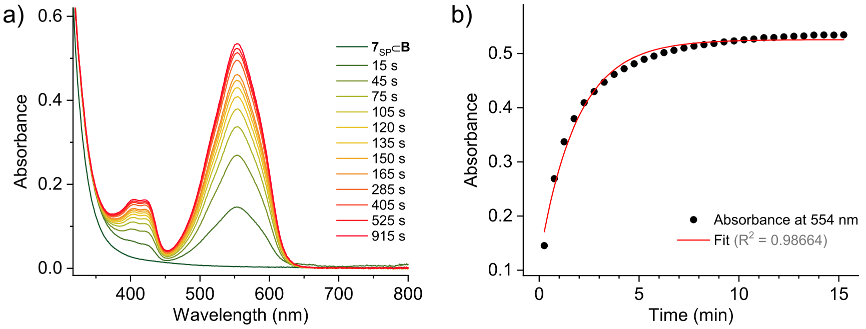

Figure S90. (a) Evolution of UV/vis spectra of $\mathbf{7}_{\mathrm{SP}}\subset\mathbf{B}$ (in the presence of 4 extra equivalents of **B** to ensure complete encapsulation of **7**) upon injecting 5 equiv of cage **A**. (b) Following the reaction by monitoring the absorbance at 554 nm (originating from $\mathbf{7}_{\mathrm{MC}}$ within **A**); fitting to a first-order rate equation gives a rate constant of $k = 0.529\ \mathrm{min}^{-1}$.

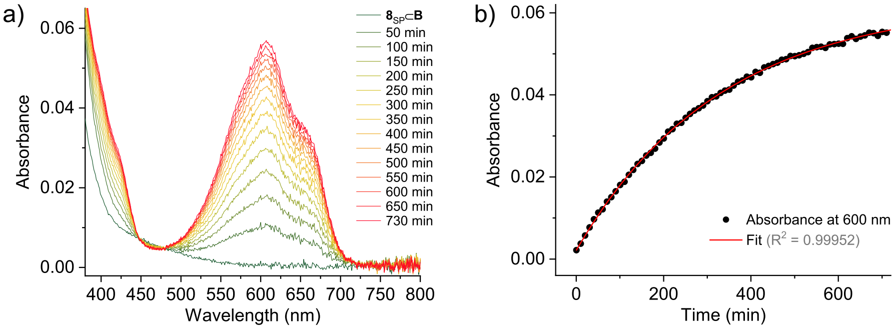

Figure S91. (a) Evolution of UV/vis spectra of $\mathbf{8}_{\mathrm{SP}}\subset\mathbf{B}$ (in the presence of 4 extra equivalents of **B** to ensure complete encapsulation of **8**) upon injecting 5 equiv of cage **A**. (b) Following the reaction by monitoring the absorbance at 600 nm (originating from $\mathbf{8}_{\mathrm{MC}}$ within **A**); fitting to a first-order rate equation gives a rate constant of $k = 0.003\ \mathrm{min}^{-1}$.

12. Preparation of photoresponsive agarose gels

One gram of agarose (CAS # 9012-36-6, biotechnology grade, Amresco product # 0710) was added to an Erlenmeyer flask containing 50 mL of distilled water. The mixture was heated in a microwave oven until water started to boil. After an additional 3 min, heating was discontinued and the flask was removed from the oven. The resulting colorless, homogeneous solution was poured while hot between two glass slides separated by 1 mm spacers; any air bubbles were removed by gentle tapping on the glass slides while hot. After having been cooled to room temperature, a 4×3.5×0.1 cm piece of solidified agarose gel was cut using a cutter knife and placed in a Petri dish containing a 2 mM aqueous solution of **5⊂A** or **6⊂A**. After 30 min of gentle shaking, the gel was briefly rinsed with water.

13. Supporting references

1. Samanta, D.; Mukherjee, S.; Patil, Y. P.; Mukherjee, P. S. Self-assembled Pd_6 open cage with triimidazole walls and the use of its confined nanospace for catalytic Knoevenagel- and Diels–Alder reactions in aqueous medium. *Chem. Eur. J.* **2012**, *18*, 12322–12329.
2. Samanta, D.; Galaktionova, D.; Gemen, J.; Shimon, L. J. W.; Diskin-Posner, Y.; Avram, L.; Král, P.; Klajn, R. Reversible chromism of spiropyran in the cavity of a flexible coordination cage. *Nat. Commun.* **2018**, *9*, 641.
3. Yoshizawa, M.; Miyagi, S.; Kawano, M.; Ishiguro, K.; Fujita, M. Alkane oxidation via photochemical excitation of a self-assembled molecular cage. *J. Am. Chem. Soc.* **2004**, *126*, 9172–9173.
4. Shi, Z.; Peng, P.; Strohecker, D.; Liao, Y. Long-lived photoacid based upon a photochromic reaction. *J. Am. Chem. Soc.* **2011**, *133*, 14699–14703.
5. Akselsen, Ø. W.; Skattebøl, L.; Hansen, T. V. *ortho*-Formylation of oxygenated phenols. *Tetrahedron Lett.* **2009**, *50*, 6339–6341.
6. Aldred, R.; Johnston, R.; Levin, D.; Neilan, J. Magnesium-mediated *ortho*-specific formylation and formaldoximation of phenols. *J. Chem. Soc. Perkin Trans. 1* **1994**, 1823–1831.
7. Zacharie, B.; Attardo, G.; Barriault, N.; Penney, C. Regioselective synthesis of 6-substituted 2-hydroxybenzaldehyde: efficient synthesis of the immunomodulator tucaresol and related analogues. *J. Chem. Soc. Perkin Trans. 1* **1997**, 2925–2929.
8. Haight, A. R.; Bailey, A. E.; Baker, W. S.; Cain, M. H.; Copp, R. R.; DeMattei, J. A.; Ford, K. L.; Henry, R. F.; Hsu, M. C.; Keyes, R. F.; King, S. A.; McLaughlin, M. A.; Melcher, L. M.; Nadler, W. R.; Oliver, P. A.; Parekh, S. I.; Patel, H. H.; Seif, L. S.; Staeger, M. A.; Wayne, G. S.; Wittenberger, S. J.; Zhang, W. A Scaleable Synthesis of Fiduxosin. *Org. Process Res. Dev.* **2004**, *8*, 897–902.
9. Liu, J.; Tang, W.; Sheng, L.; Du, Z.; Zhang, T.; Su, X.; Zhang, S. X.-A. Effects of substituents on metastable-state photoacids: Design, synthesis, and evaluation of their photochemical properties. *Chem. Asian J.* **2019**, *14*, 438–445.
10. Hajjaj, F.; Tashiro, K.; Nikawa, H.; Mizorogi, N.; Akasaka, T.; Nagase, S.; Furukawa, K.; Kato, T.; Aida, T. Ferromagnetic Spin Coupling between Endohedral Metallofullerene La@C_{82} and a Cyclodimeric Copper Porphyrin upon Inclusion. *J. Am. Chem. Soc.* **2011**, *133*, 9290–9292.
11. Hristova, Y. R.; Smulders, M. M. J.; Clegg, J. K.; Breiner, B.; Nitschke, J. R. Selective anion binding by a "Chameleon" capsule with a dynamically reconfigurable exterior. *Chem. Sci.* **2011**, *2*, 638–641.
12. Sheldrick, G. M. *SHELXT* - Integrated space-group and crystal-structure determination. *Acta Crystallogr. A* **2015**, *71*, 3–8.
13. Sheldrick, G. M. Crystal structure refinement with *SHELXL*. *Acta Crystallogr. C* **2015**, *71*, 3–8.
14. Dolomanov, O. V.; Bourhis, L. J.; Gildea, R. J.; Howard, J. A. K.; Puschmann, H. *OLEX2*: a complete structure solution, refinement and analysis program. *J. Appl. Crystallogr.* **2009**, *42*, 339–341.
15. Spek, A. L. *PLATON* SQUEEZE: a tool for the calculation of the disordered solvent contribution to the calculated structure factors. *Acta Crystallogr. C* **2015**, *71*, 9–18.
16. Avram, L.; Iron, M. A.; Bar-Shir, A. Amplifying undetectable NMR signals to study host–guest interactions and exchange. *Chem. Sci.* **2016**, *7*, 6905–6909.
17. Zaiss, M.; Bachert, P. Exchange-dependent relaxation in the rotating frame for slow and intermediate exchange – modeling off-resonant spin-lock and chemical exchange saturation transfer. *NMR Biomed.* **2013**, *26*, 507–518.
18. Zaiss, M.; Zu, Z.; Xu, J.; Schuenke, P.; Gochberg, D. F.; Gore, J. C.; Ladd, M. E.; Bachert, P. A combined analytical solution for chemical exchange saturation transfer and semi-solid magnetization transfer. *NMR Biomed.* **2015**, *28*, 217–230.
19. Becke, A. D. Density‐functional thermochemistry. III. The role of exact exchange. *J. Chem. Phys.* **1993**, *98*, 5648–5652.
20. Lee, C.; Yang, W.; Parr, R. G. Development of the Colle-Salvetti correlation-energy formula into a functional of the electron density. *Phys. Rev. B* **1988**, *37*, 785–789.

21. Frisch, M. J.; Trucks, G. W.; Schlegel, H. B.; Scuseria, G. E.; Robb, M. A.; Cheeseman, J. R.; Scalmani, G.; Barone, V.; Petersson, G. A.; Nakatsuji, H.; Li, X.; Caricato, M.; Marenich, A. V.; Bloino, J.; Janesko, B. G.; Gomperts, R.; Mennucci, B.; Hratchian, H. P.; Ortiz, J. V.; Izmaylov, A. F.; Sonnenberg, J. L.; Williams-Young, D.; Ding, F.; Lipparini, F.; Egidi, F.; Goings, J.; Peng, B.; Petrone, A.; Henderson, T.; Ranasinghe, D.; Zakrzewski, V. G.; Gao, J.; Rega, N.; Zheng, G.; Liang, W.; Hada, M.; Ehara, M.; Toyota, K.; Fukuda, R.; Hasegawa, J.; Ishida, M.; Nakajima, T.; Honda, Y.; Kitao, O.; Nakai, H.; Vreven, T.; Throssell, K.; Montgomery, J. A., Jr.; Peralta, J. E.; Ogliaro, F.; Bearpark, M. J.; Heyd, J. J.; Brothers, E. N.; Kudin, K. N.; Staroverov, V. N.; Keith, T. A.; Kobayashi, R.; Normand, J.; Raghavachari, K.; Rendell, A. P.; Burant, J. C.; Iyengar, S. S.; Tomasi, J.; Cossi, M.; Millam, J. M.; Klene, M.; Adamo, C.; Cammi, R.; Ochterski, J. W.; Martin, R. L.; Morokuma, K.; Farkas, O.; Foresman, J. B.; Fox, D. J. Gaussian 16, Revision C.01. Gaussian, Inc., Wallingford CT, 2016.
